# Molecular detection and genomic characterization of diverse hepaciviruses in African rodents

**DOI:** 10.1093/ve/veab036

**Published:** 2021-04-12

**Authors:** Magda Bletsa, Bram Vrancken, Sophie Gryseels, Ine Boonen, Antonios Fikatas, Yiqiao Li, Anne Laudisoit, Sebastian Lequime, Josef Bryja, Rhodes Makundi, Yonas Meheretu, Benjamin Dudu Akaibe, Sylvestre Gambalemoke Mbalitini, Frederik Van de Perre, Natalie Van Houtte, Jana Těšíková, Elke Wollants, Marc Van Ranst, Oliver G Pybus, Jan Felix Drexler, Erik Verheyen, Herwig Leirs, Joelle Gouy de Bellocq, Philippe Lemey

**Affiliations:** 1 Department of Microbiology, Immunology and Transplantation, Rega Institute, KU Leuven, Leuven, Belgium; 2 Department of Biology, Evolutionary Ecology Group, University of Antwerp, Antwerp, Belgium; 3 EcoHealth Alliance, New York, NY, USA; 4 Institute of Vertebrate Biology of the Czech Academy of Sciences, Brno, Czech Republic; 5 Pest Management Center –Sokoine University of Agriculture, Morogoro, Tanzania; 6 Department of Biology and Institute of Mountain Research & Development, Mekelle University, Mekelle, Ethiopia; 7 Department of Ecology and Animal Resource Management, Faculty of Science, Biodiversity Monitoring Center, University of Kisangani, Kisangani, Democratic Republic of the Congo; 8 Department of Botany and Zoology, Faculty of Science, Masaryk University, Brno, Czech Republic; 9 Department of Zoology, University of Oxford, Oxford, UK; 10 Department of Pathobiology and Population Sciences, The Royal Veterinary College, London, UK; 11 Charite—Universitatsmedizin Berlin, Berlin, Germany; 12 German Center for Infection Research (DZIF), Berlin, Germany; 13 OD Taxonomy and Phylogeny—Royal Belgian Institute of Natural Sciences, Brussels, Belgium

**Keywords:** rodent hepacivirus, Hepatits C virus, cross-species transmission, hepacivirus co-infection, recombination

## Abstract

Hepatitis C virus (HCV; genus *Hepacivirus*) represents a major public health problem, infecting about three per cent of the human population. Because no animal reservoir carrying closely related hepaciviruses has been identified, the zoonotic origins of HCV still remain unresolved. Motivated by recent findings of divergent hepaciviruses in rodents and a plausible African origin of HCV genotypes, we have screened a large collection of small mammals samples from seven sub-Saharan African countries. Out of 4,303 samples screened, eighty were found positive for the presence of hepaciviruses in twenty-nine different host species. We, here, report fifty-six novel genomes that considerably increase the diversity of three divergent rodent hepacivirus lineages. Furthermore, we provide strong evidence for hepacivirus co-infections in rodents, which were exclusively found in four sampled species of brush-furred mice. We also detect evidence of recombination within specific host lineages. Our study expands the available hepacivirus genomic data and contributes insights into the relatively deep evolutionary history of these pathogens in rodents. Overall, our results emphasize the importance of rodents as a potential hepacivirus reservoir and as models for investigating HCV infection dynamics.

## 1. Introduction

Diseases originating from animal sources represent a tremendous public health threat that requires sustained research effort and informed intervention measures. Owing to major advances in genome sequencing technologies, we are now able to characterize pathogen emergence and explore the interplay between viral evolution and host ecological dynamics in great detail. By obtaining viral genetic data and applying evolutionary analysis methods, we can determine key factors for interspecies transmissions and successful epidemic spread in the human population.

The current COVID-19 pandemic, the recent Ebola virus outbreaks and the 2009 influenza H1N1 pandemic are just three examples that highlight the need to understand zoonotic disease emergence. To date, we still lack essential knowledge of how viruses evolve from their reservoir species, emerge into the human population and establish infections with epidemic and/or even pandemic potential. There is not only a pressing need to address these questions for recently emerged diseases but also important is to understand the origins of long-established human pathogens.

Viruses in the genus *Hepacivirus* (family *Flaviviridae*) are positive-sense single-stranded RNA viruses. Hepatitis C virus (HCV), the type species of this genus, was discovered in 1989 (Choo et al. 1989) and is an important blood-borne human pathogen that can cause severe chronic liver disease with more than 185 million infections globally (Messina et al. 2015). Although considerable research has been devoted to the optimization of curative antivirals, comparatively less effort has been put into unravelling the epidemic history and emergence of HCV. A zoonotic origin several hundred years ago has been postulated by Pybus and Thézé (2016), but convincing evidence for this remains lacking.

For a long time, HCV was the sole representative of the *Hepacivirus* genus. Since 2011, however, efforts to fill the gaps in hepacivirus diversity led to the identification of HCV homologues in a wide range of animal hosts. To date, those include mammalian hosts such as bats (Quan et al. 2013), cows (Baechlein et al. 2015; Corman et al. 2015), dogs (Kapoor et al. 2011; El-Attar et al. 2015), horses (Burbelo et al. 2012; Lyons et al. 2012), non-human primates (Lauck et al. 2013; Canuti et al. 2019; Porter et al. 2020), possums (Chang et al. 2019), shrews (Guo et al. 2019), sloths (Moreira-Soto et al. 2020) and rodents (Drexler et al. 2013; Kapoor et al. 2013; Firth et al. 2014; Van Nguyen et al. 2018). Non-mammalian hosts harbouring hepaciviruses have also been identified in birds (Chu et al. 2019; Goldberg et al. 2019; Porter et al. 2020), fish and reptiles (Shi et al. 2018). Finally, divergent hepaciviruses were very recently detected in the first non-vertebrate hosts, specifically in a *Culex annulirostris* mosquito (Williams et al. 2020) and an *Ixodes holocyclus* tick species (Harvey et al. 2018).

Despite our expanding knowledge of the hepaciviral host range, the zoonotic origins of HCV still remain unresolved. The most closely related viruses to HCV have been identified in horses (Burbelo et al. 2012; Lyons et al. 2012) and this lineage has also been introduced in dogs (Pybus and Thézé 2016) and donkeys (Walter et al. 2017). Very recently, a hepaci-like virus sampled from a Senegal bushbaby was shown to group with the equine lineage (Porter et al. 2020). Rodent hepaciviruses (RHVs) show the greatest genetic heterogeneity among mammalian host clades and were hypothesized to constitute the primary zoonotic source of mammalian hepaciviruses (Pybus and Gray 2013; Hartlage et al. 2016; Pybus and Thézé 2016). The idea that rodents could be reservoir from which viruses have been introduced to other animals has gained considerable momentum. Not only do rodents host extensive virus diversity but also are abundant in nature, which provides them with ample ecological opportunity for spreading infectious diseases (Pybus and Thézé 2016).

Although hepaciviruses have now been identified in a variety of hosts, our knowledge about the evolutionary dynamics of hepaciviruses is almost entirely based on HCV. It still remains unclear to what extent insights from HCV can be extended or applied to other hepaciviruses. Recombination is, for example, rare in HCV, but Thézé et al. (2015) suggested that it may have occurred in the ancestral history of different hepacivirus lineages. HCV is also known to escape immune responses during chronic infection (Gaudieri et al. 2006), but whether similar virus–host interactions impact hepacivirus evolution in other hosts is currently unknown. Finally, while the HCV genome has been estimated to evolve at about $10^{-3}$ substitutions/site/year (Gray et al. 2011), a rate typical of RNA viruses, similar attempts to quantify the evolutionary rate of hepaciviruses in other hosts are lacking.

To scrutinize the role of small mammals as potential natural reservoir hosts of hepaciviruses, we screened 4,303 specimens from wild mammals corresponding to 161 species. We specifically focused on rodents that accounted for the majority of our sample collection. Complementary to previous research that mainly investigated rodents from Europe and the New World (Drexler et al. 2013; Kapoor et al. 2013; Firth et al. 2014), our sampling concentrated exclusively on Africa. The focus on sub-Saharan Africa as a source of HCV is critical because it harbours several endemic HCV genotypes. Using a set of new RHV genomes, we characterize diversity, virus–host phylogenetic relationships and co-infection patterns. Finally, we take an important step towards exploring the evolutionary dynamics of hepaciviruses by examining recombination, selective pressure and temporal signal in specific host lineages.

## 2. Materials and methods

### 2.1 Sample collection and hepacivirus screening

We screened a large collection of small mammal samples that was assembled from various ecological and evolutionary studies by authors of this study and their collaborators (Laudisoit et al. 2009; Goüy de Bellocq et al. 2010; Meheretu et al. 2012; Massawe et al. 2012; Bryja et al. 2012, 2014; Gryseels et al. 2015, 2017; Makundi et al. 2015; Těšíková et al. 2017; Mazoch et al. 2018; Petružela et al. 2018; Van de Perre et al. 2018, 2019b). As part of these studies, a total number of 4,303 wild mammals (rodents, bats, shrews, elephant shrews, hedgehogs and moles) were captured in multiple localities of seven different countries across Central and East Africa between 2006 and 2013. The specimen collection used here consists of 894 animals originating from the Democratic Republic of the Congo (DRC), 426 from Ethiopia, 532 from Kenya, 30 from Madagascar, 399 from Mozambique, 1,798 from Tanzania and 224 from Zambia (Supplementary Table S1). For the majority of captured individuals (using various types of traps), whole blood was collected on pre-punched filter papers but also spleen, kidney and other organs were collected and stored in RNAlater (Qiagen) at −20*°*C or in ethanol at room temperature.

For hepacivirus screening, *n *=* *4, 173 dried blood spots (DBS) were pooled by two and RNA was extracted using the RTP DNA/RNA Virus Mini Kit (Stratec), according to the manufacturer’s instructions and using the maximum lysis incubation time. For *n *=* *130 kidneys (all belonging to the collection from Mozambique), RNA was purified using the Nucleospin RNA II Total RNA Isolation kit (Macherey-Nagel) following the manufacturer’s protocol. Complementary DNA (cDNA) was synthesized from either blood or tissue extracts using Maxima Reverse Transcriptase (ThermoFisher Scientific) with random hexamers and 8 μl of RNA extract.

In order to screen for hepaciviruses, we employed a hemi-nested polymerase chain reaction (PCR) assay targeting a 300-nt fragment of the conserved NS3 protease-helicase gene, as described by Kapoor et al. (2013). The first round of PCR was performed using the OneStep RT-PCR Kit (Qiagen) with primer pair AK4340F1 and AK4630R1 (Kapoor et al. 2013) and 5 μl of cDNA. The cycling conditions consisted of an additional reverse transcription step with a sequence-specific primer at 50 *°*C for 30 minutes, followed by an initial denaturation step at 95 °C for 15 minutes. The PCR cycle included thirty-five rounds of 95 °C for 30 seconds, 57 °C for 30 seconds and 72 °C for 1 minute. The final extension step was performed at 72 °C for 10 minutes. For the second round of PCR, 1 μl of the amplified product was subjected to another PCR reaction using primer pair AK4340F2 and AK4630R2 (Kapoor et al. 2013) along with the DreamTaq DNA Polymerase (ThermoFisher Scientific). The PCR conditions included 3 minutes of denaturation at 95 °C, forty cycles of 95 °C for 20 seconds, 62 °C for 20 seconds, 72 °C for 30 seconds and a final extension step of 10 minutes at 72 °C. The quality of the PCR products was assessed visually through gel electrophoresis and in case of reasonable indication of hepacivirus presence, we subsequently purified the PCR product using the ExoSAP-IT PCR Product Cleanup Reagent (Applied Biosystems) and performed Sanger sequencing. Positive hepacivirus hits were confirmed through a similarity search against a custom viral database employing the tBLASTx algorithm.

We resolved the positive pools by individually extracting RNA from the separate DBS samples or from either kidney or spleen, depending on availability of the biological material for each individual. On these tissue extracts, an additional screening step was performed using the previously described PCR assay in order to confirm hepacivirus presence.

### 2.2 Whole-genome sequencing and hepacivirus genome assembly

We focused our available resources on obtaining viral genomic data from rodent individuals and attempted whole genome sequencing on the positive specimens when organ samples were available. Total RNA was purified from kidneys and spleens stored in RNAlater (Qiagen) at −20 *°*C or from other organs stored in ethanol at room temperature, using an optimized protocol for the RNeasy Mini Kit (Qiagen). In order to optimally prepare the specimens for whole-genome sequencing, we adapted the original assay as detailed by Bletsa et al. (2019). Briefly, we introduced two freeze–thaw steps: before and after tissue homogenization, followed by an intermediate on-column DNase treatment using the RNase-Free DNase Set (Qiagen) to remove any residual DNA prior to RNA purification. In order to increase the yield of viral RNA during extraction, we used the flow-through from the first elution to re-elute the column.

RNA extracts were subjected to RNA quantification using the RNA Quantifluor System (Promega) and their RNA profiles were assessed on an Agilent RNA 6000 Nano chip (Agilent Technologies). Prior to library preparation, a ribosomal RNA (rRNA) depletion step using the Ribo-Zero rRNA Removal Kit (Illumina) was applied to the total RNA to eliminate both cytoplasmic and mitochondrial rRNAs. For cDNA generation and construction of the sequencing libraries, we used the NEXTflex Rapid Illumina Directional RNA-Seq Library Prep Kit (PerkinElmer) followed by paired-end sequencing on an Illumina NextSeq 500 at Viroscan3D (Lyon, France).

Demultiplexing was performed using Bcl2fastq v2.17.1.14 (Illumina) and low-quality parts of the reads were trimmed using Trimmomatic v0.36 (Bolger et al. 2014). For digital host subtraction we used SNAP v1.0 (Zaharia et al. 2011) to map the trimmed reads against a list of ten mammalian reference genomes (eight rodent species, shrew and human genome) coupled with PRINSEQ v0.20.4 (Schmieder and Edwards 2011) as an additional filtering step prior to *de novo* assembly. SPAdes v3.12.0 (Bankevich et al. 2012) was used to generate contigs, which were subsequently analysed using tBLASTx against a flavivirus- and hepacivirus-enriched database. To correct for any sequence polymorphism, we re-mapped all reads to our generated consensus sequences using Bowtie2 v2.2.5 (Langmead and Salzberg 2012) and QUASR v6.08 (Watson et al. 2013). Coverage and sequencing depth were assessed by calculating the proportion of the mapped reads over the total numbers of reads (see Supplementary Table S2).

To fill gaps in partial genomes, we designed strain-specific primers (see Supplementary Table S3) and generated overlapping amplicons using the OneStep RT-PCR Kit (Qiagen) and 5 μl of cDNA. PCR products were purified and Sanger sequenced in both directions. Open reading frames were predicted in Geneious Prime v2019.2.1 (Biomatters 2019) based on previously characterized RHVs.

### 2.4 Validation of co-infections

To exclude the possibility of *de novo* assembly artefacts in co-infection detection, we developed a strain-specific PCR validation assay for two different specimens (MOZ329 and TA100) that harboured three RHVs each. Outer and inner primer pairs were designed targeting the most variable region of the RHV genome (see Supplementary Fig. S1 and Supplementary Table S3). All PCR reactions were performed using the OneStep RT-PCR Kit (Qiagen) with only very few differences compared with the detection assay. For the first round of PCR, an annealing temperature of 52 °C was used, whereas 53 °C was the optimal temperature for the second round. Products were loaded on a two per cent agarose gel and the appropriate bands were excised and cleaned up with the Zymoclean Gel DNA Recovery Kit (Zymo Research). All purified amplicons were shipped to Macrogen Europe B.V. (Amsterdam, the Netherlands) and Sanger sequenced in both directions. Sequencing chromatographs were visually checked and the sequences were mapped to their corresponding strains. As an additional step to validate the co-infections, we meticulously tested for intra-specific recombination using SimPlot v3.5.1 (Lole et al. 1999) and BootScan methods (Salminen et al. 1995; Martin et al. 2005) with the default settings of a 200 bp window size and a step size of 20 bp.

### 2.4 Phylogenetic analysis and visualization

We analysed our novel rodent (*n *=* *56) and bat (*n *=* *2) hepacivirus genomes together with all available full-length hepaciviruses (*n *=* *130) in GenBank (accessed on 10 January 2021) along with information on their host, sampling location and collection date. Due to the vast number of sequences available for HCV, we only included one representative genome from each HCV genotype. This resulted in a final dataset of 188 genome-wide hepaciviruses (Supplementary Table S4). All 5′ and 3′ untranslated regions were removed to retain the polyprotein coding sequence for downstream analyses.

Upon translating the polyprotein sequences to amino acid sequences, we built a multiple alignment using MAFFT v7.407 (Katoh et al. 2009) and SeaView v4.6 (Gouy et al. 2010) in a stepwise approach. First, we generated alignments for all main lineages defined by Thézé et al. (2015): equine, bovine, human, primate, bat and rodent virus lineages. Second, we manually edited the individual alignments in Aliview v1.18.1 (Larsson 2014) to remove large gaps and then progressively incorporated the lineage-specific alignments into a single multiple host alignment using profile alignment. BMGE v1.12 (Criscuolo and Gribaldo 2010) was used to eliminate ambiguously aligned regions from our dataset (188 sequences, 1,696 amino acids).

We used IQ-TREE v1.6.7 (Nguyen et al. 2015) to find the best-fitting amino acid substitution model according to the Bayesian Information Criterion (BIC), which was identified to be the LG + F + I + G4 model, and to reconstruct maximum likelihood (ML) phylogenies using this substitution model. We obtained bootstrap support using 1,000 pseudo-replicates and visualized trees as midpoint-rooted.

Amino acid alignments for the classification of hepaciviruses were prepared according to the methodology proposed by Smith et al. (2016), which resulted in a subset of sixty sequences. We estimated mean pairwise amino acid *p*-distances using MEGA7 v.0.1 (Kumar et al. 2016) for positions 1123–1566 in NS3 and 2536–2959 in NS5B. Genome positions were numbered relative to the M62321 reference genome (Choo et al. 1989). Phylogenetic trees were reconstructed for both regions with IQ-TREE v1.6.7 (Nguyen et al. 2015) using the LG + F + I + G4 substitution model and 1,000 bootstrap replicates.

To molecularly confirm the host species of the positive specimens, we recovered cytochrome b gene sequences from the samples subjected to whole-genome sequencing by directly mapping the deep sequencing data to a list of reference sequences from various African rodent species. Some of these species have not yet been formally named, but we used expert opinion to delineate the different species. In addition, we downloaded cytochrome b sequence data for the twelve rodent species from which hepacivirus genomes were sequenced in previous studies (see Supplementary Table S5).

Phylogenetic trees based on the alignment of twenty-one cytochrome b sequences were estimated with IQ-TREE v1.6.7 (Nguyen et al. 2015) using the TIM2 + F + I + G4 nucleotide substitution model (identified as the best model according to the BIC) and clade support was assessed using 1,000 bootstrap replicates.

To visualize and annotate phylogenies we made use of *ggtree* and *treeio* R packages (Yu et al. 2018; Wang et al. 2019). In order to investigate the relationships between rodents and hepaciviruses we created a co-phylogenetic plot (or ‘tanglegram’). This visual representation plots the host phylogeny opposite to the virus phylogeny and draws lines between the taxa of the two trees, as a function of their topological distance. Here, we focused on highlighting the evolutionary relationships of rodent-borne hepaciviruses and their hosts only, as those mammals were exclusively associated with multiple circulating hepacivirus strains (co-infections). Briefly, for the tanglegram we constructed a viral phylogeny based on a subset of all RHVs (*n *=* *82) from the large dataset using the approach described above. The association matrix between the host and viral phylogeny was computed using the *ape* R package (Paradis and Schliep 2019) and patristic distances were calculated using the *adephylo* R package (Jombart et al. 2010).

### 2.5 Recombination, selective pressure and temporal signal in host-specific lineages

For comparative evolutionary analyses, we selected viral genomes representative for specific host lineages from the complete hepacivirus phylogeny that are roughly similar in diversity (see Supplementary Fig. S2). This includes the entire collections of bovine (*n *=* *16) and equine (*n *=* *34) strains and subsets of two rodent lineages (*n *=* *11 and *n *=* *36, respectively). To represent HCV, we collected representative genome data sets of similar sizes for HCV genotype 1a (*n *=* *35), genotype 1 b (*n *=* *34) and genotype 3a (*n *=* *34) by applying phylogenetic diversity analyser (Chernomor et al. 2015) to the large number of genomes publicly available in Genbank.

Multiple sequence alignments were constructed for the amino acid translations of the polyprotein coding sequence using Muscle v3.8.31 (Edgar 2004) and back-translated to the nucleotide sequences. A relatively short but highly diverse part in the 3′ region of one of the rodent lineage alignments was removed by manual editing. To ensure comparable data in the evolutionary analyses, the equivalent part was removed from the other host-lineage alignments.

We tested for recombination in the host-specific lineages using the PHI-test (v4.15.1) (Bruen et al. 2006) and confirmed the evidence of significant recombination using RDP4 v4.97 (Martin et al. 2015). The RDP analysis employed the following individual methods: 3SEQ (Lam et al. 2018), RDP (Martin and Rybicki 2000), Bootscan (Martin et al. 2005), Chimaera (Martin et al. 2005) and SisScan (Martin et al. 2005). For RDP, Bootscan and SisScan, a window size of 200 bp was selected, while for Chimaera, we allowed for a number of twenty variable sites per window. Apart from specifying linear genomes and recombination events to be listed by more than two methods, all other parameters were kept to their default settings.

Due to the detection of a significant amount of recombination in the viral genomes from animal hosts, we estimated selection pressure at the molecular level using the population genetics approach implemented in omegaMap v0.5 (Wilson and McVean 2006). This method was specifically designed to estimate the relative excess of nonsynonymous (dN) over synonymous (dS) substitutions in the presence of recombination. We performed selection analyses on the same data sets (see Supplementary Fig. S2) that were analysed for recombination. In our analyses, we allowed for variation in dN/dS ratio according to a block-like model with length of thirty sites. We set the codon frequencies to the values obtained by multiplying the four empirical nucleotide frequencies that were obtained separately for the three codon positions. We summarize mean estimates as well as 95 per cent highest posterior density (HPD) intervals for the site-specific dN/dS values. We consider high dN/dS estimates to be significantly higher than 1 if their 95 per cent HPD intervals do not include 1.

To test for temporal signal, we focused on the genomes in the lineage-specific data sets for which sampling time was available. This information was retrieved for all HCV genomes, the rodent virus genomes (exact sampling dates), and for fourteen out of sixteen bovine virus genomes, as well as for twenty-two out of thirty-four equine virus genomes. To avoid the impact of recombination, minor recombinant regions were masked based on the RDP4 analysis, keeping only the major non-recombinant regions in the lineage-specific alignments. Temporal signal was explored in a visual manner and tested using a Bayesian inference procedure. For the visual assessment, we plotted root-to-tip divergences as a function of sampling time using TempEst v1.5.3 (Rambaut et al. 2016) based on ML trees inferred by IQ-TREE under a GTR + G4 nucleotide substitution model. A more formal test of temporal signal was performed by comparing marginal likelihood estimates for a model with dated tips and a model that assumes all sequences are contemporaneous (Duchene et al. 2019).

## 3. Results

### 3.1 Hepaciviruses are present in a wide range of rodent host species

We screened *n *=* *4, 303 samples from small mammals, collected between 2006 and 2013 in Central and East Africa, for the presence of hepaciviruses. Most of the specimens were from rodents (*n *=* *3,788) representing 38 genera and 116 potential rodent species. In addition to rodent specimens, *n *=* *515 samples from shrews, bats, elephant shrews, hedgehogs and moles were screened for hepaciviruses (see also Supplementary Table S1).

**Figure 1.**
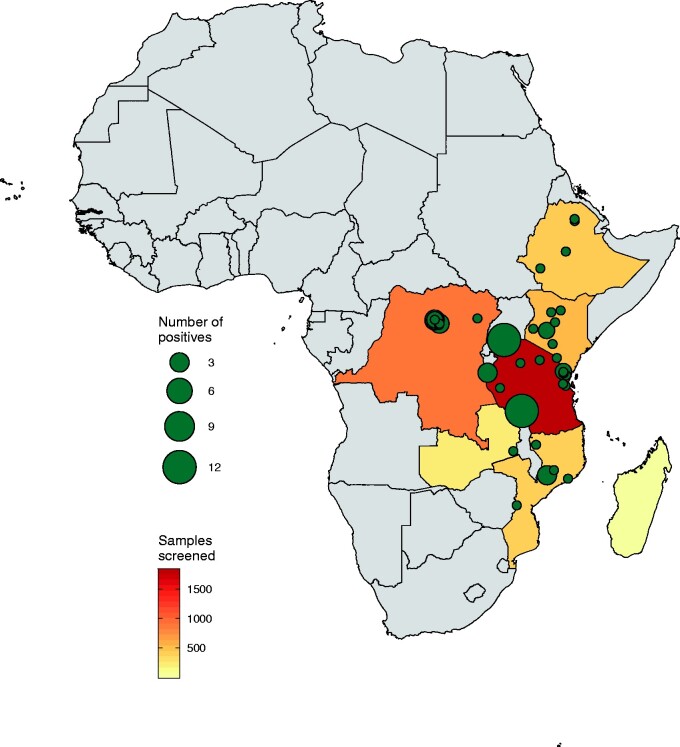
Spatial distribution of the hepacivirus-positive specimens. Map of Africa indicating sampling sites and the exact locations of our detected hepacivirus cases. Grey-coloured countries correspond to locations that were not included in this survey, while coloured countries represent our sampling focus. In those countries, the number of specimens screened is indicated by a continuous colour scale ranging from yellow (small sampling size) to red (large sampling size). Green circles denote the number of hepaciviruses detected in each locality. Circle sizes are proportional to the number of infected individuals, ranging from one to twelve positive specimens per site.

Out of the eighty PCR-positive specimens, two were identified in a single bat species and seventy-eight in twenty-eight potential rodent species. We did not detect any positive shrew samples, although these mammals have been previously reported to host hepaciviruses (Guo et al. 2019) (Supplementary Fig. S3, Supplementary Table S6). In Fig. 1, we mapped the screening results to further investigate the geographic distribution of the positive specimens. This highlights three distinct localities with a relatively high number of RHV infections: two sites in Tanzania and one in Mozambique.

### 3.2 Evolutionary relationships of hepaciviruses with a focus on rodent hosts

We generated fifty-six complete hepacivirus genomes originating from nine rodent species and two complete bat hepaciviruses from a single host species using an untargeted deep sequencing procedure. The mean read depth for the fifty-eight genomes was 433, with a 20× coverage for more than 93.1 per cent of each genome (Supplementary Table S2).

**Figure 2.**
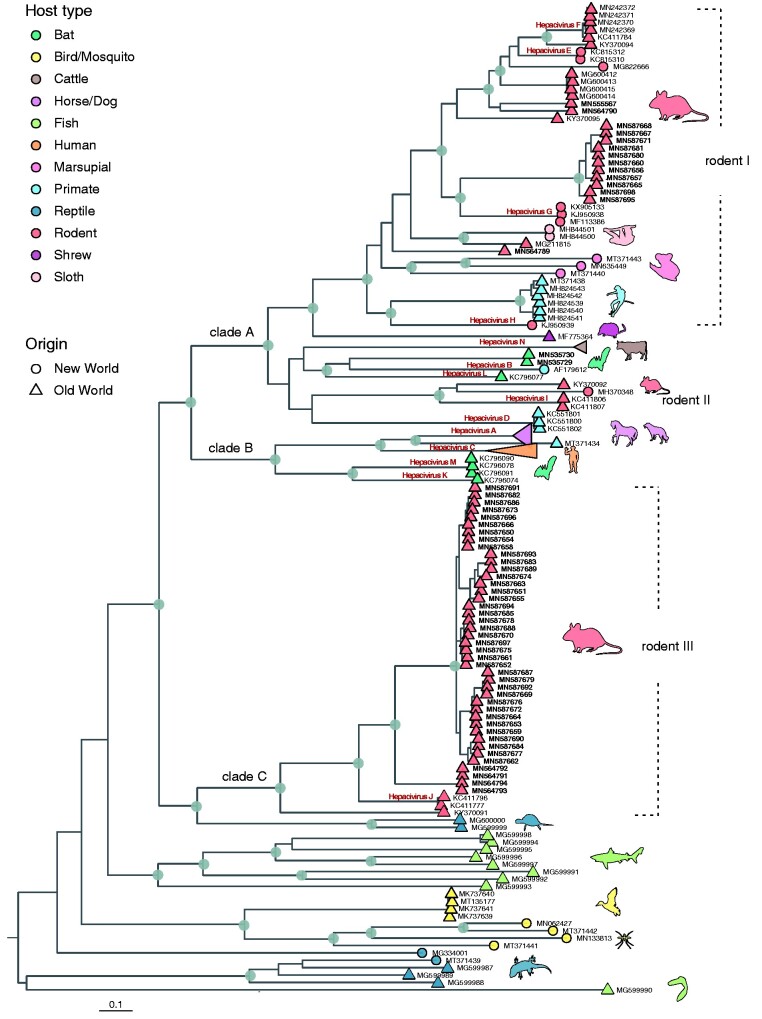
Genome-wide phylogenetic reconstruction of hepaciviruses. ML tree of all available (*n *=* *130) and novel (*n *=* *58) hepacivirus genomes. Silhouettes indicate hosts and are coloured according to their broader host type: bats (green), birds and mosquito (yellow), cattles (brown), equids and dog (lilac), fishes (lime green), humans (peach orange), marsupials (pastel pink), primates (light blue), reptiles (steel blue), rodents (salmon), shrew (plum) and sloths (champagne pink). Grey circles indicate internal nodes with bootstrap support ≥70, while designated virus species names are shown in bold bordeaux text. Circles at the tips denote New World origin, while triangles represent the Old Wolrd viruses. Novel genomes generated in this study are labeled with bold text. Clades A, B and C have been provisionally named for the purpose of discussing the mammalian hepacivirus lineages in the main text.

Phylogenetic analysis of a data set that combines all available hepacivirus genomes with our new data confirms an extensive virus diversity that is to some extent structured by vertebrate class, order and family into host-specific clades (Fig. 2). All mammalian hepaciviruses form a monophyletic cluster, but with low bootstrap support. Bird, fish and reptile hepaciviruses form lineages basal to the mammalian-borne clade and exhibit long branches, which suggests long-term circulation in these hosts. Within the basal lineages, bird hepaciviruses form a monophyletic cluster assuming that a virus identified in a mosquito that had fed on a bird is indeed a bird virus (Williams et al. 2020) (Fig. 2).

We distinguish three well-supported clades (provisionally named A, B and C) in mammals for the purpose of description. Clade A represents the most heterogenous group and is characterized by an intermingling of viruses found in a wide range of taxonomically diverse animals such as rodents, bats, shrews, sloths, non-human primates, possums and cattle. Clade B contains viruses originating from equine, canine, human and bat hosts, while clade C comprises a strictly monophyletic group of RHVs. Previously identified bat hepaciviruses (BHVs) form two distinct lineages with few representatives that cluster in both clades A and B. Our two new BHVs constitute a new lineage that groups with a virus from a tamarin host, with one of the previous African BHV falling as a sister-lineage to this clade. RHVs fall in three divergent lineages in the phylogeny (Fig. 2). Two separate RHV lineages can be identified in clade A, while the remaining lineage is responsible for the entire C clade. In further analyses below, we refer to the rodent-borne lineages in clade A as ‘rodent I’ and ‘rodent II’, containing viruses that are paraphyletic with respect to non-human primate, bat, shrew, sloth and possum viruses, and to the third RHV lineage as ‘rodent III’ (equivalent to clade C).

The ever-expanding genomic characterization of hepaciviruses is challenging to subject to systematic classification. To illustrate this, we attempted to apply the classification criteria by Smith et al. (2016), which focus on two relatively conserved subgenomic regions: part of NS3 and NS5B (Supplementary Table S7). This resulted in an impractically large number of different virus ‘species’, many of which were represented by only a single taxon, and a large degree of inconsistency between the two genome regions despite congruent tree topologies (Supplementary Fig. S4). It therefore remains more practical and coherent to describe lineages by strongly supported evolutionary units, which is also in line with the interspecific level previously used by Thézé et al. (2015).

As demonstrated in Supplementary Fig. S5, our novel hepaciviruses fall into the major rodent lineages that were previously disproportionally represented by New-World and Asian viruses. More specifically, the diverse rodent I cluster did not include any African viruses, while the rodent II lineage contained only two African genomes. In addition, rodent III clade was exclusively represented by European and Asian viruses. The large number of African hepacivirus genomes that contribute about seventy per cent of the current RHV genomes increases the Old-World diversity of those lineages and substantially broadens the known host spectrum of RHVs, especially in the Muridae family. Of particular interest is an isolate that originated from a *Graphiurus kelleni* sample collected in the DRC (GenBank accession number MN564789). This rodent species belongs to the Gliridae family, which has not been included in any of the previous screening efforts. The closest relative of this strain is a hepacivirus from a *Spermophilus dauricus* sample that was isolated in China, indicating the deep evolutionary trajectory of RHVs.

In terms of geographic structure, the rodent I and II lineages show an intermingling of hepaciviruses from different continents without any clear patterns of co-divergence between the viruses and their rodent hosts. In contrast, the rodent III lineage (or clade C) exhibits some degree of confinement to specific rodent taxa since one subclade of this lineage is restricted to the *Lophuromys* genus and another subclade is restricted to the Praomyini tribe (Lecompte et al. 2008). Hepaciviruses in the rodent III lineage are exclusively sampled from Old-World locations and demonstrate geographic clustering by continent, but with mixing by country in Africa (Supplementary Fig. S5). The large diversity and wide distribution of RHVs as well as the lack of a clear geographic and host structure suggest a relatively long-term circulation history with little boundaries to transmission among different rodent hosts.

### 3.3 Hepacivirus co-infections within *Lophuromys* rodents

**Figure 3.**
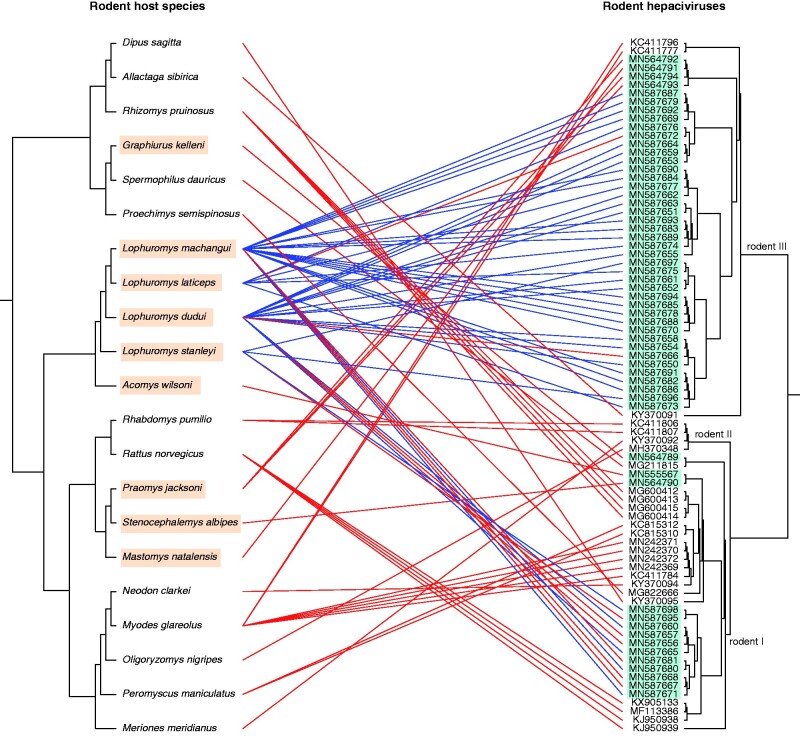
Tanglegram of rodent hosts and their hepaciviruses. The topology of the host tree was reconstructed using the cytochrome b gene from twenty-one rodent species (left phylogeny). For the viral reconstruction we used the rodent subset of our genome-wide alignments (right phylogeny) and we highlighted the novel RHV genomes in turqoise. Lines connecting the two phylogenies represent an association between the rodent host species and their identified hepaciviruses. Blue lines correspond to individuals harbouring multiple hepaciviruses, while rodent species highlighted in a caramel colour represent the novel hosts found to be hepacivirus positive.

We identified a large proportion of RHV-positive samples harbouring multiple divergent strains, suggesting a relatively high degree of hepacivirus co-infections among those rodents. Specifically, eleven *Lophuromys* individuals were found to carry from two up to five different hepaciviruses, which—in some cases—clustered in different clades. Hepacivirus co-infections were not found in any other sampled genus (see also Supplementary Table S8). To exclude the possibility that these multiple genomes could have been generated by assembly artefacts, we performed additional molecular assays and computational analyses. The *in silico* validation included different assembly algorithms to *de novo* reconstruct our sequencing data. The majority of algorithms resulted in multiple hepacivirus strains per sample, albeit with variabilities in the length of the generated scaffolds. As *in vitro* validation test, we developed a strain-specific PCR assay to examine two cases harbouring three hepaciviruses each. Sanger sequencing following strain-specific PCR assays targeted to the hypervariable regions confirmed the presence of three distinct hepacivirus strains in each of the two tested specimens for which co-infections were inferred from metagenomic sequencing. This demonstrates that our metagenomic sequencing protocols and our bioinformatic pipeline indeed reliably retrieved distinct hepacivirus genomes (and thus co-infections) within single specimens. Phylogenetic relationships among those RHVs are depicted in (Fig. 3), while a summary of the multiple isolates per individual can be found in Supplementary Table S8.

Molecular identification of rodent hosts resulted in a wide species spectrum that can be broadly divided into three groups, as highlighted in Fig. 3: (1) those belonging to the Deomyinae subfamily: *Acomys wilsoni*, *Lophuromys dudui*, *Lophuromys laticeps*, *Lophuromys machangui* and *Lophuromys stanleyi*, (2) those belonging to the Murinae subfamily: *Mastomys natalensis*, *Praomys jacksoni* and *Stenocephalemys albipes* and (3) the one belonging to the *Graphiurus kelleni* species within the Gliridae family. Despite the broad host spectrum elucidated by our screening, a substantial proportion (55%) of hepaci-positive individuals were identified in the four species of *Lophuromys* rodents, and only these rodents were found to harbour more than one RHV strain. This is the first time that hepacivirus co-infections have been described in non-human host species. Figure 3 highlights the viruses found in co-infections (blue lines) in a virus–host phylogenetic comparison, indicating that all co-infections were associated with only four potential species of the *Lophuromys* genus. Comparison of the rodent host phylogeny to the corresponding RHV phylogeny does not demonstrate any appreciable co-divergence patterns (Fig. 3), suggesting again that these viruses have frequently jumped rodent hosts throughout their evolutionary history and that they may transmit relatively easily between different rodent species and genera under appropriate ecological opportunities.

### 3.4 Intraspecific recombination, prevailing negative selection and absence of temporal signal

With our additional RHV sampling, we assess recombination within host lineages (those lineages specific to a host type), where co-infections are more likely as indicated by our findings in the *Lophuromys* genus, and where high-sequence divergence is less of a cofounding factor. We performed comparative analyses on host-specific data sets with relatively limited and comparable genetic diversity (Supplementary Fig. S2). Formal testing using the PHI-test (Bruen et al. 2006) provided significant evidence for recombination in the bovine, equine, and the rodent I and III lineages (*P *<* *0.01), but not in the three HCV data sets (1a, 1b and 3a) we included for comparison.

A substantial number of intraspecific recombinants were identified in rodents, with the highest proportion in strains circulating in the rodent III lineage. However, we did not detect any significant evidence for recombination among RHV genomes within any of the co-infections we identified. These results were also confirmed by a variety of methods implemented in RDP4 (Martin et al. 2015). For more details on specific recombinants and mosaic patterns found in each host lineage, we refer to the Supplementary information.

**Figure 4.**
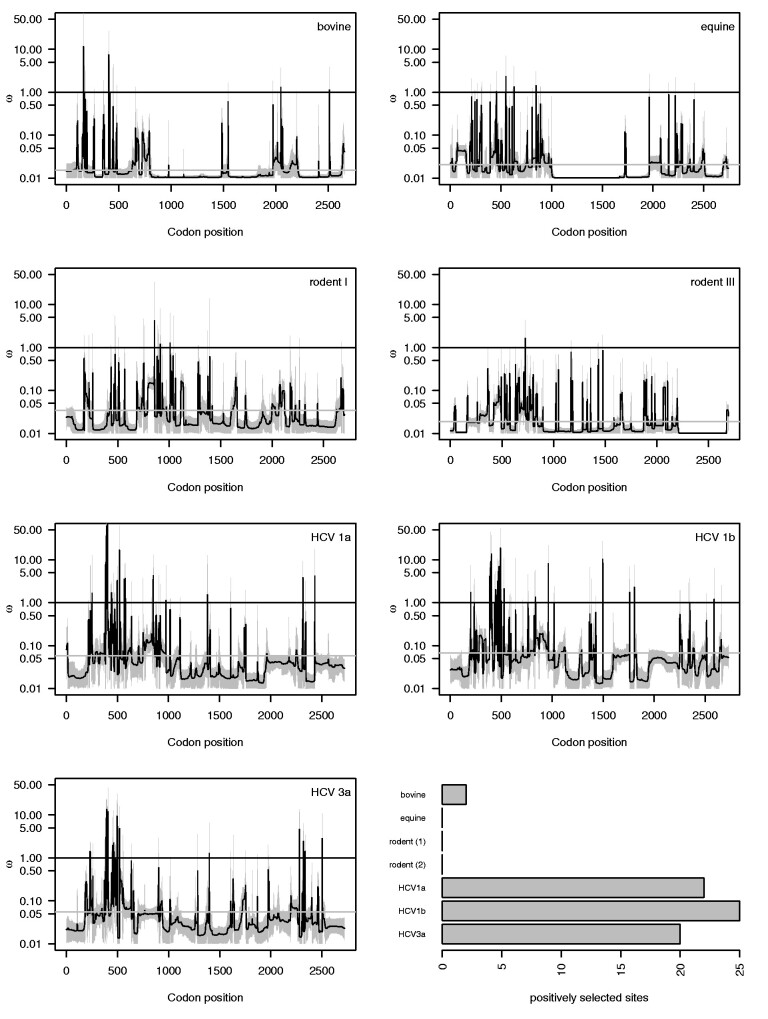
Site-specific variation of selection pressure in hepaciviruses. Estimates of ω in different animal hosts of hepaciviruses using omegaMap (Wilson and McVean 2006). Equine and RHVs show no positively selected sites across their genome; even though a few mean dN/dS estimates are above 1, their 95 per cent HPD intervals include 1. For bovine hepaciviruses only two sites evolve under positive selection. HCV genotypes 1a, 1b and 3a indicate statistically significant positive selection pressure in 22, 25 and 20 sites, respectively.

By focusing on specific host lineages, we can also perform genome-wide comparative analyses of selective pressure. At the interspecific level, such analysis would only be able to focus on conserved parts in which third codon positions may still suffer from saturation (Thézé et al. 2015). Because the presence of recombination within the specific bovine, equine and rodent I and III lineages tested complicates widely used phylogenetic codon substitution methods, we adopted a population genetic approach to estimate the ratio of nonsynonymous (dN) over synonymous (dS) substitutions in the presence of recombination (Wilson and McVean 2006) (Fig. 4). The genome-wide estimates of dN/dS ratio (or ω) indicate a generally strongly negative selective pressure with average values ranging from 0.015 to 0.035 in the non-human hosts and 0.055 to 0.067 in the human host (grey horizontal bars in Fig. 4 with a Y-axis on a log-scale). The bovine data set was the only non-human data set for which the site-specific estimates provide evidence for two sites with an ω value significantly larger than 1. In contrast, a non-negligible number of positively selected sites (ranging from 20 to 25) was consistently identified in the HCV data sets, primarily located in the antigenically important E1/E2 gene region.

Because hepacivirus evolutionary rates have only been estimated for HCV, we here explore how informative current sampling in other host lineages is about the tempo of hepacivirus evolution while accounting for recombination (cfr. Section 2). Using a recently developed test that compares the fit of a model that incorporates sampling time (the ‘dated tip’ model) to a model that assumes sampling time is uninformative (all sequences are sampled contemporaneously) (Duchene et al. 2019), we provide formal evidence that there is insufficient temporal signal in bovine, equine and the two rodent lineages tested (Table 1). In the different HCV data sets, on the other hand, temporal signal is consistently supported by a log Bayes factor support >3. This discrepancy is likely explained by the difference in sampling time intervals and how uniformly genomes can be sampled over this interval. Although the equine lineage has the broadest sampling time range, it is highly unbalanced with three closely related donkey viruses sampled in 1979 and all other viruses sampled between 2011 and 2016 (Supplementary Fig. S6).

**Table 1.** Bayesian evaluation of temporal signal in hepaciviruses.

Lineage	No. of dated sequences	Sampling time range	BETS ln Bayes factor
			(dated vs contemporaneous)
Bovine	14	2013–2017	0.26
Equine	22	1979–2016	−5.6
Rodent I	11	2010–2013	0.1
Rodent III	36	2010–2013	−0.28
HCV1a	35	1997–2014	6.19
HCV1b	34	1990–2015	37.08
HCV3a	35	2002–2014	5.11

## 4. Discussion

In this study, we performed a large-scale screening for hepaciviruses in African small mammals with a strong focus on rodents. We detect hepaciviruses in twenty-nine animal species that had not been screened before, and therefore, considerably expand the RHV host spectrum. In line with previous research (Drexler et al. 2013; Kapoor et al. 2013; Van Nguyen et al. 2018), we demonstrate that rodents constitute an important source of hepaciviruses and that the evolutionary history of those pathogens has been largely shaped by host switching events. Finally, we identify a high number of hepacivirus co-infections among *Lophuromys* rodents and conduct comparative evolutionary analyses for specific host lineages.

While bats have received much attention as important pathogen reservoirs of infectious diseases, equally large-scale surveillance efforts have focused on rodents and, to a lesser extent, other small mammals. Rodents are generally considered as major transmitters of zoonoses and carry more than sixty-six pathogens that have crossed species barriers and infected humans (Woolhouse and Gaunt 2007; Han et al. 2015). The number of virus lineages carried by vertebrate orders appears to be mainly correlated with the number of species present in these orders (Mollentze and Streicker 2020). Therefore, species-rich orders such as bats and rodents can be expected to host a higher number of viruses with zoonotic potential (Mollentze and Streicker 2020).

In our screening, 1.86 per cent of the samples were positive for HCV homologues, a prevalence consistent with previous rodent screening efforts performed by Drexler et al. (2013). That study detected hepaciviruses in 1.8 per cent of the *Myodes glareolus* population tested and a prevalence of 1.9 per cent in *Rhabdomys pumilio* species. For RHV detection, we adopted a PCR-based screening assay with primers targeting a conserved region of the hepacivirus genome (Kapoor et al. 2013). Although a targeted approach may not be able to detect all strains in highly diverse populations, we were able to capture divergent viruses in many different rodent hosts and one bat species. Directly applying metagenomic sequencing without prior PCR screening may avoid detection biases and would also allow identifying other pathogens. However, given the relatively low prevalence of hepaciviruses within various hosts, a PCR approach provides an efficient and cost-effective method for large-scale screening of hepaciviruses.

A hepacivirus nomenclature consisting of fourteen species (*Hepacivirus A–N*, Smith et al. 2016) has been proposed based on the amino acid divergence in distinct parts of the hepacivirus polyprotein. As more information accumulates on the genetic diversity of those pathogens, it becomes extremely challenging to define specific criteria for their classification (Simmonds et al. 2017). The current demarcation criteria do not adequately accommodate the high genetic diversity of hepaciviruses because they lead to discrepancies in the number of assigned species, as is demonstrated in our analysis (Supplementary Fig. S4). This calls into question the current demarcation criteria and leaves hepacivirus classification as an open issue for further discussion.

Despite many host switches, there is still some non-random clustering of hepaciviruses according to rodent taxa. All *Lophuromys* hepacivirus clades form monophyletic groups exclusive to *Lophuromys* species, despite the fact that they have been sampled thousands of kilometers apart. Furthermore, hepaciviruses sampled from other rodent taxa that are geographically much closer to some of the brush furred rats we sampled belong to different hepaci-lineages. This indicates that the hepacivirus evolutionary history has, at least to some extent, been driven by confinement to specific rodent taxa. These observations fit to some extent with an ancient evolutionary history constrained by host boundaries.

Characterizing hepaciviruses in rodents may also prove relevant for HCV vaccine research. While treatment with direct-acting antiviral compounds is now highly effective, a prophylactic vaccine is still lacking due to the absence of an *in vivo* model to study virus–host interactions in the liver. This has been an active field of research that made considerable progress in the development of surrogate rat models of chronic HCV infection (Billerbeck et al. 2017; Hartlage et al. 2019). Our work may motivate further biological characterization of RHVs, and the evidence of hepacivirus co-infections in specific rodents may have interesting immunological implications.

We only observed co-infections in brush furred rats, even though various rodent genera have been found to carry hepaciviruses. To date, relatively little is known about the behavioural ecology of the four closely related *Lophuromys* species that harboured co-infections. These belong to the so-called *L. flavopunctatus* complex (Verheyen et al. 2002, 2007) and they diverged in Pleistocene in different forest fragments (Onditi et al. 2021). Most of the species from this complex are endemic in central and east Africa (Sabuni et al. 2018; Van de Perre et al. 2019a). Although *Lophuromys* tend to be solitary and show antagonist behaviour to conspecifics, they can sometimes live in high population densities. In captivity they may fight until death and if such conflicts occur in natural circumstances, it may represent a mode of transmission that could help to explain the elevated number of co-infections. Furthermore, these rodents can be occasionally infested with blood-sucking fleas depending on the location and the specific flea index. Flea sharing between sympatric species of rodents has been previously described (Laudisoit et al. 2009) and could possibly support a scenario of RHV mechanical transmission.

Interestingly, a co-infection of two divergent paramyxovirus lineages has also been found exclusively in a *Lophuromys* specimen (Vanmechelen et al. 2018). Whether the apparent propensity of brush furred rats to be co-infected with multiple lineages of the same virus family is due to a common physiological background of the closely related species that enhances their susceptibility or tolerance of multiple hepacivirus/paramyxovirus infections, or because of behavioural characteristics that increases the transmission probabilities, is still unknown. Further research is needed into the heterogeneous viral detection and co-infection rate in rodents and how those are shaped by specific transmission dynamics.

As part of our evolutionary analyses, we focused on recombination within specific host lineages as an important driver of genetic diversity. Recombination is relatively uncommon in the extensively studied HCV population (González-Candelas et al. 2011; Raghwani et al. 2012; Karchava et al. 2015; Susser et al. 2017) and while some evidence for interspecific hepacivirus recombination has been found (Thézé et al. 2015), the authors indicated that a clear interpretation of this result is hampered by high genetic divergence and undersampling. We detected significant signal in the bovine, equine and two rodent lineages, which implies that co-infections also occur in other animal hosts.

Using selection analyses that account for recombination, we estimate an overall negative selection pressure on the virus population in each host providing evidence for a process of evolution under predominantly purifying selection. However, this does not exclude the possibility of episodic molecular adaptation in the evolutionary history of these viruses, for example, following a cross-species transmission to a new host. Unfortunately, the extensive interspecific genetic divergence hampers uncovering such events in codon sequences. We consistently identify a similar fraction of positively selected sites in three HCV genotype data sets, in particular in the E1/E2 region, while such sites are rare or absent in hepaciviruses in animal hosts. It is therefore interesting to speculate that differences in immune responses may, together with differences in transmission intensity, underly some variability in hepacivirus co-infections and hence also differences in recombination rates. However, also treatment could to some extent explain the larger number of positively sites in HCV.

For rapidly evolving RNA viruses, evolutionary rates can be estimated based on the sequence divergence that accumulates between genome samples obtained at different time points. We demonstrate that the current sampling time range is insufficient for calibrating a hepacivirus molecular clock in the different animal hosts. This calls for further characterization of hepacivirus genomes, from both old samples and more recent samples, in order to capture sufficient temporal signal. This will provide the ability to estimate divergence times in the hepacivirus evolutionary history as well as to study the biological factors underlying evolutionary rate variation.

In conclusion, we show that viral genomic studies provide important information about the diversity, transmission history within and among different hosts, and evolutionary dynamics of hepaciviruses. We hope that screening efforts guided by ecologists will target not only wild animals but also commensal species that live in close proximity to residential areas. Characterizing possible routes of transmission among those hosts and/or between different hosts may prove particularly interesting as it may provide insights into the ecological barriers for viruses at the rodent–human interface. Hopefully, the expanding hepacivirus diversity will motivate further biological studies aimed at elucidating hepacivirus transmission routes and modes of infection.

## Supplementary data

Supplementary data are available at *Virus Evolution* online.

## Supplementary Material

veab036_Supplementary_Data

## References

[veab036-B1] Baechlein C. et al (2015) ‘Identification of a Novel Hepacivirus in Domestic Cattle from Germany’, Journal of Virology, 89: 7007–15. 25926652 10.1128/JVI.00534-15 PMC4473572

[veab036-B2] Bankevich A. et al (2012) ‘Spades: A New Genome Assembly Algorithm and Its Applications to Single-Cell Sequencing’, Journal of Computational Biology, 19: 455–77. 22506599 10.1089/cmb.2012.0021 PMC3342519

[veab036-B3] Billerbeck E. et al (2017) ‘Mouse Models of Acute and Chronic Hepacivirus Infection’, Science, 357: 204–8. 28706073 10.1126/science.aal1962 PMC5654634

[veab036-B4] Biomatters (2019) *Geneious Prime* <https://www.geneious.com> last accessed 18 March 2021.

[veab036-B5] Bletsa M. et al (2019) High-quality RNA purification with on-column DNAse treatment from tissue specimens v1 (protocols.io.8ufhwtn). *protocols.io.*

[veab036-B6] Bolger A. M. , Lohse M., Usadel B. (2014) ‘Trimmomatic: A Flexible Trimmer for Illumina Sequence Data’, Bioinformatics, 30: 2114–20. 24695404 10.1093/bioinformatics/btu170 PMC4103590

[veab036-B7] Bruen T. C. , Philippe H., Bryant D. (2006) ‘A Simple and Robust Statistical Test for Detecting the Presence of Recombination’, Genetics, 172: 2665–81. 16489234 10.1534/genetics.105.048975 PMC1456386

[veab036-B8] Bryja J. et al (2012) ‘Revised Occurrence of Rodents from the Tribe Praomyini (Muridae) in zambia Based on Mitochondrial DNA Analyses: Implications for Biogeography and Conservation’, Folia Zoologica, 61: 268–83.

[veab036-B9] Bryja J. et al (2014) ‘The Role of Dispersal and Vicariance in the p Leistocene History of an e Ast a Frican Mountain Rodent, p Raomys Delectorum’, Journal of Biogeography, 41: 196–208.

[veab036-B10] Burbelo P. D. et al (2012) ‘Serology-Enabled Discovery of Genetically Diverse Hepaciviruses in a New Host’, Journal of Virology, 86: 6171–8. 22491452 10.1128/JVI.00250-12 PMC3372197

[veab036-B11] Canuti M. et al (2019) ‘Virus Discovery Reveals Frequent Infection by Diverse Novel Members of the Flaviviridae in Wild Lemurs’, Archives of Virology, 164: 509–22. 30460488 10.1007/s00705-018-4099-9

[veab036-B12] Chang W.-S. et al (2019) ‘Metagenomic Discovery and co-Infection of Diverse Wobbly Possum Disease Viruses and a Novel Hepacivirus in Australian Brushtail Possums’, One Health Outlook, 1: 5. 33829126 10.1186/s42522-019-0006-x PMC7990097

[veab036-B13] Chernomor O. et al (2015) ‘Split Diversity in Constrained Conservation Prioritization Using Integer Linear Programming’, Methods in Ecology and Evolution, 6: 83–91. 25893087 10.1111/2041-210X.12299 PMC4392707

[veab036-B14] Choo Q.-L. et al (1989) ‘Isolation of a Cdna Clone Derived from a Blood-Borne Non-a, Non-b Viral Hepatitis Genome’, Science, 244: 359–62. 2523562 10.1126/science.2523562

[veab036-B15] Chu L. et al (2019) ‘A Highly Divergent Hepacivirus-like Flavivirus in Domestic Ducks’, Journal of General Virology, 100: 1234–40. 10.1099/jgv.0.001298 31282853

[veab036-B16] Corman V. M. et al (2015) ‘Highly Divergent Hepaciviruses from African Cattle’, Journal of Virology, 89: 5876–82. 25787289 10.1128/JVI.00393-15 PMC4442428

[veab036-B17] Criscuolo A. , Gribaldo S. (2010) ‘BMGE (Block Mapping and Gathering with Entropy): A New Software for Selection of Phylogenetic Informative Regions from Multiple Sequence Alignments’, BMC Evolutionary Biology, 10: 210. 20626897 10.1186/1471-2148-10-210 PMC3017758

[veab036-B18] Drexler J. F. et al (2013) ‘Evidence for Novel Hepaciviruses in Rodents’, PLoS Pathogens, 9: e1003438. 23818848 10.1371/journal.ppat.1003438 PMC3688547

[veab036-B19] Duchene S. et al (2019) ‘Bayesian Evaluation of Temporal Signal in Measurably Evolving Populations’, bioRxiv, 810697. 10.1093/molbev/msaa163 PMC7454806 32895707

[veab036-B20] Edgar R. C. (2004) ‘Muscle: Multiple Sequence Alignment with High Accuracy and High Throughput’, Nucleic Acids Research, 32: 1792–7. 15034147 10.1093/nar/gkh340 PMC390337

[veab036-B21] El-Attar L. et al (2015) ‘Detection of Non-Primate Hepaciviruses in uk Dogs’, Virology, 484: 93–102. 26086431 10.1016/j.virol.2015.05.005 PMC7111718

[veab036-B22] Firth C. et al (2014) ‘Detection of Zoonotic Pathogens and Characterization of Novel Viruses Carried by Commensal rattus norvegicus in New York City’, mBio, 5: e01933–14. 25316698 10.1128/mBio.01933-14 PMC4205793

[veab036-B23] Gaudieri S. et al (2006) ‘Evidence of Viral Adaptation to Hla Class i-Restricted Immune Pressure in Chronic Hepatitis c Virus Infection’, Journal of Virology, 80: 11094–104. 17071929 10.1128/JVI.00912-06 PMC1642167

[veab036-B24] Goldberg T. L. et al (2019) ‘Multidecade Mortality and a Homolog of Hepatitis C Virus in Bald Eagles (*Haliaeetus leucocephalus*), the National Bird of the USA’, Scientific Reports, 9: 14953. 31628350 10.1038/s41598-019-50580-8 PMC6802099

[veab036-B25] González-Candelas F. , López-Labrador F. X., Bracho M. A. (2011) ‘Recombination in Hepatitis c Virus’, Viruses, 3: 2006–24. 22069526 10.3390/v3102006 PMC3205392

[veab036-B26] Gouy M. , Guindon S., Gascuel O. (2010) ‘Seaview Version 4: A Multiplatform Graphical User Interface for Sequence Alignment and Phylogenetic Tree Building’, Molecular Biology and Evolution, 27: 221–4. 19854763 10.1093/molbev/msp259

[veab036-B27] Goüy de Bellocq J. et al (2010) ‘Sympatric Occurrence of 3 Arenaviruses, Tanzania’, Emerging Infectious Diseases, 16: 692–5. 20350390 10.3201/eid1604.091721 PMC3321973

[veab036-B28] Gray R. R. et al (2011) ‘The Mode and Tempo of Hepatitis c Virus Evolution within and among Hosts’, BMC Evolutionary Biology, 11: 1–10. 21595904 10.1186/1471-2148-11-131 PMC3112090

[veab036-B29] Gryseels S. et al (2015) ‘Gairo Virus, a Novel Arenavirus of the Widespread Mastomys Natalensis: Genetically Divergent, but Ecologically Similar to Lassa and Morogoro Viruses’, Virology, 476: 249–56. 25559385 10.1016/j.virol.2014.12.011

[veab036-B30] Gryseels S. et al (2017) ‘When Viruses Don’t Go Viral: The Importance of Host Phylogeographic Structure in the Spatial Spread of Arenaviruses’, PLoS Pathogens, 13: e1006073. 28076397 10.1371/journal.ppat.1006073 PMC5226678

[veab036-B31] Guo H. et al (2019) ‘Novel Hepacivirus in Asian House Shrew, China’, Science China Life Sciences, 62: 701–4. 30701456 10.1007/s11427-018-9435-7 PMC7088713

[veab036-B32] Han B. A. et al (2015) ‘Rodent Reservoirs of Future Zoonotic Diseases’, Proceedings of the National Academy of Sciences, 112: 7039–44. 10.1073/pnas.1501598112 PMC4460448 26038558

[veab036-B33] Hartlage A. S. , Cullen J. M., Kapoor A. (2016) ‘The Strange, Expanding World of Animal Hepaciviruses’, Annual Review of Virology, 3: 53–75. 10.1146/annurev-virology-100114-055104 PMC5523456 27741408

[veab036-B34] Hartlage A. S. et al (2019) ‘Vaccination to Prevent t Cell Subversion Can Protect against Persistent Hepacivirus Infection’, Nature Communications, 10: 1–11. 10.1038/s41467-019-09105-0 PMC6405742 30846697

[veab036-B35] Harvey E. et al (2018) ‘Extensive Diversity of Rna Viruses in Australian Ticks’, Journal of Virology, 93: e01358–18. 10.1128/JVI.01358-18 PMC6340049 30404810

[veab036-B36] Jombart T. , Balloux F., Dray S. (2010) ‘Adephylo: New Tools for Investigating the Phylogenetic Signal in Biological Traits’, Bioinformatics, 26: 1907–9. 20525823 10.1093/bioinformatics/btq292

[veab036-B37] Kapoor A. et al (2011) ‘Characterization of a Canine Homolog of Hepatitis c Virus’, Proceedings of the National Academy of Sciences, 108: 11608–13. 10.1073/pnas.1101794108 PMC3136326 21610165

[veab036-B38] Kapoor A. et al (2013) ‘Identification of Rodent Homologs of Hepatitis c Virus and Pegiviruses’, mBio, 4: e00216–13. 23572554 10.1128/mBio.00216-13 PMC3622934

[veab036-B39] Karchava M. et al (2015) ‘High Incidence of the Hepatitis c Virus Recombinant 2k/1b in georgia: Recommendations for Testing and Treatment’, Hepatology Research, 45: 1292–8. 25689487 10.1111/hepr.12505 PMC4787595

[veab036-B40] Katoh K. , Asimenos G., Toh H. (2009) ‘Multiple Alignment of DNA Sequences with MAFFT’, Methods Mol Biol, 537: 39–64. 19378139 10.1007/978-1-59745-251-9_3

[veab036-B41] Kumar S. , Stecher G., Tamura K. (2016) ‘Mega7: Molecular Evolutionary Genetics Analysis Version 7.0 for Bigger Datasets’, Molecular Biology and Evolution, 33: 1870–4. 27004904 10.1093/molbev/msw054 PMC8210823

[veab036-B42] Lam H. M. , Ratmann O., Boni M. F. (2018) ‘Improved Algorithmic Complexity for the 3seq Recombination Detection Algorithm’, Molecular Biology and Evolution, 35: 247–51. 29029186 10.1093/molbev/msx263 PMC5850291

[veab036-B43] Langmead B. , Salzberg S. L. (2012) ‘Fast Gapped-Read Alignment with Bowtie 2’, Nature Methods, 9: 357–9. 22388286 10.1038/nmeth.1923 PMC3322381

[veab036-B44] Larsson A. (2014) ‘Aliview: A Fast and Lightweight Alignment Viewer and Editor for Large Datasets’, Bioinformatics, 30: 3276–8. 25095880 10.1093/bioinformatics/btu531 PMC4221126

[veab036-B45] Lauck M. et al (2013) ‘A Novel Hepacivirus with an Unusually Long and Intrinsically Disordered ns5a Protein in a Wild Old World Primate’, Journal of Virology, 87: 8971–81. 23740998 10.1128/JVI.00888-13 PMC3754081

[veab036-B46] Laudisoit A. et al (2009) ‘Seasonal and Habitat Dependence of Fleas Parasitic on Small Mammals in Tanzania’, Integrative Zoology, 4: 196–212. 21392290 10.1111/j.1749-4877.2009.00150.x

[veab036-B47] Lecompte E. et al (2008) ‘Phylogeny and Biogeography of African Murinae Based on Mitochondrial and Nuclear Gene Sequences, with a New Tribal Classification of the Subfamily’, BMC Evolutionary Biology, 8: 199. 18616808 10.1186/1471-2148-8-199 PMC2490707

[veab036-B48] Lole K. S. et al (1999) ‘Full-Length Human Immunodeficiency Virus Type 1 Genomes from Subtype c-Infected Seroconverters in india, with Evidence of Intersubtype Recombination’, Journal of Virology, 73: 152–60. 9847317 10.1128/jvi.73.1.152-160.1999 PMC103818

[veab036-B49] Lyons S. et al (2012) ‘Nonprimate Hepaciviruses in Domestic Horses’, Emerging Infectious Diseases, 18: 1976–82. 23171728 10.3201/eid1812.120498 PMC3557883

[veab036-B50] Makundi R. H. et al (2015) ‘We Are Connected: Flea–Host Association Networks in the Plague Outbreak Focus in the Rift Valley, Northern Tanzania’, Wildlife Research, 42: 196.

[veab036-B51] Martin D. , Rybicki E. (2000) ‘Rdp: Detection of Recombination Amongst Aligned Sequences’, Bioinformatics, 16: 562–3. 10980155 10.1093/bioinformatics/16.6.562

[veab036-B52] Martin D. et al (2005) ‘A Modified Bootscan Algorithm for Automated Identification of Recombinant Sequences and Recombination Breakpoints’, AIDS Research and Human Retroviruses, 21: 98–102. 15665649 10.1089/aid.2005.21.98

[veab036-B53] Martin D. P. et al (2015) ‘Rdp4: Detection and Analysis of Recombination Patterns in Virus Genomes’, Virus Evol, 1: vev003. 27774277 10.1093/ve/vev003 PMC5014473

[veab036-B54] Massawe A. W. et al (2012) ‘Breeding Dynamics of Rodent Species Inhabiting Farm–Fallow Mosaic Fields in Central Tanzania’, African Zoology, 47: 128–37.

[veab036-B55] Mazoch V. et al (2018) ‘Phylogeography of a Widespread Sub-Saharan Murid Rodent Aethomys Chrysophilus: The Role of Geographic Barriers and Paleoclimate in the Zambezian Bioregion’, Mammalia, 82: 373–87.

[veab036-B56] Meheretu Y. et al (2012) ‘High Diversity of Rna Viruses in Rodents, Ethiopia’, Emerging Infectious Diseases, 18: 2047–50. 23171649 10.3201/eid1812.120596 PMC3557881

[veab036-B57] Messina J. P. et al (2015) ‘Global Distribution and Prevalence of Hepatitis c Virus Genotypes’, Hepatology, 61: 77–87. 25069599 10.1002/hep.27259 PMC4303918

[veab036-B58] Mollentze N. , Streicker D. G. (2020) ‘Viral Zoonotic Risk is Homogenous among Taxonomic Orders of Mammalian and Avian Reservoir Hosts’, Proceedings of the National Academy of Sciences, 117: 9423–30. 10.1073/pnas.1919176117 PMC7196766 32284401

[veab036-B59] Moreira-Soto A. et al (2020) ‘A Novel Sloth Hepacivirus Corroborates Cross-Order Host Switches during the Genealogy of the Genus Hepacivirus’, Virus Evolution, 6:10.1093/ve/veaa033 PMC7368370 32704383

[veab036-B60] Nguyen L.-T. et al (2015) ‘Iq-Tree: A Fast and Effective Stochastic Algorithm for Estimating Maximum-Likelihood Phylogenies’, Molecular Biology and Evolution, 32: 268–74. 25371430 10.1093/molbev/msu300 PMC4271533

[veab036-B61] Onditi K. O. et al (2021) ‘Systematics and Phylogeography of the Non-Ethiopian Speckled-Pelage Brush-Furred Rats (Lophuromys Flavopunctatus Group) Inferred from Integrative Genetics and Morphometry’, BMC Ecology and Evolution. 10.1186/s12862-021-01813-w PMC8132446 34011264

[veab036-B62] Paradis E. , Schliep K. (2019) ‘Ape 5.0: An Environment for Modern Phylogenetics and Evolutionary Analyses in R’, Bioinformatics, 35: 526–8. 30016406 10.1093/bioinformatics/bty633

[veab036-B63] Petružela J. et al (2018) ‘Spiny Mice of the Zambezian Bioregion–Phylogeny, Biogeography and Ecological Differentiation within the Acomys Spinosissimus Complex’, Mammalian Biology, 91: 79–90.

[veab036-B64] Porter A. F. et al (2020) ‘Novel Hepaci-and Pegi-like Viruses in Native Australian Wildlife and Non-Human Primates’, Virus Evolution, 6: veaa064. 10.1093/ve/veaa064 PMC7673076 33240526

[veab036-B65] Pybus O. G. , Gray R. R. (2013) ‘Virology: The Virus Whose Family Expanded’, Nature, 498: 310–1. 23783626 10.1038/498310b PMC7095075

[veab036-B66] Pybus O. G. , Thézé J. (2016) ‘Hepacivirus Cross-Species Transmission and the Origins of the Hepatitis c Virus’, Current Opinion in Virology, 16: 1–7. 26517843 10.1016/j.coviro.2015.10.002

[veab036-B67] Quan P.-L. et al (2013) ‘Bats Are a Major Natural Reservoir for Hepaciviruses and Pegiviruses’, Proceedings of the National Academy of Sciences, 110: 8194–9. 10.1073/pnas.1303037110 PMC3657805 23610427

[veab036-B68] Raghwani J. et al (2012) ‘Origin and Evolution of the Unique Hepatitis C Virus Circulating Recombinant Form 2k/1b’, Journal of Virology, 86: 2212–20. 22114341 10.1128/JVI.06184-11 PMC3302385

[veab036-B69] Rambaut A. et al (2016) ‘Exploring the Temporal Structure of Heterochronous Sequences Using Tempest (Formerly Path-o-Gen)’, Virus Evolution, 2: vew007. 27774300 10.1093/ve/vew007 PMC4989882

[veab036-B70] Sabuni C. et al (2018) ‘Biogeographic Implications of Small Mammals from Northern Highlands in Tanzania with First Data from the Volcanic Mount Kitumbeine’, Mammalia, 82: 360–72.

[veab036-B71] Salminen M. O. et al (1995) ‘Identification of Breakpoints in Intergenotypic Recombinants of Hiv Type 1 by Bootscanning’, AIDS Research and Human Retroviruses, 11: 1423–5. 8573403 10.1089/aid.1995.11.1423

[veab036-B72] Schmieder R. , Edwards R. (2011) ‘Quality Control and Preprocessing of Metagenomic Datasets’, Bioinformatics, 27: 863–4. 21278185 10.1093/bioinformatics/btr026 PMC3051327

[veab036-B73] Shi M. et al (2018) ‘The Evolutionary History of Vertebrate Rna Viruses’, Nature, 556: 197–202. 29618816 10.1038/s41586-018-0012-7

[veab036-B74] Simmonds P. et al (2017) ‘Consensus Statement: Virus Taxonomy in the Age of Metagenomics’, Nature Reviews Microbiology, 15: 161–8. 28134265 10.1038/nrmicro.2016.177

[veab036-B75] Smith D. B. et al (2016) ‘Proposed Update to the Taxonomy of the Genera Hepacivirus and Pegivirus within the Flaviviridae Family’, Journal of General Virology, 97: 2894–907. 10.1099/jgv.0.000612 PMC5770844 27692039

[veab036-B76] Susser S. et al (2017) ‘Origin, Prevalence and Response to Therapy of Hepatitis c Virus Genotype 2k/1b Chimeras’, Journal of Hepatology, 67: 680–6. 28619439 10.1016/j.jhep.2017.05.027

[veab036-B77] Těšíková J. et al (2017) ‘Hantavirus Strains in East Africa Related to Western African Hantaviruses’, Vector-Borne and Zoonotic Diseases, 17: 278–80. 28075239 10.1089/vbz.2016.2022

[veab036-B78] Thézé J. et al (2015) ‘Evolutionary and Phylogenetic Analysis of the Hepaciviruses and Pegiviruses’, Genome Biology and Evolution, 7: 2996–3008. 26494702 10.1093/gbe/evv202 PMC5635594

[veab036-B79] Van de Perre F. et al (2018) ‘Reconciling Biodiversity and Carbon Stock Conservation in an Afrotropical Forest Landscape’, Science Advances, 4: eaar6603. 29670947 10.1126/sciadv.aar6603 PMC5903881

[veab036-B80] Van de Perre F. et al (2019a) *African Mammalia* <http://projects.biodiversity.be/africanmammalia> last accessed on 16 January 2020.

[veab036-B81] Van de Perre F. et al (2019b) ‘Shrews (Soricidae) of the Lowland Forests around Kisangani (DR Congo)’, Biodivers Data Journal, 7: e46948. 10.3897/BDJ.7.e46948 PMC6934628 31885462

[veab036-B82] Van Nguyen D. et al (2018) ‘Detection and Characterization of Homologues of Human Hepatitis Viruses and Pegiviruses in Rodents and Bats in Vietnam’, Viruses, 10: 102. 10.3390/v10030102 PMC5869495 29495551

[veab036-B83] Vanmechelen B. et al (2018) ‘Discovery and Genome Characterization of Three New Jeilongviruses, a Lineage of Paramyxoviruses Characterized by Their Unique Membrane Proteins’, BMC Genomics, 19: 617. 30115009 10.1186/s12864-018-4995-0 PMC6097224

[veab036-B84] Verheyen W. et al (2002) ‘The Lophuromys Flavopunctatus thomas1888 sl Species Complex: A Craniometric Study, with the Description and Genetic Characterization of Two New Species (Rodentia-Muridae-Africa). *Bulletin de L’Institut Royal Des*’, Sciences Naturelles de Belgique Biologie, 72: 141–82.

[veab036-B85] Verheyen W. N. et al (2007) ‘The Characterization of the Kilimanjaro Lophuromys Aquilus True 1892 Population and the Description of Five New Lophuromys Species (Rodentia, Muridae)’, Koninklijk Belgisch Instituut Voor Natuurwetenschappen. Studiedocumenten, 77: 23–75.

[veab036-B86] Walter S. et al (2017) ‘Differential Infection Patterns and Recent Evolutionary Origins of Equine Hepaciviruses in Donkeys’, Journal of Virology, 91: e01711–16. 27795428 10.1128/JVI.01711-16 PMC5165184

[veab036-B87] Wang L.-G. et al (2019) ‘Treeio: An R Package for Phylogenetic Tree Input and Output with Richly Annotated and Associated Data’, Molecular Biology and Evolution. 10.1093/molbev/msz240 PMC6993851 31633786

[veab036-B88] Watson S. J. et al (2013) ‘Viral Population Analysis and Minority-Variant Detection Using Short Read Next-Generation Sequencing’, Philosophical Transactions of the Royal Society B: Biological Sciences, 368: 20120205. 10.1098/rstb.2012.0205 PMC3678329 23382427

[veab036-B89] Williams S. H. et al (2020) ‘Discovery of Jogalong Virus, a Novel Hepacivirus Identified in a Culex Annulirostris (Skuse) Mosquito from the Kimberley Region of Western Australia’, PloS One, 15: e0227114. 31899786 10.1371/journal.pone.0227114 PMC6941808

[veab036-B90] Wilson D. J. , McVean G. (2006) ‘Estimating Diversifying Selection and Functional Constraint in the Presence of Recombination’, Genetics, 172: 1411–25. 16387887 10.1534/genetics.105.044917 PMC1456295

[veab036-B91] Woolhouse M. , Gaunt E. (2007) ‘Ecological Origins of Novel Human Pathogens’, Critical Reviews in Microbiology, 33: 231–42. 18033594 10.1080/10408410701647560

[veab036-B92] Yu G. et al (2018) ‘Two Methods for Mapping and Visualizing Associated Data on Phylogeny Using Ggtree’, Molecular Biology and Evolution, 35: 3041–3. 30351396 10.1093/molbev/msy194 PMC6278858

[veab036-B93] Zaharia M. et al (2011) ‘Faster and More Accurate Sequence Alignment with Snap’, arXiv, 1111–5572.

