# Supplementary material for: Molecular detection and genomic characterization of diverse hepaciviruses in African rodents
Source: Virus Evol. 2021 Apr 12;7(1):veab036. doi: 10.1093/ve/veab036 (PMC8242229; doi:10.1093/ve/veab036)
Supplement: veab036_Supplementary_Data [file veab036_supplementary_data.zip › hepaci_SI_VE.pdf]

Supplementary information for 'Molecular detection and genomic characterisation of diverse hepaciviruses in African rodents'

Magda Bletsa[1] #a, Bram Vrancken[1], Sophie Gryseels[1,2], Ine Boonen[1], Antonios Fikatas[1], Yiqiao Li[1], Anne Laudisoit[3], Sebastian Lequime[1], Josef Bryja[4], Rhodes Makundi[5], Yonas Meheretu[6], Benjamin Dudu Akaibe[7], Sylvestre Gambalemoke Mbalitini[7], Frederik Van de Perre[2], Natalie Van Houtte[2], Jana Těšíková[4,8], Elke Wollants[1], Marc Van Ranst[1], Oliver G. Pybus[9,10], Jan Felix Drexler[11,12], Erik Verheyen[2,13], Herwig Leirs[2], Joelle Gouy de Bellocq[4] and Philippe Lemey[1] #b*

[1]*Department of Microbiology, Immunology and Transplantation, Rega Institute, KU Leuven, Leuven, Belgium*

[2]*Department of Biology, Evolutionary Ecology Group, University of Antwerp, Antwerp, Belgium*

[3]*EcoHealth Alliance, New York, USA*

[4]*Institute of Vertebrate Biology of the Czech Academy of Sciences, Brno, Czech Republic*

[5]*Pest Management Center – Sokoine University of Agriculture, Morogoro, Tanzania*

[6]*Department of Biology and Institute of Mountain Research & Development, Mekelle University, Mekelle, Ethiopia*

[7]*Department of Ecology and Animal Resource Management, Faculty of Science, Biodiversity Monitoring Center, University of Kisangani, Kisangani, Democratic Republic of the Congo*

[8]*Department of Botany and Zoology, Faculty of Science, Masaryk University, Brno, Czech Republic*

[9]*Department of Zoology, University of Oxford, Oxford, UK*

[10]*Department of Pathobiology and Population Sciences, The Royal Veterinary College, London, UK*

[11]*Charite - Universitatsmedizin Berlin, Berlin, Germany*

[12]*German Center for Infection Research (DZIF), Germany and*

[13]*OD Taxonomy and Phylogeny - Royal Belgian Institute of Natural Sciences, Brussels,*

Belgium

*Electronic address: magda.bletsa@kuleuven.be; Electronic address: philippe.lemey@kuleuven.be

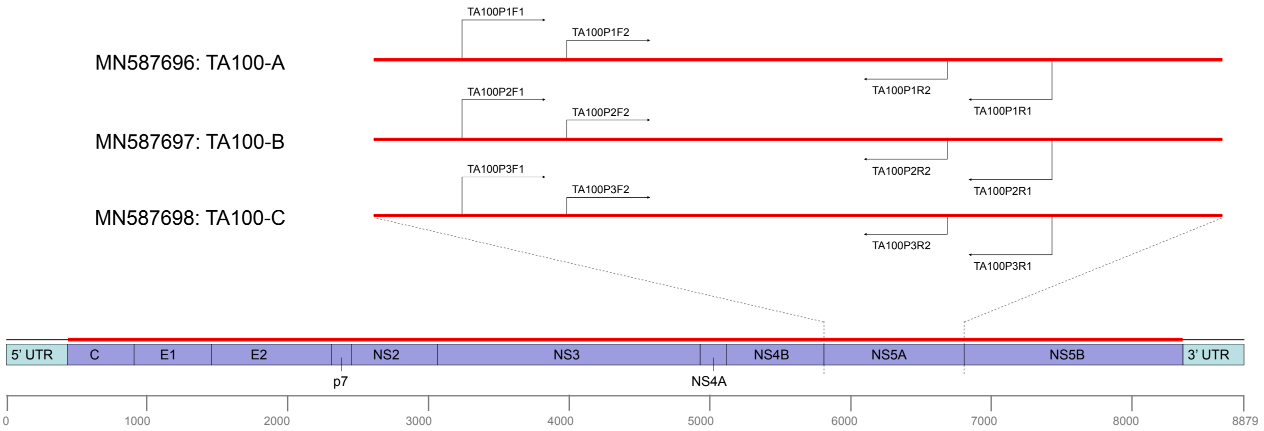

FIG. 1 **Schematic overview of the primers used in our co-infection validation assay.** Purple and light blue boxes correspond to the full genome organisation of rodent hepaciviruses. Numbering of positions is relative to GenBank accession number NC_021153. Primers P1, P2 and P3 denote the distinct PCR assays designed on the three divergent hepaciviruses of specimen TA100. F1 and R1 represent the outer forward and reverse primers, respectively. Accordingly, F2 and R2 refer to the inner forward and reverse primers. For sample TA100 for example, we will have primer pair TA100P1F1 – TA100P1R1, which represents the outer primer pair designed specifically to amplify a fragment of the NS5A region of TA100-A (accession number: MN587696).

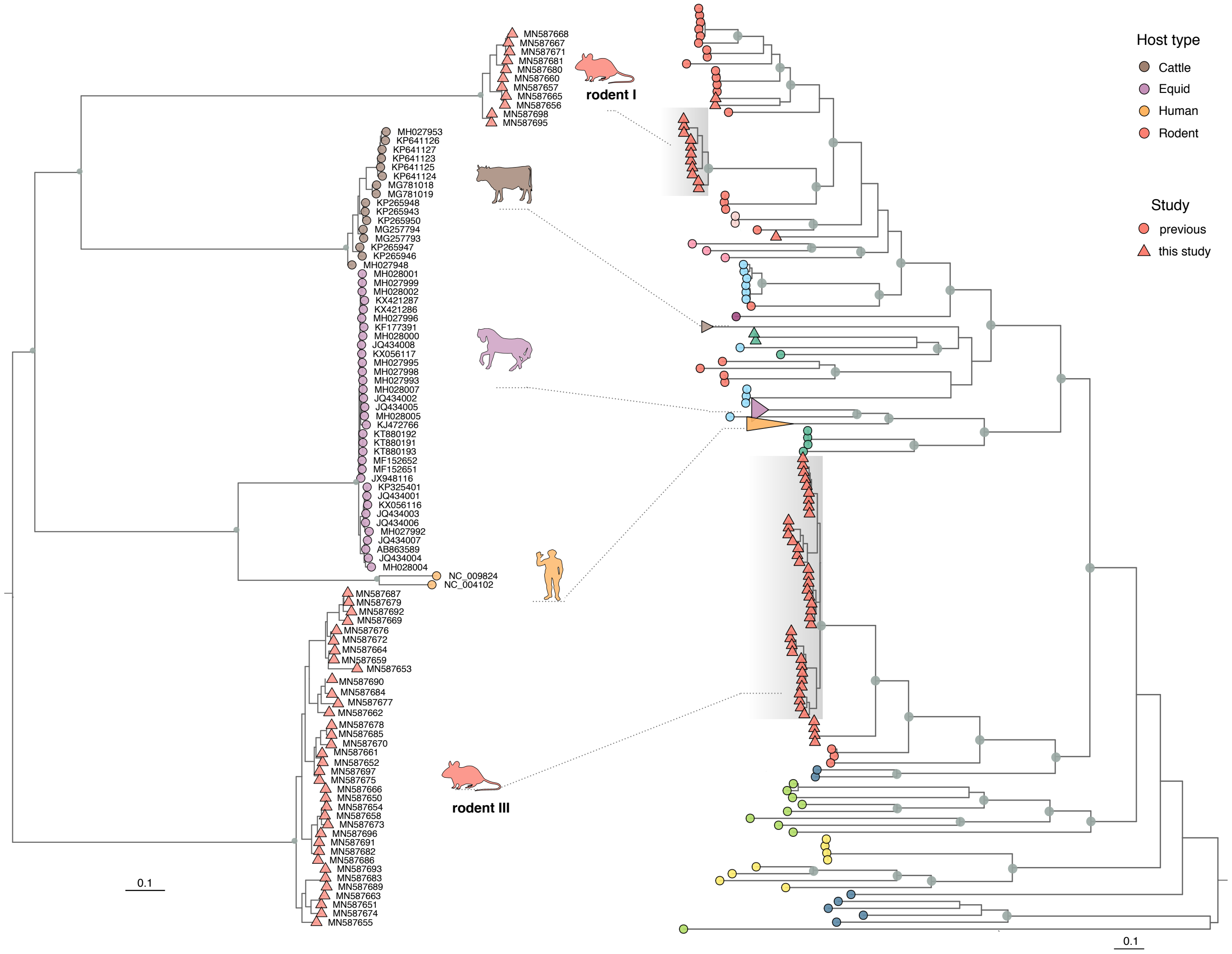

FIG. 2 **Host-specific lineages used in the recombination and selection analyses.** ML subtree of the genome-wide phylogenetic reconstruction depicted in main Figure 2. Five host-specific lineages were analysed (left phylogeny) that showed limited genetic diversity compared to all the other hepacivirus lineages (right phylogeny). The colouring scheme follows the host assignment from main Figure 2.

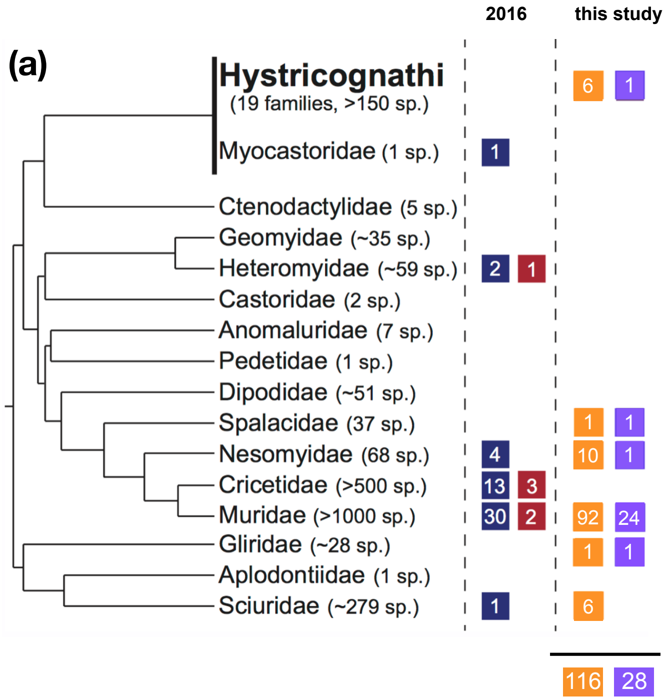

(b)

Country	Samples	Positives (%)
DRC	894	16 (1.79)
Ethiopia	426	4 (0.94)
Kenya	532	8 (1.50)
Madagascar	30	0
Mozambique	399	10 (2.51)
Tanzania	1,798	41 (2.28)
Zambia	224	1 (0.45)
	**4,303**	**80 (1.86)**

FIG. 3 **Sampling and screening summary of hepaciviruses in rodents.** **(a)** The phylogeny of the extant rodent families reproduced from (**?**) and adapted to include our current rodent sampling. The large rodent suborder Hystricognathi (in bold) has been collapsed into a single lineage for clarity. Boxes next to each family name demonstrate the estimated number of species that has been previously screened (blue box) and those found to harbour hepaciviruses in earlier studies (red box). The number of potential rodent species in our sampling is shown in orange boxes and the number of species within which we have detected hepaciviruses is indicated in the purple boxes. **(b)** The capture efforts were performed at multiple localities of seven African countries: the Democratic Republic of the Congo (DRC), Ethiopia, Kenya, Madagascar, Mozambique, Tanzania and Zambia. The number of sampled individuals is shown next to each country, along with the number of specimens that were found to be positive for hepaciviruses. In brackets we summarise the percentage of positives detected within the different countries.

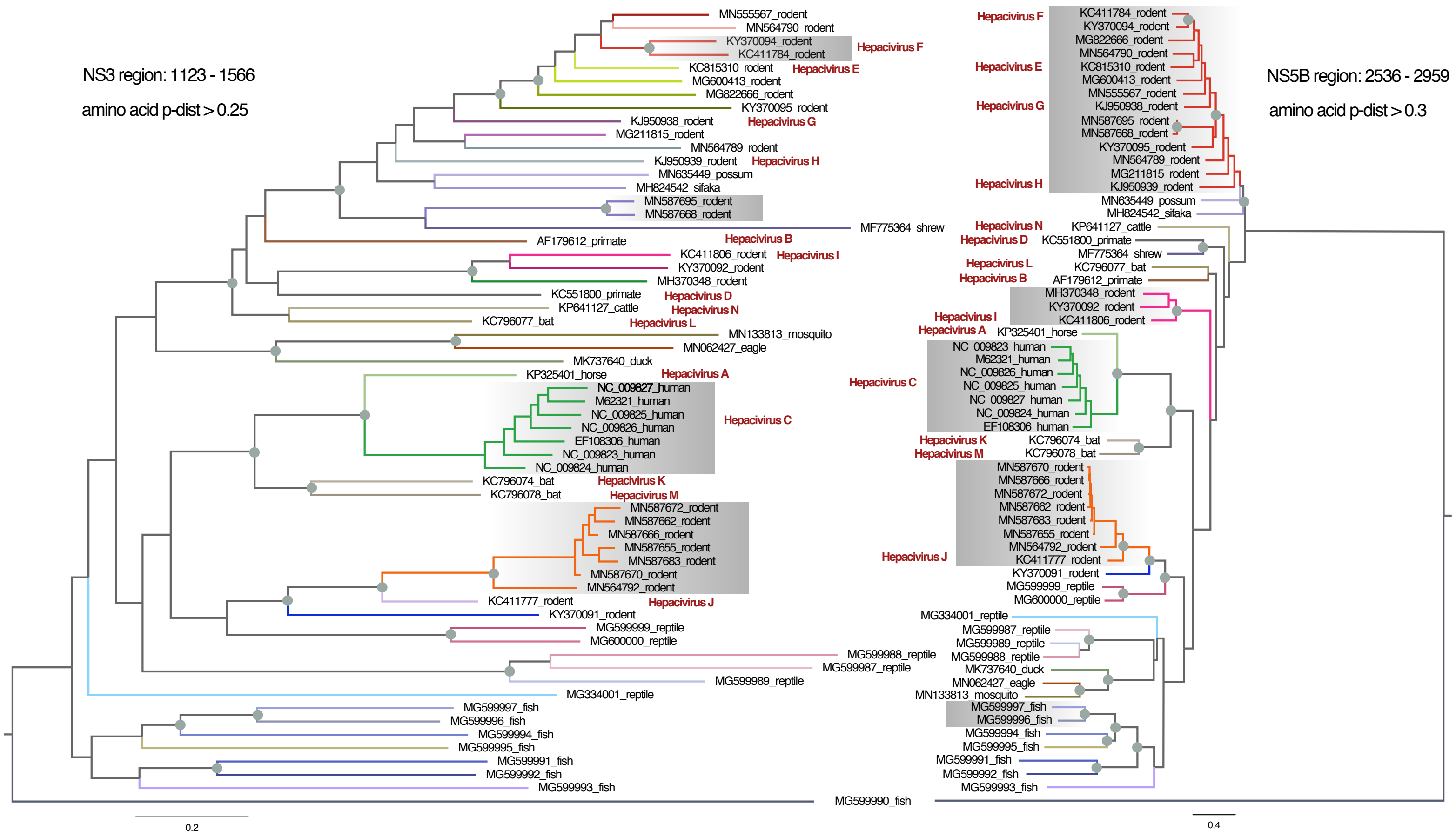

FIG. 4 ***Hepaciviruses* species classification following the taxonomic proposal by ?.** For this analysis we used a reduced data set of 60 genomes differing over their complete coding sequence by amino acid p-distances greater than 0.1 (more details in Supplementary Table 7). ML trees were generated for amino acid regions 1123 - 1566 and 2536 - 2959 (reference sequence: M62321). Demarcation between species occurs when amino acid p-distances in region 1123 - 1566 are greater than 0.25 and greater than 0.3 in region 2536 - 2959. Grey circles denote internal nodes with bootstrap support ≥70, while grey shaded areas indicate virus species with more than one representatives. The assigned virus species name is shown next to each lineage in bordeaux.

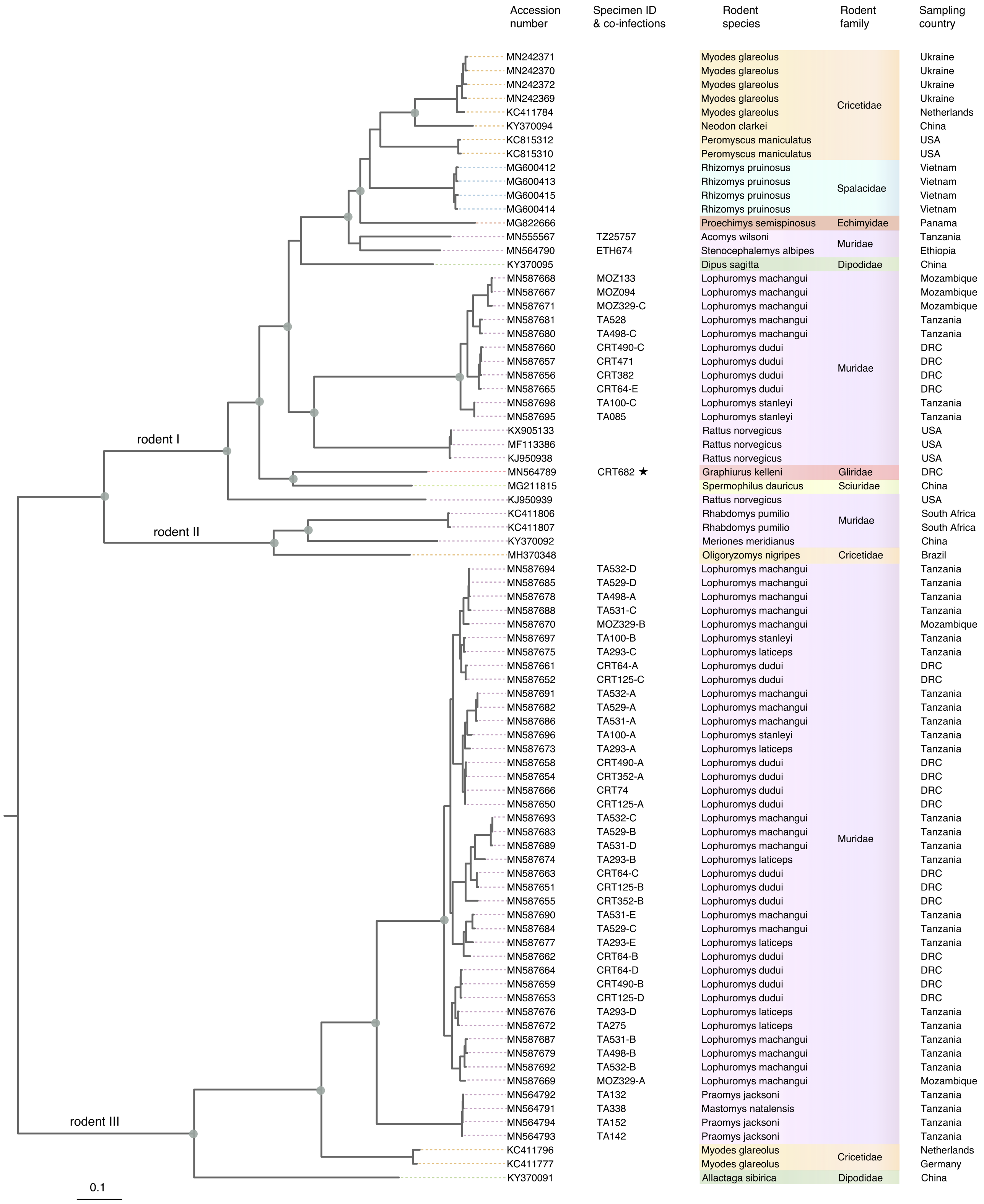

FIG. 5 **Phylogenetic reconstruction of rodent hepaciviruses.** ML tree of all available ($n = 26$) and novel ($n = 56$) hepacivirus genomes. Columns next to the phylogeny indicate GenBank accession numbers, specimen IDs and their associated RHV strains, the exact rodent species and family and the country of origin. Grey circles indicate internal nodes with bootstrap support ≥70. The star denotes isolate CRT682, that originated in a *Graphiurus kelleni* individual collected in the DRC.

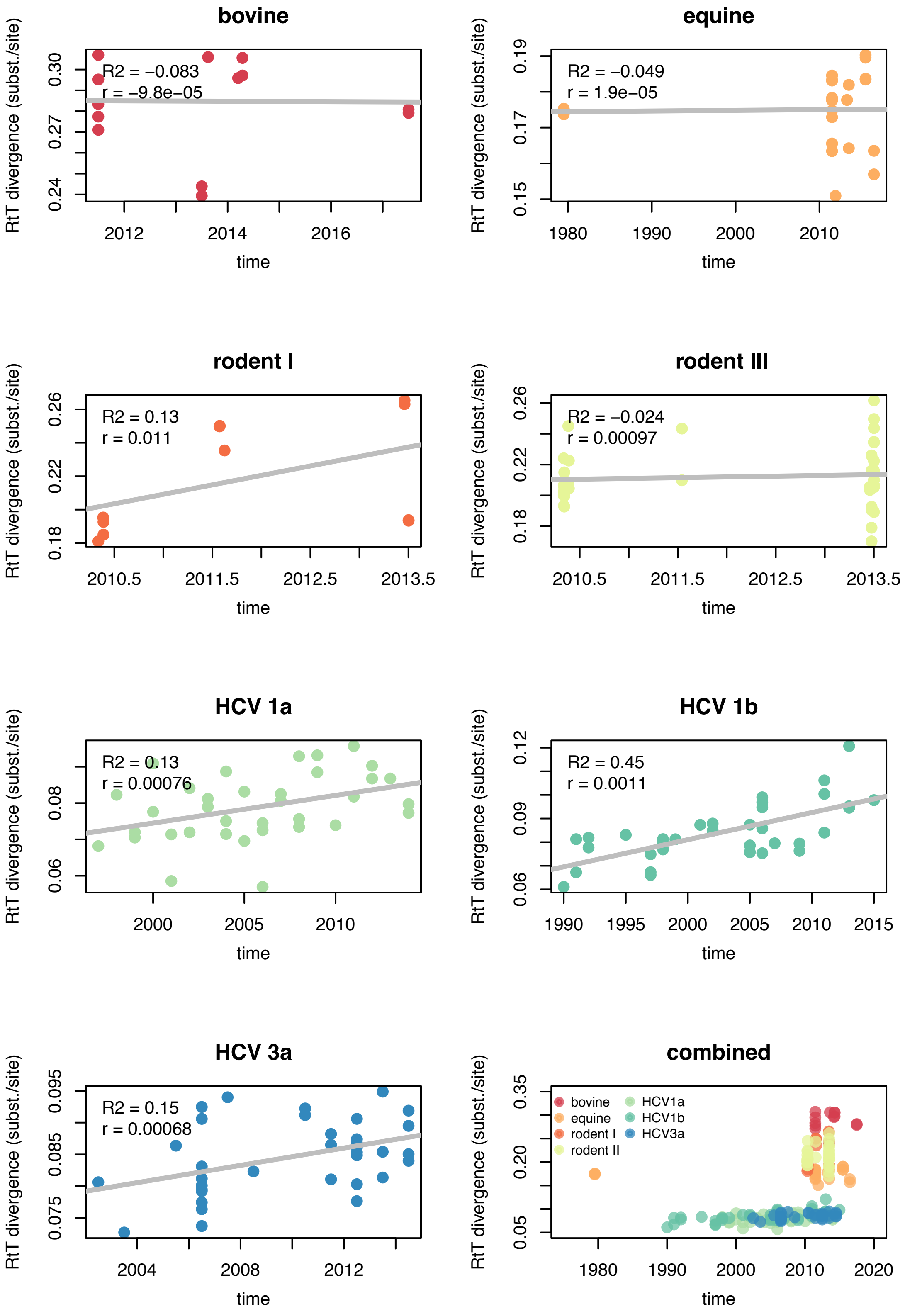

FIG. 6 **Temporal signal analysis of hepaciviruses.** For specific host lineages with limited diversity we show root-to-tip divergence as a function of sampling year. Co-efficients of determination (R2) and slope estimates are indicated in the upper left corner of the regression plots. Data points are coloured according to the specific host lineages: bovine (red), equine (orange), rodent I (coral), rodent III (lime green), HCV1a (light green), HCV1b (green), HCV3a (blue). In the final panel we plot all the root-to-tip divergences as a function of sampling times for all lineages.

- Supplementary Figure 1:

  Outer and inner primer pairs were designed targeting the most variable region of the rodent hepacivirus genome, as illustrated in Suppl. Figure 1.

- Supplementary Figure 2:

  To test for recombination, we selected lineage-specific data sets with limited and shallow diversity. Suppl. Figure 2 demonstrates the lineages used in our recombination and selection analyses, while the species composition of these viral genomes is detailed here:

  - Rodent hepaciviruses from four *Lophuromys* species were used: *L. dudui*, *L. laticeps*, *L. machangui* and *L. stanleyi*.
  - Equine hepaciviruses from two *Equus* species were used: *E. ferus* and *E. asinus*.
  - Bovine hepaciviruses included sequences only from the *Bos taurus* species.
  - Hepatitis C virus datasets were obviously restricted to the *Homo sapiens* species.

  A substantial number of lineage-specific recombinants were identified in rodents, with the highest proportion in strains circulating in the rodent III lineage. Furthermore, three hepaciviruses present in Ghanaian cattle (accession numbers: KP2655943, KP2655948, KP2655950) appeared to share a recombinant part between positions 4953 and 5064 with an ancestor of the German cattle hepacivirus MH027948. For the equine lineage, recombination events were detected between strains circulating in Europe (and the UK) with those circulating in the USA. The best supported recombination breakpoint was located between positions 6472 and 7608.

  We assume that the virus population dynamics remained the same within the different host genera. It is, therefore, conceivable that comparison of selective pressure acting on closely related viruses holds a biological explanation even when pathogens from congeneric species are being tested.

- Supplementary Figure 3:

  Panel a demonstrates the species range of our current rodent sampling, while panel b summarizes our screening results per African country along with the composition of our sample collection. For a detailed list of all hepaci-positive specimens we refer to Supplementary Table 6.

- Supplementary Figure 4:

  In an attempt to position our novel sequences within the tremendous heterogeneity of hepaciviruses, we followed the classification proposal by **?**. For this analysis we used a reduced data set of 60 genomes differing over their complete coding sequence by amino acid p-distances greater than 0.1 (Supplementary Table 7). This assignment resulted in 46 *Hepacivirus* species for the amino acid region 1123 - 1566 and in 31 *Hepacivirus* species for the amino acid region 2536 - 2959 (Supplementary Figure 4). Only 18 genomes were classified in groups of more than one representatives in the NS3 region, while 34 genomes were assigned in lineages with multiple hepaciviruses in the NS5B fragment. Although the tree topology remains congruent between those regions, the number of assigned *Hepacivirus* species differs dramatically. The current demarcation criteria not only fail to classify viruses from host-specific lineages into one single species, but also they provide erratic assignments between the proposed conserved positions. For example, major inconsistencies appear to exist in the rodent I cluster, which is divided into 15 distinct *Hepacivirus* species for amino acid region 1123 - 1566 compared to the two well-defined species for the other region.

- Supplementary Figure 5:

  Rodent hepacivirus phylogeny annotated with GenBank accession numbers, specimen IDs and their associated RHV strains, the exact rodent species and family and the country of origin.

- Supplementary Figure 6:

  Root-to-tip divergence of host-specific lineages as a function of their sampling year.

References

Pybus, O. G. and Thézé, J. 2016. Hepacivirus cross-species transmission and the origins of the hepatitis c virus. *Curr Opin Virol*, 16: 1–7.

Smith, D. B., Becher, P., Bukh, J., Gould, E. A., Meyers, G., Monath, T., Muerhoff, A. S., Pletnev, A., Rico-Hesse, R., Stapleton, J. T., and Simmonds, P. 2016. Proposed update to the taxonomy of the genera hepacivirus and pegivirus within the flaviviridae family. *J Gen Virol*, 97(11): 2894–2907.
