# Supplementary material for: Molecular detection and genomic characterization of diverse hepaciviruses in African rodents
Source: Virus Evol. 2021 Apr 12;7(1):veab036. doi: 10.1093/ve/veab036 (PMC8242229; doi:10.1093/ve/veab036)
Supplement: veab036_Supplementary_Data [file veab036_supplementary_data.zip › Table_S1_R1.docx]

**Supplementary table S1:** Complete list of specimens that have been screened for the presence of hepaciviruses.

| **Order** | **Family** | **Species** | **No. of samples** | **No. of positives** | **Sampling location** | **Sampling date** |
| --- | --- | --- | --- | --- | --- | --- |
|  |  |  |  |  |  |  |
|  |  |  |  |  |  |  |
| Afrosoricida | Chrysochloridae | *Chrysochloris stuhlmanni* | 2 |  | CD | 2010 |
|  | **Subtotal** | **1 species** | **2 samples** | **0 positives** |  |  |
| Carnivora | Herpestidae | *Atilax paludinosus* | 1 |  | TZ | 2007 |
|  | Viverridae | *Genetta angolensis* | 1 |  | TZ | 2011 |
|  | **Subtotal** | **2 species** | **2 samples** | **0 positives** |  |  |
| Chiroptera | Molossidae | *Chaerephon pumilus* | 3 |  | ZM/TZ | 2010/2013 |
|  |  | *Mops condylurus* | 4 | ­­ | MZ | 2011 |
|  | Pteropodidae | *Epomophorus gambianus* | 3 |  | ZM | 2010 |
|  |  | *Epomophorus labiatus* | 8 |  | MZ | 2011 |
|  |  | *Lissonycteris goliath* | 1 |  | MZ | 2011 |
|  |  | *Rousettus aegyptiacus* | 3 |  | KE | 2010 |
|  | Rhinolophidae | *Rhinolphus hilebrandtii­* | 2 |  | MZ | 2011 |
|  | Vespertilionidae | *Chalinolobus variegatus* | 1 |  | ZM | 2010 |
|  |  | *Glauconycteris atra* | 6 | 2 | TZ | 2013 |
|  |  | *Pipistrellus* sp. | 5 |  | ZM/KE/MZ | 2010/2010/2011 |
|  | **Subtotal** | **10 species** | **36 samples** | **2 positives** |  |  |
| Eulipotyphla | Erinaceidae | *Atelerix* sp. | 1 |  | TZ | 2013 |
|  | Soricidae | *Crocidura caliginea* | 15 |  | CD | 2012,2013 |
|  |  | *Crocidura* cf. *denti* | 34 |  | CD | 2012,2013 |
|  |  | *Crocidura* cf. *flavescens* | 4 |  | TZ | 2013 |
|  |  | *Crocidura* cf. *littoralis* | 18 |  | CD | 2012,2013 |
|  |  | *Crocidura* cf. *ludia* | 68 |  | CD | 2012,2013 |
|  |  | *Crocidura dolichura* | 10 |  | CD | 2012,2013 |
|  |  | *Crocidura fumosa* | 4 |  | KE | 2010 |
|  |  | *Crocidura hildegardeae* | 5 |  | TZ | 2013 |
|  |  | *Crocidura hirta* | 22 |  | ZM/MZ | 2009,2010/2011 |
|  |  | *Crocidura lamottei-parvipes group* | 3 |  | TZ | 2013 |
|  |  | *Crocidura luna* | 9 |  | ZM/TZ | 2010/2013 |
|  |  | *Crocidura montis* | 22 |  | TZ | 2013 |
|  |  | *Crocidura olivieri* | 46 |  | ZM/ET/KE/CD/TZ | 2009/2010/2010/2010,2012,2013/2013 |
|  |  | *Crocidura silacea-mariquensis* | 1 |  | ZM | 2010 |
|  |  | *Crocidura* sp. | 149 |  | ZM/MZ/TZ/CD | 2010/2011/2007-2013/2007,2010-2013 |
|  |  | *Crocidura* sp. *Chipata* | 5 |  | ZM | 2010 |
|  |  | *Crocidura* sp. *Kaoma* | 1 |  | ZM | 2010 |
|  |  | *Crocidura turba* | 8 |  | ZM/KE/TZ | 2009/2010/2013 |
|  |  | *Paracrocidura schoutedeni* | 5 |  | CD | 2012,2013 |
|  |  | *Scutisorex* *congicus* | 1 |  | CD | 2013 |
|  |  | *Scutisorex* sp. | 5 |  | CD | 2012 |
|  |  | *Suncus* sp. | 9 |  | CD | 2013 |
|  |  | *Surdisorex polulus* | 1 |  | KE | 2010 |
|  |  | *Sylvisorex akaibei* | 9 |  | CD | 2013 |
|  |  | *Sylvisorex* sp. | 4 |  | TZ/CD | 2006/2013 |
|  | **Subtotal** | **26 species** | **459 samples** | **0 positives** |  |  |
| Hyracoidea | Procaviidae | *Dendrohyrax dorsalis* | 1 |  | CD | 2012 |
|  | **Subtotal** | **1 species** | **1 sample** | **0 positives** |  |  |
| Macroscelidae | Macroscelididae | *Elephantulus brachyrhynchus* | 6 |  | ZM/KE/TZ | 2009/2010,2011/2013 |
|  |  | *Elephantulus rufescens* | 1 |  | KE | 2010 |
|  |  | *Petrodromus tetradactylus* | 1 |  | MZ | 2011 |
|  |  | *Petrodromus tordayi* | 6 |  | CD | 2013 |
|  |  | *Rhynchocyon cirnei* | 1 |  | MZ | 2011 |
|  | **Subtotal** | **5 species** | **15 samples** | **0 positives** |  |  |
| Rodentia | Bathyergidae | *Fukomys anselli* | 3 |  | ZM | 2010 |
|  |  | *Fukomys bocagei* | 1 |  | ZM | 2010 |
|  |  | *Fukomys mechowii* | 4 |  | ZM | 2009 |
|  |  | *Fukomys whytei* | 1 |  | ZM | 2009 |
|  |  | *Heliophobius argenteocinereus* | 29 | 1 | TZ/ZM/KE/MZ | 2008/2010/2010/2011 |
|  | Gliridae | *Graphiurus* sp. * | 25 | 2 | TZ/KE/CD/ZM/MZ | 2007,2008,2010,2013/2010/2010/2010/2011 |
|  | Muridae | *Acomys* aff. *percivali* | 4 |  | KE | 2010 |
|  |  | *Acomys ignitus* | 13 |  | KE | 2010 |
|  |  | *Acomys kempi* | 21 | 2 | KE | 2010 |
|  |  | *Acomys muzei* | 17 |  | ZM/TZ | 2009,2010/2013 |
|  |  | *Acomys ngurui* | 82 |  | MZ/TZ | 2011/2009-2011 |
|  |  | *Acomys percivali* | 6 |  | KE | 2010 |
|  |  | *Acomys* sp. | 20 | 1 | TZ | 2008-2010/2013 |
|  |  | *Acomys spinosissimus* | 31 |  | MZ | 2011 |
|  |  | *Acomys wilsoni* | 8 | 2 | KE | 2010,2011 |
|  |  | *Aethomys chrysophilus* | 74 |  | ZM/KE/MZ/TZ | 2009,2010/2010/2011/2007,2011,2013 |
|  |  | *Aethomys hindei* | 6 |  | KE/MZ | 2010,2011/2011 |
|  |  | *Aethomys kaiseri* | 30 |  | KE/TZ | 2010,2011/2013 |
|  |  | *Aethomys silindensis* | 7 |  | MZ | 2011 |
|  |  | *Arvicanthis niloticus* | 25 |  | ET | 2010 |
|  |  | *Arvicanthis nairobae* | 1 |  | TZ | 2013 |
|  |  | *Arvicanthis neumanni* | 9 |  | TZ | 2012 |
|  |  | *Arvicanthis* aff. *niloticus* | 2 |  | KE | 2011 |
|  |  | *Arvicanthis somalicus* | 6 |  | KE | 2010,2011 |
|  |  | *Arvicanthis* sp. | 55 |  | CD/TZ | 2007/2007-2009 |
|  |  | *Deomys ferrugineus* | 1 |  | TZ | 2013 |
|  |  | *Gerbilliscus* cf. *bayeri* | 2 |  | KE | 2011 |
|  |  | *Gerbilliscus* cf. *cosensi* | 2 |  | ZM/MZ | 2009/2011 |
|  |  | *Gerbilliscus* cf. *taborae* | 3 |  | TZ | 2013 |
|  |  | *Gerbilliscus kempi* | 2 |  | TZ | 2013 |
|  |  | *Gerbilliscus leucogaster* | 4 |  | ZM/TZ | 2009/2013 |
|  |  | *Gerbilliscus nigricaudus* | 2 |  | KE | 2011 |
|  |  | *Gerbilliscus phillipsi* | 1 |  | KE | 2011 |
|  |  | *Gerbilliscus* sp. | 34 | 1 | TZ/MZ | 2007-2010,2012/2011 |
|  |  | *Gerbilliscus vicinus* | 14 | 2 | KE/TZ | 2010,2011/2013 |
|  |  | *Grammomys* cf. *gazellae* | 1 |  | KE | 2010 |
|  |  | *Grammomys* cf. *kuru* | 3 |  | CD/TZ | 2013 |
|  |  | *Grammomys ibeanus* | 1 |  | TZ | 2007 |
|  |  | *Grammomys macmillani* | 16 |  | KE | 2010,2011 |
|  |  | *Grammomys* sp. | 83 | 1 | CD/TZ | 2010/2006,2007,2009-2011 |
|  |  | *Grammomys surdaster* | 9 |  | ZM/KE/TZ | 2009/2010/2013 |
|  |  | *Hybomys* cf. *univitattus* | 14 |  | CD/TZ | 2013 |
|  |  | *Hybomys lunaris* | 1 |  | CD | 2010 |
|  |  | *Hybomys* sp. | 40 | 1 | CD | 2010,2012,2013 |
|  |  | *Hylomyscus aeta* | 3 |  | CD | 2012 |
|  |  | *Hylomyscus anselli* | 1 |  | ZM | 2009 |
|  |  | *Hylomyscus endorobae* | 5 |  | KE | 2010 |
|  |  | *Hylomyscus* sp. | 45 |  | CD | 2010-2013 |
|  |  | *Hylomyscus stella* | 26 | 1 | KE/CD/TZ | 2010/2012/2013 |
|  |  | *Lemniscomys rosalia* | 13 |  | ZM/TZ | 2009/2007,2008,2013 |
|  |  | *Lemniscomys* sp. | 34 | 1 | TZ/CD/MZ | 2007,2008,2011/2010/2011 |
|  |  | *Lemniscomys striatus* | 50 | 1 | KE/TZ/CD | 2010,2011/2009,2013/2010 |
|  |  | *Lemniscomys zebra* | 43 |  | TZ | 2008,2009,2011-2013 |
|  |  | *Lophuromys ansorgei* | 2 |  | KE | 2010 |
|  |  | *Lophuromys dudui* | 10 | 8 | CD | 2012,2013 |
|  |  | *Lophuromys kilonzoi* | 33 | 5 | TZ | 2007 |
|  |  | *Lophuromys laticeps* | 13 | 3 | TZ | 2013 |
|  |  | *Lophuromys luteogaster* | 6 |  | CD | 2012,2013 |
|  |  | *Lophuromys machangui* | 30 | 21 | MZ/TZ | 2011/2013 |
|  |  | *Lophuromys* sp. | 118 | 1 | CD/TZ | 2007,2010,2012,2013/2006,2009,2010,2011 |
|  |  | *Lophuromys stanleyi* | 31 | 4 | TZ | 2013 |
|  |  | *Lophuromys zena* | 60 | 2 | KE | 2010,2011 |
|  |  | *Malacomys longipes* | 25 |  | ZM/CD | 2009/2010, 2012,2013 |
|  |  | *Mastomys awashensis* | 28 | 2 | ET | 2010/2012 |
|  |  | *Mastomys erythroleucus* | 53 |  | KE/ET | 2011/2012 |
|  |  | *Mastomys kollmannspergeri* | 1 |  | ET | 2012 |
|  |  | *Mastomys natalensis* | 643 | 1 | ZM/KE/MZ/ET/TZ | 2009,2010/2010,2011/2011/2012/2007-2009,2011-2013 |
|  |  | *Mastomys pernanus* | 4 |  | KE | 2010 |
|  |  | *Mastomys* sp. | 159 |  | CD/TZ | 2007,2010/2006,2007,2009-2011,2013 |
|  |  | *Micaelamys namaquensis* | 26 | 1 | TZ | 2011,2012 |
|  |  | *Mus bufo* | 17 |  | KE | 2010 |
|  |  | *Mus* cf. *gerbillus* | 1 |  | KE | 2010 |
|  |  | *Mus* cf. *gratus* | 18 |  | KE/CD/TZ | 2010/2012/2013 |
|  |  | *Mus mahomet* | 33 |  | ET | 2010 |
|  |  | *Mus minutoides* | 35 |  | TZ/ZM/KE/MZ | 2007,2013/2009,2010/2010/2011 |
|  |  | *Mus musculus* | 2 |  | KE | 2010 |
|  |  | *Mus* sp. | 103 |  | TZ/CD/MG | 2008-2010/2007,2010,2012,2013/2010 |
|  |  | *Mus triton* | 23 |  | ZM/KE/TZ | 2009/2011/2013 |
|  |  | *Myomyscus brockmani* | 36 |  | KE | 2010,2011 |
|  |  | *Oenomys hypoxanthus* | 11 |  | CD/KE/TZ | 2010/2010/2013 |
|  |  | *Oenomys* sp. | 3 |  | CD | 2010 |
|  |  | *Otomys* sp. | 6 |  | CD/TZ/KE | 2007/2007,2009,2010/2010 |
|  |  | *Otomys tropicalis* | 4 |  | KE | 2010 |
|  |  | *Praomys delectorum* | 103 |  | KE/TZ | 2010/2007,2013 |
|  |  | *Praomys jacksoni* | 140 | 7 | ZM/KE/CD/TZ | 2009/2010,2011/2010,2012/2013 |
|  |  | *Praomys lukoleae* | 6 |  | CD | 2013 |
|  |  | *Praomys minor* | 2 |  | ZM | 2009 |
|  |  | *Praomys* sp. | 390 | 2 | TZ/KE/CD | 2006,2008-2011/2010/2010,2012,2013 |
|  |  | *Rattus rattus* | 250 | 1 | CD/TZ/ZM/ET/KE/MG/MZ | 2007,2012,2013/2006,2007,2009/2009,2010/2010/2010/2010/2011 |
|  |  | *Rattus* sp. | 84 |  | TZ/MZ | 2007,2009,2010,2013/2011 |
|  |  | *Rhabdomys dilectus* | 27 |  | KE/TZ | 2010/2008,2009,2013 |
|  |  | *Stenocephalemys albipes* | 178 | 2 | ET | 2010/2012 |
|  |  | *Stenocephalemys albocaudatus* | 10 |  | ET | 2012 |
|  |  | *Stenocephalemys griseicauda* | 10 |  | ET | 2012 |
|  |  | *Stenocephalemys* sp. | 9 |  | ET | 2012 |
|  |  | *Stochomys longicaudatus* | 5 |  | CD | 2010,2012,2013 |
|  |  | *Uranomys ruddi* | 9 |  | MZ/TZ | 2011 |
|  |  | *Zelotomys hildegardeae* | 2 |  | TZ | 2013 |
|  | Nesomyidae | *Beamys hindei* | 4 |  | MZ | 2011 |
|  |  | *Beamys major* | 6 |  | TZ | 2007,2011 |
|  |  | *Cricetomys gambianus* | 6 |  | TZ | 2007 |
|  |  | *Cricetomys* sp. | 5 |  | CD/MZ | 2010/2011 |
|  |  | *Dendromus insignis* | 8 |  | KE | 2010,2011 |
|  |  | *Dendromus nyikae* | 8 |  | TZ | 2007 |
|  |  | *Eliurus myoxinus* | 5 |  | MG | 2010 |
|  |  | *Saccostomus campestris* | 13 | 1 | ZM/MZ | 2009,2010/2011 |
|  |  | *Saccostomus mearnsi* | 4 |  | KE | 2010, 2011 |
|  |  | *Saccostomus umbriventer* | 2 |  | KE | 2010 |
|  | Sciuridae | *Funisciurus anerythus* | 3 |  | CD | 2010 |
|  |  | *Funisciurus pyrrhopus* | 3 |  | CD | 2013 |
|  |  | *Funisciurus* sp. | 4 |  | CD | 2010 |
|  |  | *Heliosciurus rufobrachium* | 1 |  | TZ | 2013 |
|  |  | *Paraxerus ochraceus* | 6 |  | TZ/KE | 2007/2010 |
|  |  | *Xerus* sp. | 3 |  | TZ | 2013 |
|  | Spalacidae | *Tachyoryctes splendens* | 13 | 1 | KE/TZ | 2010,2011/2008,2013 |
|  | Thryonomyidae | *Thryonomys* sp. | 1 |  |  |  |
|  | **Subtotal** | **116 species** | **3788 samples** | **78 positives** |  |  |
|  | **Total** | **161 species** | **4303** | **80** |  |  |

* The current nomenclature does not reliably delineate the genetic lineages in the *Graphiurus* genus. Based on the experts’ opinion that co-authored this manuscript, *Graphiurus* individuals from those countries are genetically different and most likely represent multiple species. Although we cannot update the rodent species taxonomy within the scope of this manuscript, we will use the *Graphiurus* sp. name and count them all together as a single species.
