# Supplementary material for: Molecular detection and genomic characterization of diverse hepaciviruses in African rodents
Source: Virus Evol. 2021 Apr 12;7(1):veab036. doi: 10.1093/ve/veab036 (PMC8242229; doi:10.1093/ve/veab036)
Supplement: veab036_Supplementary_Data [file veab036_supplementary_data.zip › Table_S2_R1.docx]

**Supplementary table S2:** Genome assembly information and statistics of the generated hepacivirus genomes.

| **Accession**  **number** | **Isolate name** | **Length (bp)** | **Total reads** | **Mapped reads** | **Hepacivirus reads (%)** | **Mean depth (x)** |  |
| --- | --- | --- | --- | --- | --- | --- | --- |
|  |  |  |  |  |  |  |  |
| MN535729 | TA166/TZA/2013 | 8,877 | 32,384,088 | 7,369 | 0.02 | 84 |  |
| MN535730 | TA168/TZA/2013 | 8,864 | 48,290,982 | 15,025 | 0.03 | 222 |  |
| MN587650 | CRT125-A/COD/2010 | 10,589 | 38,980,104 | 67,324 | 0.17 | 783 |  |
| MN587651 | CRT125-B/COD/2010 | 10,198 | 38,980,104 | 47,629 | 0.12 | 569 |  |
| MN587652 | CRT125-C/COD/2010 | 9,556 | 38,980,104 | 67,214 | 0.17 | 859 |  |
| MN587653 | CRT125-D/COD/2010 | 7,378 | 38,980,104 | 21,855 | 0.06 | 349 |  |
| MN587654 | CRT352-A/COD/2010 | 10,798 | 38,665,546 | 26,753 | 0.07 | 311 |  |
| MN587655 | CRT352-B/COD/2010 | 10,218 | 38,665,546 | 32,703 | 0.08 | 401 |  |
| MN587656 | CRT382/COD/2010 | 9,202 | 19,188,476 | 20,676 | 0.11 | 222 |  |
| MN587657 | CRT471/COD/2010 | 9,093 | 34,484,982 | 49,037 | 0.14 | 692 |  |
| MN587658 | CRT490-A/COD/2010 | 10,786 | 40,271,194 | 32,478 | 0.08 | 385 |  |
| MN587659 | CRT490-B/COD/2010 | 10,413 | 40,271,194 | 25,152 | 0.06 | 307 |  |
| MN587660 | CRT490-C/COD/2010 | 9,092 | 40,271,194 | 43,912 | 0.11 | 629 |  |
| MN587661 | CRT64-A/COD/2010 | 10,417 | 35,044,868 | 32,846 | 0.09 | 411 |  |
| MN587662 | CRT64-B/COD/2010 | 10,056 | 35,044,868 | 16,557 | 0.05 | 212 |  |
| MN587663 | CRT64-C/COD/2010 | 9,846 | 35,044,868 | 39,061 | 0.11 | 485 |  |
| MN587664 | CRT64-D/COD/2010 | 9,616 | 35,044,868 | 29,537 | 0.08 | 363 |  |
| MN587665 | CRT64-E/COD/2010 | 9,111 | 35,044,868 | 20,274 | 0.06 | 285 |  |
| MN564789 | CRT682/COD/2010 | 8,905 | 64,046,542 | 26,664 | 0.04 | 286 |  |
| MN587666 | CRT74/COD/2010 | 10,299 | 78,066,330 | 96,359 | 0.12 | 1248 |  |
| MN564790 | ETH674/ETH/2012 | 7,857 | 18,743,010 | 558 | 0.00 | 8 |  |
| MN587667 | MOZ094/MOZ/2011 | 8,991 | 27,052,806 | 1,212 | 0.01 | 14 |  |
| MN587668 | MOZ133/MOZ/2011 | 8,061 | 26,434,678 | 446 | 0.00 | 6 |  |
| MN587669 | MOZ329-A/MOZ/2011 | 10,443 | 33,200,264 | 2,840 | 0.01 | 28 |  |
| MN587670 | MOZ329-B/MOZ/2011 | 9,805 | 33,200,264 | 2,867 | 0.01 | 30 |  |
| MN587671 | MOZ329-C/MOZ/2011 | 9,076 | 33,200,264 | 8,475 | 0.03 | 101 |  |
| MN587695 | TA085/TZA/2013 | 9,117 | 34,726,306 | 209,897 | 0.63 | 2715 |  |
| MN587696 | TA100-A/TZA/2013 | 10,732 | 29,063,502 | 10,449 | 0.04 | 119 |  |
| MN587697 | TA100-B/TZA/2013 | 9,532 | 29,063,502 | 21,205 | 0.07 | 245 |  |
| MN587698 | TA100-C/TZA/2013 | 9,109 | 29,063,502 | 33,589 | 0.12 | 425 |  |
| MN564792 | TA132/TZA/2013 | 9,802 | 27,975,436 | 36,582 | 0.13 | 483 |  |
| MN564793 | TA142/TZA/2013 | 9,853 | 15,236,960 | 50,451 | 0.33 | 500 |  |
| MN564794 | TA152/TZA/2013 | 9,826 | 15,461,838 | 10,493 | 0.07 | 106 |  |
| MN587672 | TA275/TZA/2013 | 9,788 | 35,575,010 | 82,933 | 0.23 | 1035 |  |
| MN587673 | TA293-A/TZA/2013 | 10,295 | 43,001,980 | 83,824 | 0.19 | 1011 |  |
| MN587674 | TA293-B/TZA/2013 | 9,616 | 43,001,980 | 53,222 | 0.12 | 665 |  |
| MN587675 | TA293-C/TZA/2013 | 8,782 | 43,001,980 | 52,073 | 0.12 | 714 |  |
| MN587676 | TA293-D/TZA/2013 | 8,777 | 43,001,980 | 85,905 | 0.20 | 1209 |  |
| MN587677 | TA293-E/TZA/2013 | 8,732 | 43,001,980 | 59,566 | 0.14 | 821 |  |
| MN564791 | TA338/TZA/2013 | 9,807 | 28,069,808 | 2,704 | 0.01 | 36 |  |
| MN587678 | TA498-A/TZA/2013 | 10,412 | 26,235,274 | 19,421 | 0.07 | 175 |  |
| MN587679 | TA498-B/TZA/2013 | 9,200 | 26,235,274 | 22,543 | 0.09 | 231 |  |
| MN587680 | TA498-C/TZA/2013 | 9,093 | 26,235,274 | 29,106 | 0.11 | 323 |  |
| MN587681 | TA528/TZA/2013 | 9,083 | 43,252,972 | 1,082 | 0.00 | 15 |  |
| MN587682 | TA529-A/TZA/2013 | 10,768 | 34,037,162 | 29,411 | 0.09 | 335 |  |
| MN587683 | TA529-B/TZA/2013 | 9,634 | 34,037,162 | 16,378 | 0.05 | 206 |  |
| MN587684 | TA529-C/TZA/2013 | 9,178 | 34,037,162 | 14,990 | 0.04 | 192 |  |
| MN587685 | TA529-D/TZA/2013 | 9,067 | 34,037,162 | 14,161 | 0.04 | 184 |  |
| MN587686 | TA531-A/TZA/2013 | 10,788 | 35,890,092 | 45,394 | 0.13 | 519 |  |
| MN587687 | TA531-B/TZA/2013 | 9,432 | 35,890,092 | 39,758 | 0.11 | 518 |  |
| MN587688 | TA531-C/TZA/2013 | 9,238 | 35,890,092 | 22,053 | 0.06 | 279 |  |
| MN587689 | TA531-D/TZA/2013 | 8,826 | 35,890,092 | 20,742 | 0.06 | 274 |  |
| MN587690 | TA531-E/TZA/2013 | 8,070 | 35,890,092 | 22,655 | 0.06 | 331 |  |
| MN587691 | TA532-A/TZA/2013 | 10,782 | 61,884,584 | 38,421 | 0.06 | 444 |  |
| MN587692 | TA532-B/TZA/2013 | 10,326 | 61,884,584 | 39,042 | 0.06 | 461 |  |
| MN587693 | TA532-C/TZA/2013 | 9,370 | 61,884,584 | 18,208 | 0.03 | 228 |  |
| MN587694 | TA532-D/TZA/2013 | 9,181 | 61,884,584 | 17,474 | 0.03 | 224 |  |
| MN555567 | TZ25757/TZA/2011 | 9,154 | 27,184,916 | 61,224 | 0.23 | 824 |  |
| **Average** |  |  | **35,239,834** | **34,479** | **0.10** | **433** | |
