# Supplementary material for: Molecular detection and genomic characterization of diverse hepaciviruses in African rodents
Source: Virus Evol. 2021 Apr 12;7(1):veab036. doi: 10.1093/ve/veab036 (PMC8242229; doi:10.1093/ve/veab036)
Supplement: veab036_Supplementary_Data [file veab036_supplementary_data.zip › Table_S3_R1.docx]

**Supplementary table S3:** Primers used in this study.

| **Name** | **Sequence (5'-3')** | **Amplicon size (bp)** | **Study** |
| --- | --- | --- | --- |
|  |  |  |  |
| AK4340F1 | GTACTTGCTACTGCNACNCC | 299 | Kapoor et al., 2013 |
| AK4360R1 | TACCCTGTCATAAGGGCRTC |  | Kapoor et al., 2013 |
| AK4340F2 | CTTGCTACTGCNACNCCWCC | 296 | Kapoor et al., 2013 |
| AK4360R2 | TACCCTGTCATAAGGGCRTCNGT |  | Kapoor et al., 2013 |
| MOZ094-5110F | ACTGGCTTAGGCTTAGGA | 287 | This study |
| MOZ094-5438R | AAGCAAGCCAAGCCAGCG |  | This study |
| MOZ133-582F | CTACAAGCCTATCCCTCTCA | 223 | This study |
| MOZ133-978R | ACTAGCGAGCCAACCAAA |  | This study |
| MOZ133-1741F | ACATTGTATCGCGGTTCG | 172 | This study |
| MOZ133-1918R | GTATTGCCATAATTACAATGTGG |  | This study |
| MOZ133-2085F | CTTAGGTTGGCTGCTCTGTA | 211 | This study |
| MOZ133-2301R | AGTGGTGACCTCCAGTTG |  | This study |
| MOZ133-2492F | TTGCTCGTCTGGCGGAGAA | 133 | This study |
| MOZ133-2837R | ACATCTAGGTCTTCATAGCC |  | This study |
| MOZ133-3159F | CTCATCAGGCGCTCCGTT | 195 | This study |
| MOZ133-3365R | GCAACAAGGATTTCGATGG |  | This study |
| MOZ133-3393F | ATTACCCATGGAATACTATAAACA | 161 | This study |
| MOZ133-3556R | AATACGTAAGCCGCGAGC |  | This study |
| MOZ133-4078F | TTCTCAATTTCATTGACCACA | 162 | This study |
| MOZ133-4410R | AACGCGTTGTTTCATGGTA |  | This study |
| MOZ133-4452F | TCTAGAGGCTAAAGCAGG | 137 | This study |
| MOZ133-4690R | CAAAGAGATCAATGGCCAC |  | This study |
| MOZ133-5248F | TCTATGTCAGAGTGGTCTCA | 174 | This study |
| MOZ133-5460R | AAGTAGCCTGATGATGGTTG |  | This study |
| MOZ133-5699F | TTGTACGTTCCACTTGTGG | 129 | This study |
| MOZ133-5920R | CTCCTCTGAGCTCATACTTT |  | This study |
| MOZ133-7434F | CCGTAACTGTAGAGCATCG | 93 | This study |
| MOZ133-7889R | AAGTGTACTGCTATCACCC |  | This study |
| MOZ133-7978F | CCTTACATAATCCAGGCCC | 170 | This study |
| MOZ133-8183R | CAGCTCAATAAGGTCTTTCC |  | This study |
| MOZ329-7156P1F1 | GTGATGAATGAGATCGAAAGTG | 385 | This study |
| MOZ329-7737P1R1 | GGATCTCAGCGCTGCTCT |  | This study |
| MOZ329-7156P2F1 | GGGTTGTATGAAAATGGTTCCT | 364 | This study |
| MOZ329-7737P2R1 | GGACTGTCTCCTCCTTCTCG |  | This study |
| MOZ329-7540P3F1 | GGGTCCTGTTGGGGAAAG | 119 | This study |
| MOZ329-8129P3R1^†^ | CGCTCCCGTCTGTCAAGC |  | This study |
| MOZ329-7205P1F2 | CAGCAAGCCGGGCGTCCAG | 223 | This study |
| MOZ329-7513P1R2 | TCTTCGAGGAGCCTCTTGAT |  | This study |
| MOZ329-7178P2F2 | TCGATCCACCAACCATGC | 184 | This study |
| MOZ329-7490P2R2 | GCAGACACTGGTTCCGGTT |  | This study |
| MOZ329-7553P3F2 | GAAAGAAACCCGAGGATCCG | 106 | This study |
| MOZ329-8129P3R2^†^ | CGCTCCCGTCTGTCAAGC |  | This study |
| TA100-7156P1F1 | AAACAGGATCAGGGGGTTG | 571 | This study |
| TA100-7737P1R1 | AGATTGCGTTCTCCACCTC |  | This study |
| TA100-7156P2F1 | GGTTTGTATGAGAATGGGTCC | 367 | This study |
| TA100-7737P2R1 | GGATTGTCTCCTCTTTCTCG |  | This study |
| TA100-7414P3F1 | AGACTGGACCCTGGCAAG | 185 | This study |
| TA100-8108P3R1 | CTGATCGAGCATGTCCATCA |  | This study |
| TA100-7205P1F2 | AACAGCTGAAGCCAGGGG | 300 | This study |
| TA100-7513P1R2 | CTGCGAGCATGTCCTTGAAT |  | This study |
| TA100-7178P2F2 | TCGATCCACCATCTATGCCT | 184 | This study |
| TA100-7490P2R2 | GCAGCTACTGGTTCTGGC |  | This study |
| TA100-7428P3F2 | CAAGTCACAATGCAGACAGG | 148 | This study |
| TA100-7852P3R2 | CAAGCTGGTACTTTATGTTGTAAT |  | This study |
| TA338-1157F | TCAATTGGTCTGTCAGGAAC | 388 | This study |
| TA338-1544R | AGAGTCGCTTTCGCCAAC |  | This study |
| TA338-2204F | ACCACTTCAGGGTCGGTG | 349 | This study |
| TA338-2552R | TAAGCCATTACGCCCTTG |  | This study |
| TA338-3303F | TCTAGCTCTTACTGGCAGG | 318 | This study |
| TA338-3620R | TGCATAACACCGTCACGC |  | This study |
|  |  |  |  |
| ^†^ denotes an identical primer sequence | |  |  |
