# Supplementary material for: Molecular detection and genomic characterization of diverse hepaciviruses in African rodents
Source: Virus Evol. 2021 Apr 12;7(1):veab036. doi: 10.1093/ve/veab036 (PMC8242229; doi:10.1093/ve/veab036)
Supplement: veab036_Supplementary_Data [file veab036_supplementary_data.zip › Table_S4_R1.docx]

**Supplementary table S4:** Specimen information of all available and novel hepacivirus genomes used in the phylogenetic analysis of study.

| **Accession**  **number** | **Isolate name** | **Host family** | | **Host species** | | **Host type** | | | **Country** | **Sampling year** | | | |
| --- | --- | --- | --- | --- | --- | --- | --- | --- | --- | --- | --- | --- | --- |
|  |  |  |  |  |  |  |  |  |  |  |  |  |  |
| AB863589 | JPN3 | Equidae | | *Equus ferus caballus* | | Horse | | | Japan | 2013 | | | |
| AF179612 | GBV-B | Cebidae | | *Saguinus mystax* | | Primate | | | NA | 1995 | | | |
| NC_004102 | HCV1 | Hominidae | | *Homo sapiens* | | Human | | | NA | NA | | | |
| NC_009823 | HCV2 | Hominidae | | *Homo sapiens* | | Human | | | NA | NA | | | |
| NC_009824 | HCV3 | Hominidae | | *Homo sapiens* | | Human | | | New Zealand | NA | | | |
| NC_009825 | HCV4 | Hominidae | | *Homo sapiens* | | Human | | | Egypt | NA | | | |
| NC_009826 | HCV5 | Hominidae | | *Homo sapiens* | | Human | | | United Kingdom | NA | | | |
| NC_009827 | HCV6 | Hominidae | | *Homo sapiens* | | Human | | | NA | NA | | | |
| EF108306 | HCV7 | Hominidae | | *Homo sapiens* | | Human | | | Canada | 2003 | | | |
| JF744991 | NA | Canidae | | *Canis lupus familiaris* | | Dog | | | USA | 2011 | | | |
| JQ434001 | NZP-1 | Equidae | | *Equus ferus caballus* | | Horse | | | USA | 2011 | | | |
| JQ434002 | G1-073 | Equidae | | *Equus ferus caballus* | | Horse | | | USA | 2011 | | | |
| JQ434003 | A6-006 | Equidae | | *Equus ferus caballus* | | Horse | | | USA | 2011 | | | |
| JQ434004 | B10-022 | Equidae | | *Equus ferus caballus* | | Horse | | | USA | 2011 | | | |
| JQ434005 | F8-068 | Equidae | | *Equus ferus caballus* | | Horse | | | USA | 2011 | | | |
| JQ434006 | G5-077 | Equidae | | *Equus ferus caballus* | | Horse | | | USA | 2011 | | | |
| JQ434007 | H10-094 | Equidae | | *Equus ferus caballus* | | Horse | | | USA | 2011 | | | |
| JQ434008 | H3-011 | Equidae | | *Equus ferus caballus* | | Horse | | | UK | 2011 | | | |
| JX948116 | EF369_11J | Equidae | | *Equus ferus caballus* | | Horse | | | UK | 2011 | | | |
| KC411777 | RMU10-3382 | Cricetidae | | *Myodes glareolus* | | Rodent | | | Germany | 2010 | | | |
| KC411784 | NLR07-oct70 | Cricetidae | | *Myodes glareolus* | | Rodent | | | Netherlands | 2007 | | | |
| KC411796 | NLR08-365 | Cricetidae | | *Myodes glareolus* | | Rodent | | | Netherlands | 2008 | | | |
| KC411806 | SAR-3 | Muridae | | *Rhabdomys pumilio* | | Rodent | | | South Africa | 2008 | | | |
| KC411807 | SAR-46 | Muridae | | *Rhabdomys pumilio* | | Rodent | | | South Africa | 2008 | | | |
| KC551800 | GHV-1_BWC08 | Cercopithecidae | | *Colobus guereza* | | Primate | | | Uganda | 2010 | | | |
| KC551801 | GHV-1_BWC05 | Cercopithecidae | | *Colobus guereza* | | Primate | | | Uganda | 2010 | | | |
| KC551802 | GHV-2_BWC04 | Cercopithecidae | | *Colobus guereza* | | Primate | | | Uganda | 2010 | | | |
| KC796074 | PDB-829 | Hipposideridae | | *Hipposideros vittatus* | | Bat | | | Kenya | 2011 | | | |
| KC796077 | PDB-112 | Hipposideridae | | *Hipposideros vittatus* | | Bat | | | Kenya | 2010 | | | |
| KC796078 | PDB-491.1 | Molossidae | | *Otomops martiensseni* | | Bat | | | Kenya | 2011 | | | |
| KC796090 | PDB-452 | Molossidae | | *Otomops martiensseni* | | Bat | | | Kenya | 2010 | | | |
| KC796091 | PDB-445 | Molossidae | | *Otomops martiensseni* | | Bat | | | Kenya | 2010 | | | |
| KC815310 | RHV-339 | Cricetidae | | *Peromyscus maniculatus* | | Rodent | | | USA | 2008 | | | |
| KC815312 | RHV-089 | Cricetidae | | *Peromyscus maniculatus* | | Rodent | | | USA | 2008 | | | |
| KF177391 | DH1 | Equidae | | *Equus ferus caballus* | | Horse | | | Hungary | 2013 | | | |
| KJ472766 | WSU-2013 | Equidae | | *Equus ferus caballus* | | Horse | | | USA | 2013 | | | |
| KJ950938 | NrHV-1_NYC-C12 | Muridae | | *Rattus norvegicus* | | Rodent | | | USA | 2013 | | | |
| KJ950939 | NrHV-2_NYC-E43 | Muridae | | *Rattus norvegicus* | | Rodent | | | USA | 2012 | | | |
| KP265943 | GHC25 | Bovidae | | *Bos taurus* | | Cattle | | | Ghana | 2011 | | | |
| KP265946 | GHC52 | Bovidae | | *Bos taurus* | | Cattle | | | Ghana | 2011 | | | |
| KP265947 | GHC55 | Bovidae | | *Bos taurus* | | Cattle | | | Ghana | 2011 | | | |
| KP265948 | GHC85 | Bovidae | | *Bos taurus* | | Cattle | | | Ghana | 2011 | | | |
| KP265950 | GHC100 | Bovidae | | *Bos taurus* | | Cattle | | | Ghana | 2011 | | | |
| KP325401 | NZP-1 | Equidae | | *Equus ferus caballus* | | Horse | | | USA | 2011 | | | |
| KP641123 | B1 | Bovidae | | *Bos taurus* | | Cattle | | | Germany | 2013 | | | |
| KP641124 | 209 | Bovidae | | *Bos taurus* | | Cattle | | | Germany | 2014 | | | |
| KP641125 | 379 | Bovidae | | *Bos taurus* | | Cattle | | | Germany | 2014 | | | |
| KP641126 | 438 | Bovidae | | *Bos taurus* | | Cattle | | | Germany | 2014 | | | |
| KP641127 | 463 | Bovidae | | *Bos taurus* | | Cattle | | | Germany | 2014 | | | |
| MK737639 | HCL-1 | Anatidae | | *Anas platyrhynchos domesticus* | | Bird | | | China | 2018 | | | |
| MK737640 | HCL-2 | Anatidae | | *Anas platyrhynchos domesticus* | | Bird | | | China | 2018 | | | |
| MK737641 | HCL-3 | Anatidae | | *Anas platyrhynchos domesticus* | | Bird | | | China | 2018 | | | |
| MF775364 | SZCDC70 | Soricidae | | *Suncus murinus* | | Shrew | | | China | 2015 | | | |
| MH824541 | H2-L41 | Indriidae | | *Propithecus diadema* | | Primate | | | Madagascar | 2011 | | | |
| MH824540 | H2-L40 | Indriidae | | *Propithecus diadema* | | Primate | | | Madagascar | 2011 | | | |
| MH824539 | H2-L25 | Indriidae | | *Propithecus diadema* | | Primate | | | Madagascar | 2011 | | | |
| MH824542 | H6-L83 | Indriidae | | *Propithecus diadema* | | Primate | | | Madagascar | 2013 | | | |
| MH824543 | H5-L75 | Indriidae | | *Propithecus diadema* | | Primate | | | Madagascar | 2012 | | | |
| MH027948 | BH181 | Bovidae | | *Bos taurus* | | Cattle | | | Germany | NA | | | |
| MH027953 | BH204 | Bovidae | | *Bos taurus* | | Cattle | | | Germany | NA | | | |
| MG781019 | BR_RN034B019 | Bovidae | | *Bos taurus* | | Cattle | | | Brazil | 2013 | | | |
| MG781018 | BR_MA236B017 | Bovidae | | *Bos taurus* | | Cattle | | | Brazil | 2013 | | | |
| MG257793 | BovHepV/GD/01 | Bovidae | | *Bos taurus* | | Cattle | | | China | 2017 | | | |
| MG257794 | BovHepV/GD/02 | Bovidae | | *Bos taurus* | | Cattle | | | China | 2017 | | | |
| MH027992 | H56 | Equidae | | *Equus ferus caballus* | | Horse | | | Germany | NA | | | |
| MH028007 | H628 | Equidae | | *Equus ferus caballus* | | Horse | | | Germany | NA | | | |
| MH028000 | H268 | Equidae | | *Equus ferus caballus* | | Horse | | | Germany | NA | | | |
| MH027993 | H57 | Equidae | | *Equus ferus caballus* | | Horse | | | Germany | NA | | | |
| MH028004 | H581 | Equidae | | *Equus ferus caballus* | | Horse | | | Germany | NA | | | |
| MH028005 | H593 | Equidae | | *Equus ferus caballus* | | Horse | | | Germany | NA | | | |
| MH027998 | H170 | Equidae | | *Equus ferus caballus* | | Horse | | | Germany | NA | | | |
| KX056116 | K-061 | Equidae | | *Equus ferus caballus* | | Horse | | | South Korea | 2015 | | | |
| KX056117 | K-062 | Equidae | | *Equus ferus caballus* | | Horse | | | South Korea | 2015 | | | |
| MH027995 | H105 | Equidae | | *Equus ferus caballus* | | Horse | | | Germany | NA | | | |
| MH027999 | H179 | Equidae | | *Equus ferus caballus* | | Horse | | | Germany | NA | | | |
| MH028001 | H285 | Equidae | | *Equus ferus caballus* | | Horse | | | Germany | NA | | | |
| MH028002 | H286 | Equidae | | *Equus ferus caballus* | | Horse | | | Germany | NA | | | |
| MH027996 | H143 | Equidae | | *Equus ferus caballus* | | Horse | | | Germany | NA | | | |
| KX421286 | B82 | Equidae | | *Equus asinus asinus* | | Donkey | | | Bulgaria | 2015 | | | |
| KX421287 | B89 | Equidae | | *Equus asinus asinus* | | Donkey | | | Bulgaria | 2015 | | | |
| KT880191 | R09-249 | Equidae | | *Equus asinus asinus* | | Donkey | | | France | 1979 | | | |
| KT880192 | R09-250 | Equidae | | *Equus asinus asinus* | | Donkey | | | France | 1979 | | | |
| KT880193 | R09-251 | Equidae | | *Equus asinus asinus* | | Donkey | | | France | 1979 | | | |
| MF152651 | Guangzhou/6 | Equidae | | *Equus ferus caballus* | | Horse | | | China | 2016 | | | |
| MF152652 | Guangzhou/33 | Equidae | | *Equus ferus caballus* | | Horse | | | China | 2016 | | | |
| KY370095 | IM2014 | Dipodidae | | *Dipus sagitta* | | Rodent | | | China | 2014 | | | |
| KY370094 | Tibet2014 | Cricetidae | | *Neodon clarkei* | | Rodent | | | China | 2014 | | | |
| KY370092 | IM2014 | Muridae | | *Meriones meridianus* | | Rodent | | | China | 2014 | | | |
| KX905133 | rn-1 | Muridae | | *Rattus norvegicus* | | Rodent | | | USA | 2015 | | | |
| MF113386 | SD-1 | Muridae | | *Rattus norvegicus* | | Rodent | | | USA | 2014 | | | |
| MH370348 | On/2012 | Cricetidae | | *Oligoryzomys nigripes* | | Rodent | | | Brazil | 2012 | | | |
| MG211815 | GS2015 | Sciuridae | | *Spermophilus dauricus* | | Rodent | | | China | 2015 | | | |
| MG600414 | 05VZ-14-118 | Spalacidae | | *Rhizomys pruinosus* | | Rodent | | | Vietnam | 2015 | | | |
| MG600412 | 05VZ-14-104 | Spalacidae | | *Rhizomys pruinosus* | | Rodent | | | Vietnam | 2015 | | | |
| MG600413 | 05VZ-14-103 | Spalacidae | | *Rhizomys pruinosus* | | Rodent | | | Vietnam | 2015 | | | |
| MG600415 | 05VZ-14-119 | Spalacidae | | *Rhizomys pruinosus* | | Rodent | | | Vietnam | 2015 | | | |
| MG822666 | B349/PAN/2014 | Echimyidae | | *Proechimys semispinosus* | | Rodent | | | Panama | 2014 | | | |
| KY370091 | IM2014 | Dipodidae | | *Allactaga sibirica* | | Rodent | | | China | 2014 | | | |
| MG599988 | PXJHG3419 | Eublepharidae | | *Goniurosaurus luii* | | Lizard | | | China | NA | | | |
| MG599987 | YLSHG5584 | Sphaerodactylidae | | *Teratoscincus roborowskii* | | Lizard | | | China | NA | | | |
| MG599992 | DHHHBHGS10983 | Urolophidae | | *Urolophus aurantiacus* | | Cartilaginous fish | | | China | NA | | | |
| MG599997 | NHYJG60710 | Chimaeridae | | *Chimaera sp* | | Cartilaginous fish | | | China | NA | | | |
| MG599989 | LPXYG13170 | Gekkonidae | | *Hemidactylus bowringii* | | Lizard | | | China | NA | | | |
| MG599993 | FZFYG124617 | Protopteridae | | *Protopterus annectens* | | Lungfish | | | Nigeria | NA | | | |
| MG599991 | BWLTYG5315 | Rhinobatidae | | *Rhinobatos hynnicephalus* | | Cartilaginous fish | | | China | NA | | | |
| MG599994 | NHJSG30635 | Triakidae | | *Mustelus manazo* | | Cartilaginous fish | | | China | NA | | | |
| MG599998 | RBCSG7845 | Triakidae | | *Mustelus manazo* | | Cartilaginous fish | | | China | NA | | | |
| MG334001 | Hepacivirus sp. | Emydidae | | *Trachemys scripta elegans* | | Turtle | | | USA | 2008 | | | |
| MG599999 | WHJYGF75270 | Trionychidae | | *Pelodiscus sinensis* | | Turtle | | | China | NA | | | |
| MG599995 | NHJSG30261 | Squalidae | | *Squalus brevirostris* | | Cartilaginous fish | | | China | NA | | | |
| MG599996 | NHYJG60722 | Chimaeridae | | *Chimaera sp* | | Cartilaginous fish | | | China | NA | | | |
| MG600000 | WHWGGF64311 | Geoemydinae | | *Mauremys megalocephala* | | Turtle | | | China | NA | | | |
| MG599990 | XMLMGHepa10640 | Muraenidae | | *Gymnothorax reticularis* | | Ray-finned fish | | | China | NA | | | |
| MN635449 | VERT31 | Phalangeridae | | *Trichosurus vulpecula* | | Possum | | | Australia | NA | | | |
| MN062427 | NA03-001 | Accipitridae | | *Haliaeetus leucocephalus* | | Bird | | | USA | 2002 | | | |
| MH844500 | B32 | Bradypodidae | | *Bradypus variegatus* | | Sloth | | | Costa Rica | 2014 | | | |
| MH844501 | B31 | Bradypodidae | | *Bradypus variegatus* | | Sloth | | | Costa Rica | 2014 | | | |
| MH242369 | MgHV2 | Cricetidae | | *Myodes glareolus* | | Rodent | | | Ukraine | 2016 | | | |
| MH242370 | MgHV3 | Cricetidae | | *Myodes glareolus* | | Rodent | | | Ukraine | 2016 | | | |
| MH242371 | MgHV4 | Cricetidae | | *Myodes glareolus* | | Rodent | | | Ukraine | 2016 | | | |
| MH242372 | MgHV5 | Cricetidae | | *Myodes glareolus* | | Rodent | | | Ukraine | 2016 | | | |
| MT135177 | GD-61 | Anatidae | | *Anas platyrhynchos domesticus* | | Bird | | | China | 2019 | | | |
| MN133813 | P1-10 | Culicidae | | *Culex annulirostris* | | Mosquito | | | Australia | 2018 | | | |
| MT371434 |  | Galagidae | | *Galago senegalensis* | | Primate | | | NA | NA | | | |
| MT371438 |  | Indriidae | | *Propithecus diadema* | | Primate | | | Madagascar | NA | | | |
| MT371439 |  | Gekkonidae | | *Gehyra lauta* | | Lizard | | | Australia | 2013 | | | |
| MT371440 |  | Ixodidae | | *Ixodes holocyclus* | | Tick | | | Australia | 2016 | | | |
| MT371441 |  | Monarchidae | | *Grallina cyanoleuca* | | Bird | | | Australia | 2013 | | | |
| MT371442 |  | Pelecanidae | | *Pelecanus conspicillatus* | | Bird | | | Australia | 2013 | | | |
| MT371443 |  | Phascolarctidae | | *Phascolarctos cinereus* | | Marsupial | | | Australia | NA | | | |
| **Subtotal** | **130 available genomes** | |  | |  | |  |  | | |  | |  |
|  |  |  | |  | |  | | |  |  | |  |  |
| MN535729 | TA166/TZA/2013 | Vespertillionidae | | *Glauconycteris atra* | | Bat | | | Tanzania | 2013 | |  |  |
| MN535730 | TA168/TZA/2013 | Vespertillionidae | | *Glauconycteris atra* | | Bat | | | Tanzania | 2013 | |  |  |
| MN587650 | CRT125-A/COD/2010 | Muridae | | *Lophuromys dudui* | | Rodent | | | DRC | 2010 | |  |  |
| MN587651 | CRT125-B/COD/2010 | Muridae | | *Lophuromys dudui* | | Rodent | | | DRC | 2010 | |  |  |
| MN587652 | CRT125-C/COD/2010 | Muridae | | *Lophuromys dudui* | | Rodent | | | DRC | 2010 | |  |  |
| MN587653 | CRT125-D/COD/2010 | Muridae | | *Lophuromys dudui* | | Rodent | | | DRC | 2010 | |  |  |
| MN587654 | CRT352-A/COD/2010 | Muridae | | *Lophuromys dudui* | | Rodent | | | DRC | 2010 | |  |  |
| MN587655 | CRT352-B/COD/2010 | Muridae | | *Lophuromys dudui* | | Rodent | | | DRC | 2010 | |  |  |
| MN587656 | CRT382/COD/2010 | Muridae | | *Lophuromys dudui* | | Rodent | | | DRC | 2010 | |  |  |
| MN587657 | CRT471/COD/2010 | Muridae | | *Lophuromys dudui* | | Rodent | | | DRC | 2010 | |  |  |
| MN587658 | CRT490-A/COD/2010 | Muridae | | *Lophuromys dudui* | | Rodent | | | DRC | 2010 | |  |  |
| MN587659 | CRT490-B/COD/2010 | Muridae | | *Lophuromys dudui* | | Rodent | | | DRC | 2010 | |  |  |
| MN587660 | CRT490-C/COD/2010 | Muridae | | *Lophuromys dudui* | | Rodent | | | DRC | 2010 | |  |  |
| MN587661 | CRT64-A/COD/2010 | Muridae | | *Lophuromys dudui* | | Rodent | | | DRC | 2010 | |  |  |
| MN587662 | CRT64-B/COD/2010 | Muridae | | *Lophuromys dudui* | | Rodent | | | DRC | 2010 | |  |  |
| MN587663 | CRT64-C/COD/2010 | Muridae | | *Lophuromys dudui* | | Rodent | | | DRC | 2010 | |  |  |
| MN587664 | CRT64-D/COD/2010 | Muridae | | *Lophuromys dudui* | | Rodent | | | DRC | 2010 | |  |  |
| MN587665 | CRT64-E/COD/2010 | Muridae | | *Lophuromys dudui* | | Rodent | | | DRC | 2010 | |  |  |
| MN564789 | CRT682/COD/2010 | Gliridae | | *Graphiurus kelleni* | | Rodent | | | DRC | 2010 | |  |  |
| MN587666 | CRT74/COD/2010 | Muridae | | *Lophuromys dudui* | | Rodent | | | DRC | 2010 | |  |  |
| MN564790 | ETH674/ETH/2012 | Muridae | | *Stenocephalemys albipes* | | Rodent | | | Ethiopia | 2012 | |  |  |
| MN587667 | MOZ094/MOZ/2011 | Muridae | | *Lophuromys machangui* | | Rodent | | | Mozambique | 2011 | |  |  |
| MN587668 | MOZ133/MOZ/2011 | Muridae | | *Lophuromys machangui* | | Rodent | | | Mozambique | 2011 | |  |  |
| MN587669 | MOZ329-A/MOZ/2011 | Muridae | | *Lophuromys machangui* | | Rodent | | | Mozambique | 2011 | |  |  |
| MN587670 | MOZ329-B/MOZ/2011 | Muridae | | *Lophuromys machangui* | | Rodent | | | Mozambique | 2011 | |  |  |
| MN587671 | MOZ329-C/MOZ/2011 | Muridae | | *Lophuromys machangui* | | Rodent | | | Mozambique | 2011 | |  |  |
| MN587695 | TA085/TZA/2013 | Muridae | | *Lophuromys stanleyi* | | Rodent | | | Tanzania | 2013 | |  |  |
| MN587696 | TA100-A/TZA/2013 | Muridae | | *Lophuromys stanleyi* | | Rodent | | | Tanzania | 2013 | |  |  |
| MN587697 | TA100-B/TZA/2013 | Muridae | | *Lophuromys stanleyi* | | Rodent | | | Tanzania | 2013 | |  |  |
| MN587698 | TA100-C/TZA/2013 | Muridae | | *Lophuromys stanleyi* | | Rodent | | | Tanzania | 2013 | |  |  |
| MN564792 | TA132/TZA/2013 | Muridae | | *Praomys jacksoni* | | Rodent | | | Tanzania | 2013 | |  |  |
| MN564793 | TA142/TZA/2013 | Muridae | | *Praomys jacksoni* | | Rodent | | | Tanzania | 2013 | |  |  |
| MN564794 | TA152/TZA/2013 | Muridae | | *Praomys jacksoni* | | Rodent | | | Tanzania | 2013 | |  |  |
| MN587672 | TA275/TZA/2013 | Muridae | | *Lophuromys laticeps* | | Rodent | | | Tanzania | 2013 | |  |  |
| MN587673 | TA293-A/TZA/2013 | Muridae | | *Lophuromys laticeps* | | Rodent | | | Tanzania | 2013 | |  |  |
| MN587674 | TA293-B/TZA/2013 | Muridae | | *Lophuromys laticeps* | | Rodent | | | Tanzania | 2013 | |  |  |
| MN587675 | TA293-C/TZA/2013 | Muridae | | *Lophuromys laticeps* | | Rodent | | | Tanzania | 2013 | |  |  |
| MN587676 | TA293-D/TZA/2013 | Muridae | | *Lophuromys laticeps* | | Rodent | | | Tanzania | 2013 | |  |  |
| MN587677 | TA293-E/TZA/2013 | Muridae | | *Lophuromys laticeps* | | Rodent | | | Tanzania | 2013 | |  |  |
| MN564791 | TA338/TZA/2013 | Muridae | | *Mastomys natalensis* | | Rodent | | | Tanzania | 2013 | |  |  |
| MN587678 | TA498-A/TZA/2013 | Muridae | | *Lophuromys machangui* | | Rodent | | | Tanzania | 2013 | |  |  |
| MN587679 | TA498-B/TZA/2013 | Muridae | | *Lophuromys machangui* | | Rodent | | | Tanzania | 2013 | |  |  |
| MN587680 | TA498-C/TZA/2013 | Muridae | | *Lophuromys machangui* | | Rodent | | | Tanzania | 2013 | |  |  |
| MN587681 | TA528/TZA/2013 | Muridae | | *Lophuromys machangui* | | Rodent | | | Tanzania | 2013 | |  |  |
| MN587682 | TA529-A/TZA/2013 | Muridae | | *Lophuromys machangui* | | Rodent | | | Tanzania | 2013 | |  |  |
| MN587683 | TA529-B/TZA/2013 | Muridae | | *Lophuromys machangui* | | Rodent | | | Tanzania | 2013 | |  |  |
| MN587684 | TA529-C/TZA/2013 | Muridae | | *Lophuromys machangui* | | Rodent | | | Tanzania | 2013 | |  |  |
| MN587685 | TA529-D/TZA/2013 | Muridae | | *Lophuromys machangui* | | Rodent | | | Tanzania | 2013 | |  |  |
| MN587686 | TA531-A/TZA/2013 | Muridae | | *Lophuromys machangui* | | Rodent | | | Tanzania | 2013 | |  |  |
| MN587687 | TA531-B/TZA/2013 | Muridae | | *Lophuromys machangui* | | Rodent | | | Tanzania | 2013 | |  |  |
| MN587688 | TA531-C/TZA/2013 | Muridae | | *Lophuromys machangui* | | Rodent | | | Tanzania | 2013 | |  |  |
| MN587689 | TA531-D/TZA/2013 | Muridae | | *Lophuromys machangui* | | Rodent | | | Tanzania | 2013 | |  |  |
| MN587690 | TA531-E/TZA/2013 | Muridae | | *Lophuromys machangui* | | Rodent | | | Tanzania | 2013 | |  |  |
| MN587691 | TA532-A/TZA/2013 | Muridae | | *Lophuromys machangui* | | Rodent | | | Tanzania | 2013 | |  |  |
| MN587692 | TA532-B/TZA/2013 | Muridae | | *Lophuromys machangui* | | Rodent | | | Tanzania | 2013 | |  |  |
| MN587693 | TA532-C/TZA/2013 | Muridae | | *Lophuromys machangui* | | Rodent | | | Tanzania | 2013 | |  |  |
| MN587694 | TA532-D/TZA/2013 | Muridae | | *Lophuromys machangui* | | Rodent | | | Tanzania | 2013 | |  |  |
| MN555567 | TZ25757/TZA/2011 | Muridae | | *Acomys wilsoni* | | Rodent | | | Tanzania | 2011 | |  |  |
| **Subtotal** | **58 novel genomes** | |  | |  | |  |  | | |  | |  |
| **Total** | **188 genomes** | |  | |  | |  |  | | |  | |  |
