# Supplementary material for: Molecular detection and genomic characterization of diverse hepaciviruses in African rodents
Source: Virus Evol. 2021 Apr 12;7(1):veab036. doi: 10.1093/ve/veab036 (PMC8242229; doi:10.1093/ve/veab036)
Supplement: veab036_Supplementary_Data [file veab036_supplementary_data.zip › Table_S5_R1.docx]

**Supplementary table S5:** Cytochrome b haplotype information of all available and novel rodent hepacivirus hosts species.

| **Accession number** | **Host species** | **Country** | **Sampling year** |
| --- | --- | --- | --- |
|  |  |  |  |
| MN616976 | Lophuromys dudui | Democratic Republic of the Congo | 2010 |
| MN616984 | Graphiurus kelleni | Democratic Republic of the Congo | 2010 |
| MN616986 | Stenocephalemys albipes | Ethiopia | 2010 |
| MN616989 | Lophuromys machangui | Mozambique | 2011 |
| MN616994 | Lophuromys stanleyi | Tanzania | 2013 |
| MN616998 | Praomys jacksoni | Tanzania | 2013 |
| KY595882 | Lophuromys laticeps | Tanzania | 2013 |
| MN617006 | Mastomys natalensis | Tanzania | 2013 |
| MN617014 | Acomys wilsoni | Tanzania | 2011 |
| KY753939 | Allactaga sibirica | China | 2014 |
| KX399741 | Dipus sagitta | China | 2014 |
| JQ065613 | Meriones meridianus | China | 2014 |
| KJ612494 | Myodes glareolus | Netherlands | 2007 |
| KP190220 | Neodon clarkei | China | 2014 |
| GU126530 | Oligoryzomys nigripes | Brazil | 2012 |
| DQ385827 | Peromyscus maniculatus | USA | 2008 |
| NC_039103 | Proechimys semispinosus | Panama | 2014 |
| NC_001665 | Rattus norvegicus | USA | 2013 |
| AF533116 | Rhabdomys pumilio | South Africa | 2008 |
| NC_021478 | Rhizomys pruinosus | Vietnam | 2015 |
| NC_027283 | Spermophilus dauricus | China | 2015 |
