# Supplementary material for: Molecular detection and genomic characterization of diverse hepaciviruses in African rodents
Source: Virus Evol. 2021 Apr 12;7(1):veab036. doi: 10.1093/ve/veab036 (PMC8242229; doi:10.1093/ve/veab036)
Supplement: veab036_Supplementary_Data [file veab036_supplementary_data.zip › Table_S6_R1.docx]

**Supplementary table S6:** Specimen characteristics of all hepacivirus positive cases detected.

| **Sample** | **Species** | **Family** | **Locality** | **Country** | **Year** | **Latitude** | **Longitude** |
| --- | --- | --- | --- | --- | --- | --- | --- |
|  |  |  |  |  |  |  |  |
| 2240 | *Mastomys awashensis* | Muridae | Aroresha | Ethiopia | 2010 | 12.4166667 | 39.55 |
| ETH019 | *Stenocephalemys albipes* | Muridae | Menangasha | Ethiopia | 2012 | 8.9661 | 38.5495 |
| ETH505 | *Mastomys awashensis* | Muridae | Lake Hashenge | Ethiopia | 2012 | 12.6394 | 39.5383 |
| ETH674 | *Stenocephalemys albipes* | Muridae | Mizan Tefari | Ethiopia | 2012 | 7.06036 | 35.67192 |
| KE107 | *Gerbilliscus vicinus* | Muridae | Wamba | Kenya | 2010 | 0.9795 | 37.3272 |
| KE118 | *Graphiurus* sp. 1* | Gliridae | Marsabit | Kenya | 2010 | 2.3092 | 37.9659 |
| KE142 | *Acomys kempi* | Muridae | South Horr | Kenya | 2010 | 2.1038 | 36.8931 |
| KE153 | *Acomys kempi* | Muridae | South Horr | Kenya | 2010 | 2.0973 | 36.8992 |
| KE389 | *Hylomyscus stella* | Muridae | Kakamega | Kenya | 2010 | 0.2382 | 34.8647 |
| KE645 | *Gerbilliscus vicinus* | Muridae | Kapiti plains | Kenya | 2011 | -1.4842 | 37.0552 |
| KE839 | *Lophuromys zena* | Muridae | Nyahururu forest | Kenya | 2011 | 0.0449 | 36.3734 |
| KE840 | *Lophuromys zena* | Muridae | Nyahururu forest | Kenya | 2011 | 0.0449 | 36.3734 |
| TA085 | *Lophuromys stanleyi* | Muridae | Minziro FR | Tanzania | 2013 | -1.031 | 31.572 |
| TA094 | *Praomys jacksoni* | Muridae | Minziro FR | Tanzania | 2013 | -1.031 | 31.572 |
| TA100 | *Lophuromys stanleyi* | Muridae | Minziro FR | Tanzania | 2013 | -1.031 | 31.572 |
| TA106 | *Lophuromys stanleyi* | Muridae | Minziro FR | Tanzania | 2013 | -1.031 | 31.572 |
| TA109 | *Lophuromys stanleyi* | Muridae | Minziro FR | Tanzania | 2013 | -1.031 | 31.572 |
| TA132 | *Praomys jacksoni* | Muridae | Minziro FR | Tanzania | 2013 | -1.031 | 31.572 |
| TA142 | *Praomys jacksoni* | Muridae | Minziro FR | Tanzania | 2013 | -1.031 | 31.572 |
| TA151 | *Praomys jacksoni* | Muridae | Minziro FR | Tanzania | 2013 | -1.031 | 31.572 |
| TA152 | *Praomys jacksoni* | Muridae | Minziro FR | Tanzania | 2013 | -1.031 | 31.572 |
| TA156 | *Praomys jacksoni* | Muridae | Minziro FR | Tanzania | 2013 | -1.031 | 31.572 |
| TA166 | *Glauconycteris atra* | Vespertilionidae | Minziro FR | Tanzania | 2013 | -1.031 | 31.572 |
| TA168 | *Glauconycteris atra* | Vespertilionidae | Minziro FR | Tanzania | 2013 | -1.031 | 31.572 |
| TA275 | *Lophuromys laticeps* | Muridae | Bitale (North of Kigoma) | Tanzania | 2013 | -4.731659 | 29.706526 |
| TA289 | *Lophuromys laticeps* | Muridae | Bitale (North of Kigoma) | Tanzania | 2013 | -4.731659 | 29.706526 |
| TA293 | *Lophuromys laticeps* | Muridae | Bitale (North of Kigoma) | Tanzania | 2013 | -4.731659 | 29.706526 |
| TA338 | *Mastomys natalensis* | Muridae | Makongoro | Tanzania | 2013 | -6.451433 | 31.115017 |
| TA497 | *Lophuromys machangui* | Muridae | Mt Ngozi (Poroto Range) | Tanzania | 2013 | -9.04075 | 33.5732 |
| TA498 | *Lophuromys machangui* | Muridae | Mt Ngozi (Poroto Range) | Tanzania | 2013 | -9.04075 | 33.5732 |
| TA502 | *Lophuromys machangui* | Muridae | Mt Ngozi (Poroto Range) | Tanzania | 2013 | -9.04075 | 33.5732 |
| TA503 | *Lophuromys machangui* | Muridae | Mt Ngozi (Poroto Range) | Tanzania | 2013 | -9.04075 | 33.5732 |
| TA504 | *Lophuromys machangui* | Muridae | Mt Ngozi (Poroto Range) | Tanzania | 2013 | -9.04075 | 33.5732 |
| TA527 | *Lophuromys machangui* | Muridae | Mt Ngozi (Poroto Range) | Tanzania | 2013 | -9.04075 | 33.5732 |
| TA528 | *Lophuromys machangui* | Muridae | Mt Ngozi (Poroto Range) | Tanzania | 2013 | -9.04075 | 33.5732 |
| TA529 | *Lophuromys machangui* | Muridae | Mt Ngozi (Poroto Range) | Tanzania | 2013 | -9.04075 | 33.5732 |
| TA530 | *Lophuromys machangui* | Muridae | Mt Ngozi (Poroto Range) | Tanzania | 2013 | -9.04075 | 33.5732 |
| TA531 | *Lophuromys machangui* | Muridae | Mt Ngozi (Poroto Range) | Tanzania | 2013 | -9.04075 | 33.5732 |
| TA532 | *Lophuromys machangui* | Muridae | Mt Ngozi (Poroto Range) | Tanzania | 2013 | -9.04075 | 33.5732 |
| TA533 | *Lophuromys machangui* | Muridae | Mt Ngozi (Poroto Range) | Tanzania | 2013 | -9.04075 | 33.5732 |
| VM067 | *Saccostomus campestris* | Nesomyidae | Chipata - Mamarula Camp | Zambia | 2010 | -13.5823 | 32.6099 |
| CRT112 | *Lophuromys dudui* | Muridae | Yaikela Ligne Pitfall Kaswera | DRC | 2010 | 0.81361 | 24.27913 |
| CRT125 | *Lophuromys dudui* | Muridae | Yaikela Ligne 2 | DRC | 2010 | 0.82099 | 24.27678 |
| CRT352 | *Lophuromys dudui* | Muridae | Bomane Ligne 1 KGB | DRC | 2010 | 1.2 | 23.7 |
| CRT353 | *Hybomys* sp. | Muridae | Bomane Ligne 2 KGB | DRC | 2010 | 1.2 | 23.7 |
| CRT382 | *Lophuromys dudui* | Muridae | Bomane Ligne 2 KGB | DRC | 2010 | 1.2 | 23.7 |
| CRT471 | *Lophuromys dudui* | Muridae | Bomane Champ Nic2 | DRC | 2010 | 1.27374 | 23.72831 |
| CRT490 | *Lophuromys dudui* | Muridae | Bomane Champ Nic2 | DRC | 2010 | 1.27374 | 23.72831 |
| CRT502 | *Lemniscomys striatus* | Muridae | Bomane Champ Nic2 | DRC | 2010 | 1.27374 | 23.72831 |
| CRT518 | *Praomys* sp. | Muridae | Bomane Ligne 1 Dudu ile | DRC | 2010 | 1.26534 | 23.74014 |
| CRT548 | *Praomys* sp. | Muridae | Bomane Ligne 1 Dudu ile | DRC | 2010 | 1.26534 | 23.74014 |
| CRT551 | *Lemniscomys striatus* | Muridae |  | DRC | 2010 |  |  |
| CRT558 | *Praomys jacksoni* | Muridae | Bomane Ligne 2 Dudu ile | DRC | 2010 | 1.26573 | 23.74016 |
| CRT64 | *Lophuromys dudui* | Muridae | Yaikela Ligne 2 | DRC | 2010 | 0.82099 | 24.27678 |
| CRT682 | *Graphiurus* sp. 2* | Gliridae | Lieki paysans RD Dudu | DRC | 2010 | 0.67661 | 24.22865 |
| CRT74 | *Lophuromys dudui* | Muridae | Yaikela Ligne 2 | DRC | 2010 | 0.82099 | 24.27678 |
| EPU7 | *Lophuromys* sp. | Muridae | Mayali mingi | DRC | 2012 | 1.40646 | 28.56027 |
| KRT-210 | *Acomys* sp. | Muridae | Karatu | Tanzania | 2010 | -3.3 | 35.6 |
| TE5836 | *Lophuromys kilonzoi* | Muridae | Magamba | Tanzania | 2006 | -4.75 | 38.29 |
| MOZ002 | *Micaelamys namaquensis* | Muridae | Chimanimani | Mozambique | 2011 | -19.7105 | 33.0069 |
| MOZ068 | *Lophuromys machangui* | Muridae | Mt Mabu | Mozambique | 2011 | -16.3057 | 36.4241 |
| MOZ091 | *Lophuromys machangui* | Muridae | Mt Mabu | Mozambique | 2011 | -16.3057 | 36.4241 |
| MOZ094 | *Lophuromys machangui* | Muridae | Mt Mabu | Mozambique | 2011 | -16.3057 | 36.4241 |
| MOZ133 | *Lophuromys machangui* | Muridae | Mt Mabu | Mozambique | 2011 | -16.3086 | 36.4245 |
| MOZ134 | *Lophuromys machangui* | Muridae | Mt Mabu | Mozambique | 2011 | -16.3086 | 36.4245 |
| MOZ135 | *Lophuromys machangui* | Muridae | Mt Mabu | Mozambique | 2011 | -16.3086 | 36.4245 |
| MOZ241 | *Lophuromys machangui* | Muridae | Gile NP | Mozambique | 2011 | -16.7018 | 38.7984 |
| MOZ295 | *Lophuromys machangui* | Muridae | Mt Jesi | Mozambique | 2011 | -12.8675 | 35.185 |
| MOZ329 | *Lophuromys machangui* | Muridae | Gurue | Mozambique | 2011 | -15.7366 | 37.2063 |
| T8_345 | *Tachyoryctes splendens* | Spalacidae | Kilimanjaro-Tarakea | Tanzania | 2008 | -3.0569 | 37.5321 |
| T8_359 | *Heliophobius argenteocinereus* | Bathyergidae | Handei forest | Tanzania | 2008 | -5.02948 | 38.60332 |
| T8_510 | *Gerbilliscus vicinus* | Muridae | Shinyanga-Lubaga | Tanzania | 2008 | -3.6375 | 33.41723 |
| TZ20496 | *Lophuromys kilonzoi* | Muridae | Gologolo | Tanzania | 2007 | -4.8 | 38.26666667 |
| TZ20565 | *Rattus rattus* | Muridae | Emao | Tanzania | 2007 | -4.63 | 38.27 |
| TZ20612 | *Lophuromys kilonzoi* | Muridae | Kiranga | Tanzania | 2007 | -4.58 | 38.27 |
| TZ20620 | *Lophuromys kilonzoi* | Muridae | Kiranga | Tanzania | 2007 | -4.58 | 38.27 |
| TZ21205 | *Grammomys* sp. | Muridae | Magamba | Tanzania | 2009 | -4.75 | 38.28333333 |
| TZ21837 | *Lophuromys kilonzoi* | Muridae | Lushoto | Tanzania | 2009 | -4.8 | 38.3 |
| TZ25717 | *Acomys wilsoni* | Muridae | Mbwewe_site 1 | Tanzania | 2011 | -5.99493 | 38.23189 |
| TZ25757 | *Acomys wilsoni* | Muridae | Mihuga_site 2 | Tanzania | 2011 | -6.20882 | 38.53539 |

* The current nomenclature does not reliably delineate the genetic lineages in the *Graphiurus* genus. Based on the experts’ opinion that co-authored this manuscript, *Graphiurus* individuals from Kenya are genetically different from those in the DRC. Although we cannot update the rodent species taxonomy within the scope of this manuscript, we will correspond *Graphiurus* sp. 1 to the Kenyan specimen and *Graphiurus* sp. 2 to the specimen originating from the DRC.
