# Supplementary material for: Molecular detection and genomic characterization of diverse hepaciviruses in African rodents
Source: Virus Evol. 2021 Apr 12;7(1):veab036. doi: 10.1093/ve/veab036 (PMC8242229; doi:10.1093/ve/veab036)
Supplement: veab036_Supplementary_Data [file veab036_supplementary_data.zip › Table_S7a_R1_PDF.pdf]

| | 1 | 2 | 3 | 4 | 5 | 6 | 7 | 8 | 9 | 10 | 11 | 12 | 13 | 14 | 15 | 16 | 17 | 18 | 19 | 20 | 21 | 22 | 23 | 24 | 25 | 26 | 27 | 28 | 29 | 30 | 31 | 32 | 33 | 34 | 35 | 36 | 37 | 38 | 39 | 40 | 41 | 42 | 43 | 44 | 45 | 46 | 47 | 48 | 49 | 50 | 51 | 52 | 53 | 54 | 55 | 56 | 57 | 58 | 59 |
|---|---|---|---|---|---|---|---|---|---|---|---|---|---|---|---|---|---|---|---|---|---|---|---|---|---|---|---|---|---|---|---|---|---|---|---|---|---|---|---|---|---|---|---|---|---|---|---|---|---|---|---|---|---|---|---|---|---|---|---|
| 1 MG599990_fish/rept/turtle_Gymnothorax_reticularis_China |
| 2 MG334001_fish/rept/turtle_Trachemys_scripta_USA_2008 | 0.629 |
| 3 MK737640_duck_Anas_platyrhynchos_China_2018 | 0.605 | 0.524 |
| 4 MN133813_mosquito_Culex_annulirostris_Australia_2018 | 0.626 | 0.58 | 0.531 |
| 5 MN062427_eagle_Haliaeetus_leucocephalus_USA_2002 | 0.598 | 0.584 | 0.517 | 0.451 |
| 6 MG599993_fish/rept/turtle_Protopterus_annectens_Nigeria | 0.622 | 0.524 | 0.563 | 0.601 | 0.556 |
| 7 MG599988_fish/rept/turtle_Goniurosaurus_luii_China | 0.605 | 0.605 | 0.598 | 0.587 | 0.573 | 0.563 |
| 8 MG599987_fish/rept/turtle_Teratoscincus_roborowskii_China | 0.636 | 0.626 | 0.584 | 0.626 | 0.619 | 0.612 | 0.476 |
| 9 MG599989_fish/rept/turtle_Hemidactylus_bowringii_China | 0.612 | 0.594 | 0.573 | 0.601 | 0.549 | 0.584 | 0.455 | 0.458 |
| 10 MG599991_fish/rept/turtle_Rhinobatos_hynnicephalus_China | 0.591 | 0.507 | 0.528 | 0.545 | 0.521 | 0.472 | 0.601 | 0.608 | 0.563 |
| 11 MG599992_fish/rept/turtle_Urolophus_aurantiacus_China | 0.647 | 0.524 | 0.538 | 0.577 | 0.549 | 0.483 | 0.594 | 0.612 | 0.577 | 0.437 |
| 12 KP641127_cattle_Bos_taurus_Germany_2014 | 0.587 | 0.517 | 0.503 | 0.535 | 0.542 | 0.521 | 0.563 | 0.566 | 0.524 | 0.503 | 0.517 |
| 13 KC551800_primate_Colobus_guereza_Uganda_2010 | 0.605 | 0.545 | 0.521 | 0.566 | 0.524 | 0.524 | 0.566 | 0.552 | 0.563 | 0.5 | 0.556 | 0.42 |
| 14 MH370348_rodent_Oligoryzomys_nigripes_Brazil_2012 | 0.608 | 0.563 | 0.556 | 0.584 | 0.563 | 0.514 | 0.601 | 0.573 | 0.57 | 0.542 | 0.545 | 0.455 | 0.451 |
| 15 KC411806_rodent_Rhabdomys_pumilio_South_Africa_2008 | 0.629 | 0.556 | 0.51 | 0.584 | 0.563 | 0.521 | 0.598 | 0.57 | 0.563 | 0.531 | 0.549 | 0.49 | 0.472 | 0.378 |
| 16 KY370092_rodent_Meriones_meridianus_China_2014 | 0.612 | 0.556 | 0.521 | 0.563 | 0.542 | 0.514 | 0.556 | 0.57 | 0.591 | 0.517 | 0.545 | 0.465 | 0.469 | 0.381 | 0.329 |
| 17 AF179612_primate_Saguinus_mystax_South_America_1995 | 0.584 | 0.552 | 0.549 | 0.545 | 0.507 | 0.545 | 0.556 | 0.57 | 0.531 | 0.503 | 0.514 | 0.472 | 0.465 | 0.517 | 0.549 | 0.524 |
| 18 MF775364_shrew_Suncus_murinus_China_2015 | 0.664 | 0.566 | 0.559 | 0.577 | 0.57 | 0.556 | 0.573 | 0.587 | 0.58 | 0.549 | 0.542 | 0.479 | 0.524 | 0.531 | 0.531 | 0.566 | 0.521 |
| 19 KC796077_bat_Hipposideros_vittatus_Kenya_2010 | 0.612 | 0.542 | 0.483 | 0.517 | 0.493 | 0.507 | 0.545 | 0.549 | 0.521 | 0.476 | 0.514 | 0.392 | 0.378 | 0.451 | 0.483 | 0.455 | 0.413 | 0.514 |
| 20 MN635449_possum_Trichosurus_vulpecula_Sydney | 0.601 | 0.573 | 0.531 | 0.577 | 0.556 | 0.556 | 0.594 | 0.587 | 0.57 | 0.514 | 0.542 | 0.441 | 0.476 | 0.528 | 0.524 | 0.545 | 0.444 | 0.517 | 0.451 |
| 21 KJ950939_rodent_Rattus_norvegicus_USA_2012 | 0.594 | 0.559 | 0.528 | 0.573 | 0.556 | 0.535 | 0.577 | 0.573 | 0.559 | 0.531 | 0.542 | 0.448 | 0.448 | 0.517 | 0.521 | 0.517 | 0.448 | 0.5 | 0.413 | 0.416 |
| 22 MH824542_sifaka_Propithecus_diadema_Madagascar_2013 | 0.598 | 0.535 | 0.528 | 0.566 | 0.507 | 0.514 | 0.573 | 0.552 | 0.549 | 0.514 | 0.514 | 0.444 | 0.43 | 0.493 | 0.514 | 0.524 | 0.43 | 0.542 | 0.406 | 0.402 | 0.448 |
| 23 MG211815_rodent_Spermophilus_dauricus_China_2015 | 0.629 | 0.563 | 0.51 | 0.577 | 0.528 | 0.507 | 0.573 | 0.566 | 0.552 | 0.507 | 0.524 | 0.434 | 0.437 | 0.469 | 0.51 | 0.479 | 0.451 | 0.497 | 0.388 | 0.43 | 0.371 | 0.364 |
| 24 MN564789_CRT682_rodent_Graphiurus_cf._kelleni_DRC_2010 | 0.633 | 0.573 | 0.538 | 0.591 | 0.531 | 0.545 | 0.605 | 0.584 | 0.577 | 0.521 | 0.535 | 0.462 | 0.479 | 0.479 | 0.535 | 0.497 | 0.497 | 0.528 | 0.451 | 0.465 | 0.43 | 0.42 | 0.388 |
| 25 MN587695_TA085_rodent_Lophuromys_stanleyi_Tanzania_2013 | 0.619 | 0.545 | 0.503 | 0.58 | 0.566 | 0.483 | 0.58 | 0.552 | 0.545 | 0.497 | 0.517 | 0.476 | 0.444 | 0.465 | 0.472 | 0.486 | 0.476 | 0.472 | 0.448 | 0.469 | 0.434 | 0.458 | 0.367 | 0.409 |
| 26 MN587668_MOZ133_rodent_Lophuromys_machangui_Mozambique_2011 | 0.626 | 0.556 | 0.514 | 0.58 | 0.573 | 0.49 | 0.58 | 0.563 | 0.542 | 0.528 | 0.521 | 0.479 | 0.437 | 0.465 | 0.486 | 0.503 | 0.497 | 0.483 | 0.444 | 0.472 | 0.441 | 0.469 | 0.392 | 0.423 | 0.101 |
| 27 KJ950938_rodent_Rattus_norvegicus_USA_2013 | 0.619 | 0.559 | 0.542 | 0.566 | 0.538 | 0.531 | 0.577 | 0.58 | 0.535 | 0.524 | 0.545 | 0.448 | 0.416 | 0.476 | 0.521 | 0.51 | 0.444 | 0.51 | 0.402 | 0.413 | 0.385 | 0.374 | 0.353 | 0.434 | 0.409 | 0.416 |
| 28 KY370095_rodent_Dipus_sagitta_China_2014 | 0.629 | 0.584 | 0.535 | 0.587 | 0.556 | 0.538 | 0.584 | 0.587 | 0.58 | 0.514 | 0.538 | 0.458 | 0.409 | 0.51 | 0.486 | 0.465 | 0.479 | 0.51 | 0.441 | 0.444 | 0.395 | 0.42 | 0.388 | 0.423 | 0.399 | 0.427 | 0.381 |
| 29 MN555567_TZ25757_rodent_Acomys_wilsoni_Tanzania_2011 | 0.636 | 0.577 | 0.531 | 0.598 | 0.545 | 0.535 | 0.601 | 0.584 | 0.563 | 0.51 | 0.549 | 0.451 | 0.462 | 0.514 | 0.51 | 0.479 | 0.469 | 0.521 | 0.462 | 0.462 | 0.416 | 0.413 | 0.381 | 0.409 | 0.448 | 0.465 | 0.378 | 0.357 |
| 30 MG822666_rodent_Proechimys_semispinosus_Panama_2014 | 0.664 | 0.605 | 0.559 | 0.615 | 0.531 | 0.573 | 0.605 | 0.605 | 0.566 | 0.559 | 0.552 | 0.493 | 0.476 | 0.497 | 0.535 | 0.521 | 0.5 | 0.517 | 0.479 | 0.476 | 0.42 | 0.448 | 0.43 | 0.406 | 0.469 | 0.472 | 0.381 | 0.395 | 0.322 |
| 31 MN564790_ETH674_rodent_Stenocephalemys_albipes_Ethiopia_2012 | 0.643 | 0.584 | 0.57 | 0.598 | 0.542 | 0.552 | 0.619 | 0.601 | 0.584 | 0.542 | 0.535 | 0.493 | 0.462 | 0.503 | 0.514 | 0.49 | 0.49 | 0.538 | 0.451 | 0.469 | 0.385 | 0.42 | 0.402 | 0.381 | 0.444 | 0.441 | 0.371 | 0.357 | 0.273 | 0.318 |
| 32 MG600413_rodent_Rhizomys_pruinosus_Vietnam_2015 | 0.643 | 0.591 | 0.549 | 0.601 | 0.566 | 0.559 | 0.598 | 0.573 | 0.563 | 0.559 | 0.552 | 0.476 | 0.472 | 0.493 | 0.507 | 0.49 | 0.476 | 0.535 | 0.458 | 0.472 | 0.395 | 0.427 | 0.378 | 0.413 | 0.43 | 0.448 | 0.364 | 0.36 | 0.287 | 0.322 | 0.311 |
| 33 KC815310_rodent_Peromyscus_maniculatus_USA_2008 | 0.633 | 0.587 | 0.542 | 0.598 | 0.566 | 0.535 | 0.573 | 0.573 | 0.538 | 0.538 | 0.514 | 0.458 | 0.465 | 0.5 | 0.51 | 0.503 | 0.462 | 0.521 | 0.441 | 0.451 | 0.381 | 0.406 | 0.367 | 0.395 | 0.43 | 0.441 | 0.364 | 0.346 | 0.255 | 0.35 | 0.266 | 0.287 |
| 34 KY370094_rodent_Neodon_clarkei_China_2014 | 0.65 | 0.556 | 0.524 | 0.594 | 0.521 | 0.531 | 0.605 | 0.594 | 0.566 | 0.51 | 0.514 | 0.455 | 0.437 | 0.49 | 0.497 | 0.493 | 0.444 | 0.528 | 0.434 | 0.434 | 0.395 | 0.406 | 0.416 | 0.402 | 0.437 | 0.458 | 0.371 | 0.336 | 0.262 | 0.301 | 0.287 | 0.301 | 0.266 |
| 35 KC411784_rodent_Myodes_glareolus_Netherlands_2007 | 0.633 | 0.559 | 0.517 | 0.612 | 0.531 | 0.549 | 0.615 | 0.612 | 0.58 | 0.507 | 0.531 | 0.465 | 0.462 | 0.503 | 0.507 | 0.514 | 0.486 | 0.542 | 0.455 | 0.43 | 0.42 | 0.402 | 0.413 | 0.406 | 0.441 | 0.469 | 0.378 | 0.374 | 0.259 | 0.322 | 0.304 | 0.325 | 0.269 | 0.22 |
| 36 KC796074_bat_Hipposideros_vittatus_Kenya_2011 | 0.629 | 0.49 | 0.517 | 0.545 | 0.563 | 0.503 | 0.587 | 0.563 | 0.542 | 0.483 | 0.503 | 0.486 | 0.472 | 0.514 | 0.497 | 0.507 | 0.507 | 0.528 | 0.462 | 0.538 | 0.514 | 0.517 | 0.49 | 0.51 | 0.472 | 0.472 | 0.49 | 0.503 | 0.531 | 0.535 | 0.524 | 0.517 | 0.524 | 0.507 | 0.524 |
| 37 KC796078_bat_Otomops_martiensseni_Kenya_2011 | 0.615 | 0.497 | 0.493 | 0.545 | 0.573 | 0.493 | 0.577 | 0.528 | 0.528 | 0.507 | 0.514 | 0.469 | 0.507 | 0.542 | 0.514 | 0.524 | 0.517 | 0.51 | 0.479 | 0.545 | 0.472 | 0.49 | 0.483 | 0.503 | 0.462 | 0.469 | 0.486 | 0.486 | 0.476 | 0.514 | 0.5 | 0.486 | 0.486 | 0.483 | 0.497 | 0.353 |
| 38 KP325401_horse_Equus_ferus_USA_2011 | 0.587 | 0.497 | 0.503 | 0.556 | 0.58 | 0.514 | 0.573 | 0.559 | 0.528 | 0.483 | 0.507 | 0.483 | 0.479 | 0.503 | 0.503 | 0.531 | 0.483 | 0.514 | 0.448 | 0.524 | 0.465 | 0.486 | 0.476 | 0.528 | 0.469 | 0.458 | 0.493 | 0.483 | 0.503 | 0.524 | 0.524 | 0.51 | 0.497 | 0.486 | 0.503 | 0.357 | 0.364 |
| 39 EF108306_human_Homo_sapiens | 0.619 | 0.514 | 0.542 | 0.535 | 0.556 | 0.472 | 0.58 | 0.577 | 0.552 | 0.486 | 0.556 | 0.493 | 0.455 | 0.552 | 0.556 | 0.552 | 0.476 | 0.545 | 0.451 | 0.535 | 0.5 | 0.476 | 0.5 | 0.566 | 0.486 | 0.497 | 0.514 | 0.514 | 0.517 | 0.549 | 0.552 | 0.549 | 0.528 | 0.5 | 0.535 | 0.416 | 0.42 | 0.336 |
| 40 NC_009823_human_Homo_sapiens | 0.622 | 0.517 | 0.538 | 0.538 | 0.556 | 0.486 | 0.573 | 0.58 | 0.552 | 0.493 | 0.559 | 0.476 | 0.472 | 0.528 | 0.514 | 0.542 | 0.479 | 0.542 | 0.448 | 0.524 | 0.514 | 0.49 | 0.493 | 0.563 | 0.493 | 0.507 | 0.514 | 0.521 | 0.521 | 0.552 | 0.549 | 0.514 | 0.531 | 0.528 | 0.517 | 0.406 | 0.427 | 0.329 | 0.136 |
| 41 NC_009824_human_Homo_sapiens | 0.615 | 0.51 | 0.521 | 0.531 | 0.552 | 0.49 | 0.58 | 0.584 | 0.563 | 0.49 | 0.549 | 0.507 | 0.465 | 0.524 | 0.524 | 0.542 | 0.472 | 0.542 | 0.448 | 0.549 | 0.517 | 0.493 | 0.5 | 0.556 | 0.469 | 0.486 | 0.517 | 0.507 | 0.507 | 0.559 | 0.545 | 0.524 | 0.528 | 0.528 | 0.521 | 0.402 | 0.413 | 0.308 | 0.164 | 0.154 |
| 42 NC_009826_human_Homo_sapiens | 0.633 | 0.517 | 0.528 | 0.545 | 0.556 | 0.507 | 0.58 | 0.587 | 0.58 | 0.507 | 0.545 | 0.5 | 0.462 | 0.535 | 0.524 | 0.528 | 0.486 | 0.538 | 0.455 | 0.545 | 0.514 | 0.486 | 0.493 | 0.57 | 0.497 | 0.503 | 0.521 | 0.514 | 0.51 | 0.542 | 0.531 | 0.514 | 0.521 | 0.5 | 0.507 | 0.406 | 0.448 | 0.343 | 0.143 | 0.143 | 0.154 |
| 43 NC_009827_human_Homo_sapiens | 0.626 | 0.535 | 0.535 | 0.552 | 0.552 | 0.483 | 0.594 | 0.566 | 0.556 | 0.503 | 0.559 | 0.5 | 0.476 | 0.531 | 0.514 | 0.521 | 0.497 | 0.535 | 0.458 | 0.528 | 0.503 | 0.479 | 0.493 | 0.542 | 0.483 | 0.483 | 0.517 | 0.503 | 0.503 | 0.549 | 0.521 | 0.517 | 0.514 | 0.507 | 0.517 | 0.434 | 0.416 | 0.332 | 0.154 | 0.157 | 0.189 | 0.147 |
| 44 NC_009825_human_Homo_sapiens | 0.622 | 0.51 | 0.524 | 0.556 | 0.563 | 0.497 | 0.584 | 0.577 | 0.552 | 0.507 | 0.542 | 0.49 | 0.472 | 0.517 | 0.524 | 0.524 | 0.479 | 0.542 | 0.451 | 0.535 | 0.5 | 0.483 | 0.486 | 0.556 | 0.479 | 0.479 | 0.507 | 0.507 | 0.497 | 0.524 | 0.521 | 0.503 | 0.5 | 0.507 | 0.497 | 0.413 | 0.437 | 0.329 | 0.143 | 0.154 | 0.161 | 0.143 | 0.133 |
| 45 M62321_HCV_reference | 0.626 | 0.521 | 0.524 | 0.542 | 0.556 | 0.472 | 0.58 | 0.566 | 0.549 | 0.479 | 0.528 | 0.493 | 0.451 | 0.51 | 0.497 | 0.51 | 0.476 | 0.535 | 0.423 | 0.521 | 0.493 | 0.462 | 0.476 | 0.542 | 0.469 | 0.486 | 0.493 | 0.5 | 0.493 | 0.521 | 0.507 | 0.507 | 0.497 | 0.486 | 0.5 | 0.378 | 0.395 | 0.308 | 0.136 | 0.143 | 0.171 | 0.14 | 0.122 | 0.129 |
| 46 MG599997_fish/rept/turtle_Chimaera_sp_China | 0.594 | 0.507 | 0.497 | 0.57 | 0.559 | 0.483 | 0.608 | 0.615 | 0.577 | 0.43 | 0.448 | 0.483 | 0.528 | 0.517 | 0.528 | 0.514 | 0.514 | 0.559 | 0.5 | 0.517 | 0.524 | 0.49 | 0.514 | 0.5 | 0.517 | 0.538 | 0.517 | 0.528 | 0.514 | 0.538 | 0.521 | 0.528 | 0.507 | 0.503 | 0.531 | 0.493 | 0.5 | 0.497 | 0.51 | 0.493 | 0.503 | 0.493 | 0.5 | 0.5 | 0.483 |
| 47 MG599996_fish/rept/turtle_Chimaera_sp_China | 0.619 | 0.51 | 0.497 | 0.556 | 0.545 | 0.493 | 0.587 | 0.619 | 0.58 | 0.451 | 0.476 | 0.455 | 0.531 | 0.497 | 0.486 | 0.51 | 0.521 | 0.535 | 0.462 | 0.528 | 0.521 | 0.476 | 0.503 | 0.521 | 0.542 | 0.538 | 0.538 | 0.531 | 0.51 | 0.538 | 0.542 | 0.521 | 0.528 | 0.51 | 0.5 | 0.497 | 0.507 | 0.503 | 0.514 | 0.5 | 0.507 | 0.503 | 0.514 | 0.514 | 0.507 | 0.371 |
| 48 MG599995_fish/rept/turtle_Squalus_brevirostris_China | 0.594 | 0.535 | 0.5 | 0.573 | 0.531 | 0.503 | 0.598 | 0.619 | 0.549 | 0.476 | 0.458 | 0.497 | 0.57 | 0.563 | 0.552 | 0.545 | 0.51 | 0.556 | 0.503 | 0.531 | 0.57 | 0.521 | 0.549 | 0.538 | 0.545 | 0.563 | 0.531 | 0.58 | 0.521 | 0.57 | 0.573 | 0.549 | 0.521 | 0.538 | 0.531 | 0.49 | 0.497 | 0.507 | 0.535 | 0.524 | 0.524 | 0.531 | 0.521 | 0.535 | 0.521 | 0.448 | 0.437 |
| 49 MG599994_fish/rept/turtle_Mustelus_manazo_China | 0.629 | 0.524 | 0.507 | 0.57 | 0.566 | 0.514 | 0.594 | 0.615 | 0.598 | 0.43 | 0.479 | 0.531 | 0.521 | 0.566 | 0.559 | 0.552 | 0.528 | 0.535 | 0.521 | 0.514 | 0.545 | 0.507 | 0.517 | 0.559 | 0.531 | 0.545 | 0.535 | 0.531 | 0.535 | 0.58 | 0.559 | 0.57 | 0.535 | 0.528 | 0.542 | 0.483 | 0.479 | 0.476 | 0.507 | 0.524 | 0.51 | 0.531 | 0.528 | 0.521 | 0.497 | 0.413 | 0.437 | 0.451 |
| 50 MG599999_fish/rept/turtle_Pelodiscus_sinensis_China | 0.619 | 0.493 | 0.5 | 0.535 | 0.531 | 0.503 | 0.591 | 0.591 | 0.584 | 0.451 | 0.493 | 0.49 | 0.514 | 0.559 | 0.521 | 0.51 | 0.517 | 0.552 | 0.493 | 0.542 | 0.535 | 0.503 | 0.507 | 0.542 | 0.514 | 0.521 | 0.521 | 0.514 | 0.524 | 0.545 | 0.545 | 0.556 | 0.542 | 0.51 | 0.507 | 0.458 | 0.458 | 0.469 | 0.497 | 0.503 | 0.486 | 0.5 | 0.479 | 0.507 | 0.479 | 0.476 | 0.448 | 0.483 | 0.462 |
| 51 MG600000_fish/rept/turtle_Mauremys_megalocephala_China | 0.591 | 0.524 | 0.497 | 0.57 | 0.524 | 0.497 | 0.563 | 0.601 | 0.563 | 0.507 | 0.514 | 0.486 | 0.5 | 0.545 | 0.559 | 0.535 | 0.497 | 0.573 | 0.465 | 0.538 | 0.545 | 0.5 | 0.462 | 0.538 | 0.483 | 0.507 | 0.486 | 0.514 | 0.528 | 0.556 | 0.552 | 0.549 | 0.521 | 0.51 | 0.51 | 0.469 | 0.486 | 0.497 | 0.465 | 0.476 | 0.441 | 0.469 | 0.469 | 0.472 | 0.469 | 0.479 | 0.476 | 0.483 | 0.479 | 0.308 |
| 52 KY370091_rodent_Allactaga_sibirica_China_2014 | 0.584 | 0.479 | 0.486 | 0.528 | 0.545 | 0.514 | 0.577 | 0.556 | 0.531 | 0.514 | 0.493 | 0.483 | 0.49 | 0.535 | 0.542 | 0.514 | 0.535 | 0.545 | 0.465 | 0.528 | 0.507 | 0.503 | 0.507 | 0.531 | 0.51 | 0.51 | 0.472 | 0.514 | 0.517 | 0.563 | 0.535 | 0.521 | 0.517 | 0.49 | 0.524 | 0.458 | 0.441 | 0.458 | 0.469 | 0.462 | 0.486 | 0.465 | 0.455 | 0.465 | 0.462 | 0.462 | 0.476 | 0.486 | 0.462 | 0.458 | 0.465 |
| 53 KC411777_rodent_Myodes_glareolus_Germany_2010 | 0.577 | 0.476 | 0.493 | 0.549 | 0.51 | 0.493 | 0.573 | 0.57 | 0.549 | 0.462 | 0.493 | 0.448 | 0.493 | 0.503 | 0.497 | 0.493 | 0.469 | 0.552 | 0.448 | 0.517 | 0.51 | 0.451 | 0.479 | 0.462 | 0.469 | 0.5 | 0.493 | 0.5 | 0.483 | 0.531 | 0.5 | 0.507 | 0.517 | 0.472 | 0.497 | 0.43 | 0.416 | 0.448 | 0.423 | 0.43 | 0.423 | 0.437 | 0.434 | 0.437 | 0.423 | 0.434 | 0.455 | 0.5 | 0.486 | 0.42 | 0.406 | 0.388 |
| 54 MN564792_TA132_rodent_Praomys_jacksoni_Tanzania_2013 | 0.654 | 0.472 | 0.5 | 0.552 | 0.556 | 0.486 | 0.584 | 0.612 | 0.58 | 0.472 | 0.538 | 0.486 | 0.5 | 0.524 | 0.521 | 0.503 | 0.514 | 0.545 | 0.493 | 0.552 | 0.552 | 0.507 | 0.486 | 0.545 | 0.497 | 0.517 | 0.517 | 0.514 | 0.524 | 0.57 | 0.552 | 0.542 | 0.542 | 0.521 | 0.531 | 0.448 | 0.472 | 0.479 | 0.472 | 0.476 | 0.465 | 0.462 | 0.458 | 0.465 | 0.458 | 0.483 | 0.479 | 0.542 | 0.472 | 0.444 | 0.458 | 0.451 | 0.308 |
| 55 MN587672_TA275_rodent_Lophuromys_laticeps_Tanzania_2013 | 0.643 | 0.49 | 0.528 | 0.587 | 0.559 | 0.5 | 0.584 | 0.605 | 0.556 | 0.49 | 0.552 | 0.49 | 0.51 | 0.549 | 0.563 | 0.549 | 0.507 | 0.584 | 0.497 | 0.531 | 0.545 | 0.493 | 0.483 | 0.559 | 0.531 | 0.535 | 0.51 | 0.517 | 0.524 | 0.556 | 0.563 | 0.542 | 0.556 | 0.493 | 0.531 | 0.486 | 0.472 | 0.486 | 0.465 | 0.476 | 0.462 | 0.462 | 0.441 | 0.469 | 0.472 | 0.5 | 0.5 | 0.535 | 0.5 | 0.462 | 0.434 | 0.455 | 0.318 | 0.217 |
| 56 MN587655_CRT352-B_rodent_Lophuromys_dudui_DRC_2010 | 0.629 | 0.486 | 0.535 | 0.58 | 0.556 | 0.51 | 0.591 | 0.619 | 0.552 | 0.503 | 0.552 | 0.507 | 0.51 | 0.545 | 0.552 | 0.552 | 0.517 | 0.57 | 0.507 | 0.542 | 0.556 | 0.49 | 0.486 | 0.559 | 0.528 | 0.538 | 0.517 | 0.521 | 0.535 | 0.566 | 0.559 | 0.556 | 0.559 | 0.521 | 0.556 | 0.469 | 0.479 | 0.479 | 0.462 | 0.469 | 0.465 | 0.455 | 0.434 | 0.465 | 0.465 | 0.486 | 0.5 | 0.528 | 0.493 | 0.462 | 0.43 | 0.441 | 0.315 | 0.206 | 0.077 |
| 57 MN587683_TA529-B_rodent_Lophuromys_machangui_Tanzania_2013 | 0.636 | 0.493 | 0.535 | 0.58 | 0.552 | 0.517 | 0.587 | 0.605 | 0.563 | 0.497 | 0.57 | 0.493 | 0.51 | 0.552 | 0.552 | 0.549 | 0.51 | 0.58 | 0.493 | 0.538 | 0.556 | 0.5 | 0.49 | 0.566 | 0.528 | 0.538 | 0.51 | 0.514 | 0.528 | 0.566 | 0.559 | 0.552 | 0.563 | 0.521 | 0.545 | 0.479 | 0.486 | 0.483 | 0.472 | 0.462 | 0.455 | 0.462 | 0.444 | 0.465 | 0.465 | 0.514 | 0.514 | 0.538 | 0.5 | 0.465 | 0.444 | 0.448 | 0.322 | 0.21 | 0.084 | 0.059 |
| 58 MN587662_CRT64-B_rodent_Lophuromys_dudui_DRC_2010 | 0.647 | 0.493 | 0.531 | 0.57 | 0.559 | 0.503 | 0.591 | 0.612 | 0.549 | 0.503 | 0.556 | 0.493 | 0.51 | 0.552 | 0.556 | 0.552 | 0.514 | 0.58 | 0.5 | 0.531 | 0.563 | 0.493 | 0.497 | 0.57 | 0.517 | 0.521 | 0.503 | 0.521 | 0.531 | 0.577 | 0.566 | 0.556 | 0.566 | 0.521 | 0.538 | 0.49 | 0.465 | 0.479 | 0.465 | 0.462 | 0.451 | 0.462 | 0.427 | 0.462 | 0.462 | 0.497 | 0.503 | 0.538 | 0.49 | 0.455 | 0.434 | 0.434 | 0.329 | 0.224 | 0.077 | 0.063 | 0.066 |
| 59 MN587670_MOZ329-B_rodent_Lophuromys_machangui_Mozambique_201 | 0.64 | 0.469 | 0.538 | 0.573 | 0.563 | 0.497 | 0.584 | 0.619 | 0.556 | 0.49 | 0.545 | 0.493 | 0.521 | 0.549 | 0.552 | 0.556 | 0.521 | 0.57 | 0.507 | 0.545 | 0.566 | 0.503 | 0.493 | 0.556 | 0.521 | 0.524 | 0.517 | 0.521 | 0.531 | 0.573 | 0.573 | 0.552 | 0.559 | 0.51 | 0.549 | 0.462 | 0.476 | 0.479 | 0.472 | 0.469 | 0.455 | 0.465 | 0.451 | 0.469 | 0.476 | 0.486 | 0.503 | 0.528 | 0.483 | 0.451 | 0.448 | 0.43 | 0.322 | 0.196 | 0.073 | 0.063 | 0.07 | 0.066 |
| 60 MN587666_CRT74_rodent_Lophuromys_dudui_DRC_2010 | 0.636 | 0.493 | 0.535 | 0.577 | 0.563 | 0.507 | 0.584 | 0.612 | 0.552 | 0.497 | 0.552 | 0.5 | 0.517 | 0.559 | 0.559 | 0.556 | 0.507 | 0.573 | 0.497 | 0.545 | 0.559 | 0.493 | 0.5 | 0.563 | 0.535 | 0.538 | 0.51 | 0.524 | 0.528 | 0.559 | 0.559 | 0.556 | 0.552 | 0.517 | 0.552 | 0.472 | 0.465 | 0.472 | 0.472 | 0.469 | 0.451 | 0.469 | 0.441 | 0.465 | 0.469 | 0.493 | 0.5 | 0.521 | 0.486 | 0.458 | 0.434 | 0.444 | 0.329 | 0.217 | 0.077 | 0.052 | 0.066 | 0.059 | 0.059 |

Table S7a: Estimates of Evolutionary Divergence between Sequences, NS3 region
