# Supplementary material for: Molecular detection and genomic characterization of diverse hepaciviruses in African rodents
Source: Virus Evol. 2021 Apr 12;7(1):veab036. doi: 10.1093/ve/veab036 (PMC8242229; doi:10.1093/ve/veab036)
Supplement: veab036_Supplementary_Data [file veab036_supplementary_data.zip › Table_S8_R1.docx]

**Supplementary table S8:** Complete list of all rodent hepacivirus co-infections identified in this study.

| **Accession**  **number** | **Specimen voucher** | **Co-infections** | **Host family** | **Host species** | **Country** | **Sampling year** |
| --- | --- | --- | --- | --- | --- | --- |
| MN587650 | CRT125 | CRT125, strain 1 | Muridae | *Lophuromys dudui* | DRC | 2010 |
| MN587651 |  | CRT125, strain 2 | Muridae | *Lophuromys dudui* | DRC | 2010 |
| MN587652 |  | CRT125, strain 3 | Muridae | *Lophuromys dudui* | DRC | 2010 |
| MN587653 |  | CRT125, strain 4 | Muridae | *Lophuromys dudui* | DRC | 2010 |
| MN587654 | CRT352 | CRT352, strain 1 | Muridae | *Lophuromys dudui* | DRC | 2010 |
| MN587655 |  | CRT352, strain 2 | Muridae | *Lophuromys dudui* | DRC | 2010 |
| MN587658 | CRT490 | CRT490, strain 1 | Muridae | *Lophuromys dudui* | DRC | 2010 |
| MN587659 |  | CRT490, strain 2 | Muridae | *Lophuromys dudui* | DRC | 2010 |
| MN587660 |  | CRT490, strain 3 | Muridae | *Lophuromys dudui* | DRC | 2010 |
| MN587661 | CRT64 | CRT64, strain 1 | Muridae | *Lophuromys dudui* | DRC | 2010 |
| MN587662 |  | CRT64, strain 2 | Muridae | *Lophuromys dudui* | DRC | 2010 |
| MN587663 |  | CRT64, strain 3 | Muridae | *Lophuromys dudui* | DRC | 2010 |
| MN587664 |  | CRT64, strain 4 | Muridae | *Lophuromys dudui* | DRC | 2010 |
| MN587665 |  | CRT64, strain 5 | Muridae | *Lophuromys dudui* | DRC | 2010 |
| MN587669 | MOZ329 | MOZ329, strain 1 | Muridae | *Lophuromys machangui* | Mozambique | 2011 |
| MN587670 |  | MOZ329, strain 2 | Muridae | *Lophuromys machangui* | Mozambique | 2011 |
| MN587671 |  | MOZ329, strain 3 | Muridae | *Lophuromys machangui* | Mozambique | 2011 |
| MN587696 | TA100 | TA100, strain 1 | Muridae | *Lophuromys stanleyi* | Tanzania | 2013 |
| MN587697 |  | TA100, strain 2 | Muridae | *Lophuromys stanleyi* | Tanzania | 2013 |
| MN587698 |  | TA100, strain 3 | Muridae | *Lophuromys stanleyi* | Tanzania | 2013 |
| MN587673 | TA293 | TA293, strain 1 | Muridae | *Lophuromys laticeps* | Tanzania | 2013 |
| MN587674 |  | TA293, strain 2 | Muridae | *Lophuromys laticeps* | Tanzania | 2013 |
| MN587675 |  | TA293, strain 3 | Muridae | *Lophuromys laticeps* | Tanzania | 2013 |
| MN587676 |  | TA293, strain 4 | Muridae | *Lophuromys laticeps* | Tanzania | 2013 |
| MN587677 |  | TA293, strain 5 | Muridae | *Lophuromys laticeps* | Tanzania | 2013 |
| MN587678 | TA498 | TA498, strain 1 | Muridae | *Lophuromys machangui* | Tanzania | 2013 |
| MN587679 |  | TA498, strain 2 | Muridae | *Lophuromys machangui* | Tanzania | 2013 |
| MN587680 |  | TA498, strain 3 | Muridae | *Lophuromys machangui* | Tanzania | 2013 |
| MN587682 | TA529 | TA529, strain 1 | Muridae | *Lophuromys machangui* | Tanzania | 2013 |
| MN587683 |  | TA529, strain 2 | Muridae | *Lophuromys machangui* | Tanzania | 2013 |
| MN587684 |  | TA529, strain 3 | Muridae | *Lophuromys machangui* | Tanzania | 2013 |
| MN587685 |  | TA529, strain 4 | Muridae | *Lophuromys machangui* | Tanzania | 2013 |
| MN587686 | TA531 | TA531, strain 1 | Muridae | *Lophuromys machangui* | Tanzania | 2013 |
| MN587687 |  | TA531, strain 2 | Muridae | *Lophuromys machangui* | Tanzania | 2013 |
| MN587688 |  | TA531, strain 3 | Muridae | *Lophuromys machangui* | Tanzania | 2013 |
| MN587689 |  | TA531, strain 4 | Muridae | *Lophuromys machangui* | Tanzania | 2013 |
| MN587690 |  | TA531, strain 5 | Muridae | *Lophuromys machangui* | Tanzania | 2013 |
| MN587691 | TA532 | TA532, strain 1 | Muridae | *Lophuromys machangui* | Tanzania | 2013 |
| MN587692 |  | TA532, strain 2 | Muridae | *Lophuromys machangui* | Tanzania | 2013 |
| MN587693 |  | TA532, strain 3 | Muridae | *Lophuromys machangui* | Tanzania | 2013 |
| MN587694 |  | TA532, strain 4 | Muridae | *Lophuromys machangui* | Tanzania | 2013 |
