# Supplementary material for: Molecular detection and genomic characterization of diverse hepaciviruses in African rodents
Source: Virus Evol. 2021 Apr 12;7(1):veab036. doi: 10.1093/ve/veab036 (PMC8242229; doi:10.1093/ve/veab036)
Supplement: veab036_Supplementary_Data [file Table_S7b_R1_PDF.pdf › Table_S7b_R1_PDF.pdf]

	1	2	3	4	5	6	7	8	9	10	11	12	13	14	15	16	17	18	19	20	21	22	23	24	25	26	27	28	29	30
1 MG599990_fish/rept/turtle_Gymnothorax_reticularis_China																														
2 MG334001_fish/rept/turtle_Trachemys_scripta_USA_2008	0.714																													
3 MK737640_duck_Anas_platyrhynchos_China_2018	0.729	0.617																												
4 MN133813_mosquito_Culex_annulirostris_Australia_2018	0.752	0.624	0.489																											
5 MN062427_eagle_Haliaeetus_leucocephalus_USA_2002	0.737	0.699	0.444	0.489																										
6 MG599993_fish/rept/turtle_Protopterus_annectens_Nigeria	0.684	0.639	0.586	0.579	0.602																									
7 MG599988_fish/rept/turtle_Goniurosaurus_luii_China	0.722	0.632	0.526	0.586	0.549	0.564																								
8 MG599987_fish/rept/turtle_Teratoscincus_roborowskii_China	0.752	0.594	0.489	0.511	0.534	0.556	0.444																							
9 MG599989_fish/rept/turtle_Hemidactylus_bowringii_China	0.729	0.632	0.526	0.564	0.541	0.549	0.361	0.429																						
10 MG599991_fish/rept/turtle_Rhinobatos_hynnicephalus_China	0.707	0.586	0.586	0.609	0.594	0.526	0.511	0.564	0.489																					
11 MG599992_fish/rept/turtle_Urolophus_aurantiacus_China	0.744	0.632	0.594	0.586	0.594	0.586	0.556	0.579	0.549	0.489																				
12 KP641127_cattle_Bos_taurus_Germany_2014	0.669	0.586	0.594	0.602	0.594	0.504	0.571	0.564	0.617	0.564	0.579																			
13 KC551800_primate_Colobus_guereza_Uganda_2010	0.707	0.617	0.541	0.602	0.594	0.496	0.534	0.534	0.556	0.556	0.571	0.466																		
14 MH370348_rodent_Oligoryzomys_nigripes_Brazil_2012	0.722	0.677	0.526	0.579	0.534	0.466	0.556	0.556	0.564	0.534	0.586	0.451	0.451																	
15 KC411806_rodent_Rhabdomys_pumilio_South_Africa_2008	0.662	0.639	0.556	0.571	0.496	0.496	0.534	0.549	0.549	0.541	0.556	0.444	0.421	0.293																
16 KY370092_rodent_Meriones_meridianus_China_2014	0.684	0.639	0.549	0.564	0.519	0.504	0.541	0.526	0.586	0.534	0.594	0.459	0.481	0.301	0.293															
17 AF179612_primate_Saguinus_mystax_South_America_1995	0.722	0.662	0.602	0.594	0.564	0.624	0.594	0.586	0.594	0.564	0.647	0.534	0.496	0.481	0.466	0.481														
18 MF775364_shrew_Suncus_murinus_China_2015	0.722	0.639	0.564	0.602	0.564	0.564	0.579	0.556	0.586	0.549	0.579	0.466	0.346	0.429	0.436	0.459	0.489													
19 KC796077_bat_Hipposideros_vittatus_Kenya_2010	0.714	0.617	0.579	0.594	0.594	0.586	0.549	0.586	0.579	0.586	0.609	0.534	0.504	0.489	0.489	0.526	0.504	0.526												
20 MN635449_possum_Trichosurus_vulpecula_Sydney	0.662	0.639	0.556	0.602	0.571	0.511	0.564	0.526	0.564	0.556	0.564	0.436	0.459	0.368	0.376	0.398	0.496	0.436	0.541											
21 KJ950939_rodent_Rattus_norvegicus_USA_2012	0.654	0.662	0.586	0.586	0.586	0.504	0.571	0.526	0.579	0.534	0.564	0.391	0.421	0.429	0.414	0.459	0.504	0.436	0.504	0.353										
22 MH824542_sifaka_Propithecus_diadema_Madagascar_2013	0.714	0.662	0.586	0.586	0.586	0.496	0.602	0.594	0.609	0.541	0.632	0.421	0.451	0.429	0.436	0.451	0.526	0.414	0.511	0.376	0.353									
23 MG211815_rodent_Spermophilus_dauricus_China_2015	0.684	0.617	0.534	0.564	0.549	0.489	0.586	0.504	0.579	0.556	0.586	0.414	0.414	0.331	0.383	0.406	0.451	0.368	0.451	0.346	0.316	0.361								
24 MN564789_CRT682_rodent_Graphiurus_cf._kelleni_DRC_2010	0.707	0.654	0.609	0.609	0.586	0.444	0.541	0.556	0.564	0.504	0.579	0.406	0.421	0.368	0.406	0.421	0.496	0.414	0.496	0.323	0.286	0.346	0.293							
25 MN587695_TA085_rodent_Lophuromys_stanleyi_Tanzania_2013	0.669	0.647	0.571	0.609	0.556	0.541	0.579	0.534	0.617	0.511	0.602	0.391	0.459	0.451	0.444	0.474	0.504	0.414	0.534	0.376	0.301	0.353	0.323	0.338						
26 MN587668_MOZ133_rodent_Lophuromys_machangui_Mozambique_2011	0.662	0.624	0.564	0.617	0.564	0.534	0.571	0.541	0.617	0.519	0.594	0.406	0.436	0.459	0.429	0.489	0.519	0.414	0.549	0.383	0.301	0.376	0.338	0.346	0.038					
27 KJ950938_rodent_Rattus_norvegicus_USA_2013	0.684	0.639	0.594	0.579	0.564	0.459	0.564	0.534	0.594	0.534	0.571	0.406	0.429	0.421	0.398	0.459	0.429	0.421	0.474	0.368	0.293	0.376	0.271	0.301	0.301	0.308				
28 KY370095_rodent_Dipus_sagitta_China_2014	0.692	0.617	0.586	0.586	0.594	0.534	0.556	0.549	0.594	0.534	0.594	0.398	0.444	0.406	0.429	0.459	0.444	0.429	0.511	0.346	0.271	0.361	0.286	0.248	0.278	0.308	0.286			
29 MN555567_TZ25757_rodent_Acomys_wilsoni_Tanzania_2011	0.729	0.647	0.586	0.602	0.571	0.474	0.549	0.556	0.617	0.549	0.594	0.421	0.383	0.414	0.436	0.436	0.474	0.414	0.519	0.338	0.323	0.361	0.286	0.271	0.301	0.301	0.248	0.271		
30 MG822666_rodent_Proechimys_semispinosus_Panama_2014	0.684	0.647	0.556	0.586	0.564	0.526	0.549	0.504	0.586	0.594	0.594	0.414	0.436	0.406	0.414	0.451	0.474	0.429	0.481	0.338	0.331	0.398	0.316	0.353	0.293	0.323	0.316	0.301	0.308	
31 MN564790_ETH674_rodent_Stenocephalemys_albipes_Ethiopia_2012	0.684	0.617	0.594	0.586	0.564	0.481	0.571	0.541	0.624	0.526	0.602	0.376	0.414	0.383	0.414	0.436	0.421	0.406	0.481	0.331	0.278	0.338	0.263	0.271	0.256	0.278	0.211	0.241	0.226	0.256
32 MG600413_rodent_Rhizomys_pruinosus_Vietnam_2015	0.677	0.647	0.571	0.586	0.586	0.549	0.564	0.511	0.624	0.564	0.586	0.451	0.436	0.421	0.421	0.459	0.459	0.406	0.489	0.346	0.316	0.368	0.278	0.331	0.278	0.286	0.316	0.286	0.323	0.278
33 KC815310_rodent_Peromyscus_maniculatus_USA_2008	0.669	0.617	0.564	0.609	0.579	0.489	0.556	0.541	0.609	0.534	0.594	0.391	0.421	0.398	0.414	0.451	0.474	0.414	0.511	0.368	0.286	0.383	0.293	0.293	0.263	0.256	0.263	0.278	0.241	0.256
34 KY370094_rodent_Neodon_clarkei_China_2014	0.684	0.624	0.586	0.594	0.571	0.504	0.556	0.511	0.609	0.534	0.602	0.391	0.414	0.429	0.383	0.421	0.459	0.406	0.459	0.353	0.278	0.376	0.301	0.263	0.278	0.286	0.256	0.248	0.241	0.263
35 KC411784_rodent_Myodes_glareolus_Netherlands_2007	0.699	0.647	0.579	0.602	0.579	0.549	0.579	0.526	0.624	0.586	0.609	0.436	0.406	0.451	0.429	0.466	0.474	0.414	0.489	0.376	0.286	0.376	0.278	0.316	0.286	0.301	0.286	0.263	0.248	0.256
36 KC796074_bat_Hipposideros_vittatus_Kenya_2011	0.699	0.647	0.617	0.617	0.571	0.586	0.556	0.594	0.609	0.571	0.624	0.541	0.526	0.534	0.459	0.534	0.564	0.534	0.579	0.571	0.519	0.526	0.481	0.541	0.541	0.541	0.526	0.526	0.549	0.541
37 KC796078_bat_Otomops_martiensseni_Kenya_2011	0.722	0.639	0.579	0.602	0.571	0.564	0.571	0.571	0.556	0.549	0.594	0.519	0.474	0.436	0.391	0.436	0.526	0.474	0.534	0.474	0.474	0.466	0.421	0.459	0.481	0.466	0.436	0.474	0.481	0.459
38 KP325401_horse_Equus_ferus_USA_2011	0.707	0.632	0.639	0.632	0.617	0.632	0.617	0.639	0.639	0.632	0.632	0.609	0.526	0.549	0.541	0.519	0.586	0.541	0.549	0.579	0.571	0.579	0.534	0.564	0.579	0.586	0.586	0.556	0.556	0.579
39 EF108306_human_Homo_sapiens	0.714	0.617	0.632	0.624	0.647	0.602	0.647	0.647	0.677	0.624	0.617	0.647	0.571	0.579	0.549	0.541	0.609	0.541	0.571	0.564	0.556	0.564	0.534	0.571	0.586	0.571	0.564	0.579	0.571	0.602
40 NC_009823_human_Homo_sapiens	0.722	0.632	0.662	0.624	0.639	0.617	0.639	0.632	0.662	0.639	0.647	0.654	0.586	0.586	0.571	0.549	0.602	0.549	0.571	0.586	0.594	0.586	0.556	0.586	0.609	0.602	0.556	0.594	0.579	0.624
41 NC_009824_human_Homo_sapiens	0.729	0.669	0.647	0.632	0.632	0.632	0.684	0.639	0.654	0.632	0.639	0.662	0.586	0.594	0.556	0.571	0.609	0.571	0.579	0.579	0.571	0.564	0.556	0.571	0.579	0.571	0.564	0.579	0.579	0.624
42 NC_009826_human_Homo_sapiens	0.744	0.639	0.647	0.609	0.602	0.609	0.669	0.632	0.647	0.609	0.624	0.647	0.571	0.586	0.556	0.541	0.617	0.556	0.571	0.579	0.586	0.571	0.564	0.586	0.624	0.617	0.586	0.617	0.617	0.632
43 NC_009827_human_Homo_sapiens	0.714	0.654	0.632	0.624	0.617	0.617	0.647	0.617	0.654	0.617	0.624	0.624	0.571	0.556	0.541	0.519	0.586	0.541	0.526	0.571	0.556	0.556	0.519	0.549	0.564	0.564	0.564	0.564	0.571	0.594
44 NC_009825_human_Homo_sapiens	0.714	0.669	0.632	0.647	0.617	0.594	0.654	0.632	0.639	0.609	0.647	0.639	0.579	0.579	0.556	0.556	0.594	0.549	0.586	0.571	0.556	0.564	0.549	0.564	0.579	0.571	0.579	0.571	0.586	0.609
45 M62321_HCV_reference	0.737	0.632	0.639	0.609	0.639	0.639	0.669	0.624	0.647	0.624	0.654	0.662	0.594	0.594	0.579	0.571	0.609	0.556	0.564	0.602	0.602	0.602	0.571	0.579	0.617	0.617	0.594	0.609	0.609	0.632
46 MG599997_fish/rept/turtle_Chimaera_sp_China	0.759	0.639	0.564	0.541	0.541	0.511	0.534	0.556	0.504	0.481	0.489	0.571	0.579	0.526	0.541	0.541	0.564	0.586	0.549	0.586	0.541	0.586	0.556	0.549	0.579	0.571	0.571	0.571	0.571	0.541
47 MG599996_fish/rept/turtle_Chimaera_sp_China	0.759	0.617	0.504	0.541	0.556	0.519	0.519	0.526	0.489	0.451	0.496	0.579	0.571	0.534	0.564	0.549	0.579	0.564	0.571	0.624	0.519	0.556	0.541	0.564	0.571	0.571	0.564	0.564	0.586	0.526
48 MG599995_fish/rept/turtle_Squalus_brevirostris_China	0.752	0.647	0.556	0.579	0.602	0.579	0.571	0.534	0.541	0.504	0.504	0.586	0.586	0.549	0.564	0.549	0.602	0.609	0.571	0.564	0.549	0.632	0.586	0.579	0.586	0.586	0.586	0.564	0.571	0.556
49 MG599994_fish/rept/turtle_Mustelus_manazo_China	0.759	0.571	0.586	0.579	0.624	0.579	0.571	0.564	0.602	0.519	0.459	0.564	0.556	0.579	0.564	0.594	0.602	0.564	0.617	0.594	0.534	0.594	0.519	0.556	0.556	0.549	0.564	0.534	0.541	0.564
50 MG599999_fish/rept/turtle_Pelodiscus_sinensis_China	0.707	0.617	0.534	0.541	0.564	0.511	0.579	0.579	0.571	0.579	0.579	0.549	0.534	0.511	0.459	0.504	0.549	0.534	0.519	0.564	0.526	0.571	0.511	0.541	0.549	0.526	0.541	0.549	0.534	0.519
51 MG600000_fish/rept/turtle_Mauremys_megalocephala_China	0.729	0.677	0.564	0.519	0.549	0.571	0.571	0.534	0.586	0.602	0.571	0.549	0.564	0.489	0.489	0.504	0.549	0.549	0.526	0.534	0.541	0.541	0.496	0.571	0.534	0.541	0.556	0.534	0.549	0.511
52 KY370091_rodent_Allactaga_sibirica_China_2014	0.684	0.609	0.541	0.609	0.579	0.496	0.579	0.556	0.564	0.541	0.579	0.541	0.444	0.444	0.451	0.519	0.549	0.429	0.474	0.459	0.474	0.496	0.444	0.459	0.481	0.459	0.489	0.459	0.451	0.474
53 KC411777_rodent_Myodes_glareolus_Germany_2010	0.722	0.639	0.564	0.541	0.549	0.474	0.579	0.541	0.594	0.549	0.556	0.481	0.504	0.429	0.481	0.489	0.579	0.474	0.526	0.519	0.504	0.541	0.459	0.466	0.481	0.481	0.481	0.504	0.474	0.504
54 MN564792_TA132_rodent_Praomys_jacksoni_Tanzania_2013	0.699	0.609	0.526	0.564	0.526	0.519	0.556	0.526	0.594	0.549	0.541	0.459	0.481	0.444	0.466	0.519	0.556	0.466	0.541	0.496	0.466	0.511	0.436	0.459	0.451	0.436	0.481	0.474	0.459	0.489
55 MN587672_TA275_rodent_Lophuromys_laticeps_Tanzania_2013	0.669	0.647	0.564	0.579	0.534	0.511	0.541	0.556	0.594	0.564	0.579	0.459	0.481	0.421	0.429	0.489	0.564	0.451	0.526	0.459	0.444	0.496	0.406	0.444	0.436	0.444	0.444	0.459	0.444	0.436
56 MN587655_CRT352-B_rodent_Lophuromys_dudui_DRC_2010	0.692	0.647	0.571	0.579	0.519	0.511	0.534	0.556	0.602	0.571	0.579	0.474	0.474	0.406	0.436	0.489	0.556	0.451	0.549	0.459	0.451	0.489	0.406	0.436	0.436	0.436	0.451	0.459	0.421	0.451
57 MN587683_TA529-B_rodent_Lophuromys_machangui_Tanzania_2013	0.677	0.647	0.564	0.586	0.519	0.511	0.549	0.564	0.594	0.571	0.564	0.481	0.466	0.406	0.429	0.496	0.549	0.444	0.549	0.451	0.444	0.481	0.406	0.429	0.429	0.429	0.451	0.451	0.421	0.436
58 MN587662_CRT64-B_rodent_Lophuromys_dudui_DRC_2010	0.684	0.639	0.564	0.579	0.511	0.519	0.541	0.564	0.594	0.571	0.579	0.489	0.481	0.414	0.436	0.496	0.556	0.444	0.549	0.451	0.436	0.481	0.406	0.436	0.436	0.436	0.451	0.466	0.421	0.444
59 MN587670_MOZ329-B_rodent_Lophuromys_machangui_Mozambique_201	0.677	0.639	0.564	0.579	0.526	0.519	0.541	0.564	0.586	0.564	0.564	0.489	0.466	0.421	0.421	0.496	0.549	0.436	0.549	0.451	0.444	0.489	0.414	0.444	0.436	0.429	0.459	0.459	0.429	0.444
60 MN587666_CRT74_rodent_Lophuromys_dudui_DRC_2010	0.677	0.639	0.564	0.586	0.526	0.511	0.549	0.564	0.594	0.571	0.571	0.481	0.474	0.414	0.421	0.496	0.541	0.436	0.549	0.444	0.444	0.481	0.406	0.436	0.429	0.429	0.451	0.451	0.421	0.436

	31	32	33	34	35	36	37	38	39	40	41	42	43	44	45	46	47	48	49	50	51	52	53	54	55	56	57	58	59
32 MG600413_rodent_Rhizomys_pruinosus_Vietnam_2015	0.271																												
33 KC815310_rodent_Peromyscus_maniculatus_USA_2008	0.173	0.301																											
34 KY370094_rodent_Neodon_clarkei_China_2014	0.203	0.233	0.218																										
35 KC411784_rodent_Myodes_glareolus_Netherlands_2007	0.218	0.256	0.256	0.173																									
36 KC796074_bat_Hipposideros_vittatus_Kenya_2011	0.541	0.526	0.549	0.504	0.489																								
37 KC796078_bat_Otomops_martiensseni_Kenya_2011	0.459	0.466	0.451	0.429	0.489	0.368																							
38 KP325401_horse_Equus_ferus_USA_2011	0.579	0.564	0.594	0.556	0.564	0.489	0.489																						
39 EF108306_human_Homo_sapiens	0.579	0.541	0.586	0.549	0.579	0.534	0.519	0.331																					
40 NC_009823_human_Homo_sapiens	0.594	0.571	0.602	0.579	0.594	0.549	0.541	0.353	0.18																				
41 NC_009824_human_Homo_sapiens	0.609	0.541	0.602	0.586	0.586	0.556	0.526	0.353	0.188	0.226																			
42 NC_009826_human_Homo_sapiens	0.617	0.579	0.624	0.594	0.594	0.541	0.549	0.353	0.226	0.203	0.226																		
43 NC_009827_human_Homo_sapiens	0.586	0.549	0.594	0.579	0.579	0.519	0.511	0.331	0.195	0.18	0.158	0.218																	
44 NC_009825_human_Homo_sapiens	0.579	0.571	0.594	0.571	0.602	0.541	0.534	0.368	0.218	0.226	0.218	0.233	0.248																
45 M62321_HCV_reference	0.609	0.594	0.602	0.609	0.617	0.534	0.549	0.376	0.226	0.195	0.241	0.226	0.188	0.226															
46 MG599997_fish/rept/turtle_Chimaera_sp_China	0.571	0.549	0.571	0.556	0.571	0.579	0.541	0.609	0.609	0.609	0.647	0.624	0.609	0.624	0.602														
47 MG599996_fish/rept/turtle_Chimaera_sp_China	0.564	0.549	0.534	0.556	0.564	0.519	0.504	0.609	0.609	0.632	0.639	0.624	0.602	0.639	0.632	0.263													
48 MG599995_fish/rept/turtle_Squalus_brevirostris_China	0.564	0.556	0.541	0.549	0.556	0.632	0.579	0.639	0.617	0.602	0.639	0.609	0.609	0.632	0.602	0.406	0.444												
49 MG599994_fish/rept/turtle_Mustelus_manazo_China	0.541	0.541	0.534	0.526	0.526	0.594	0.556	0.624	0.564	0.617	0.617	0.602	0.617	0.617	0.632	0.474	0.459	0.459											
50 MG599999_fish/rept/turtle_Pelodiscus_sinensis_China	0.519	0.519	0.511	0.504	0.519	0.564	0.526	0.609	0.579	0.602	0.602	0.602	0.602	0.602	0.609	0.511	0.489	0.556	0.594										
51 MG600000_fish/rept/turtle_Mauremys_megalocephala_China	0.534	0.504	0.556	0.549	0.519	0.556	0.519	0.594	0.586	0.624	0.617	0.632	0.602	0.609	0.639	0.511	0.534	0.564	0.579	0.248									
52 KY370091_rodent_Allactaga_sibirica_China_2014	0.466	0.474	0.481	0.466	0.436	0.534	0.541	0.579	0.549	0.579	0.586	0.594	0.579	0.549	0.602	0.489	0.534	0.549	0.541	0.398	0.451								
53 KC411777_rodent_Myodes_glareolus_Germany_2010	0.474	0.504	0.504	0.474	0.474	0.564	0.541	0.594	0.594	0.617	0.624	0.579	0.602	0.586	0.624	0.504	0.526	0.526	0.526	0.414	0.451	0.338							
54 MN564792_TA132_rodent_Praomys_jacksoni_Tanzania_2013	0.459	0.459	0.459	0.451	0.444	0.549	0.549	0.579	0.594	0.617	0.617	0.602	0.594	0.579	0.624	0.519	0.519	0.519	0.511	0.421	0.444	0.316	0.173						
55 MN587672_TA275_rodent_Lophuromys_laticeps_Tanzania_2013	0.451	0.459	0.459	0.459	0.421	0.549	0.511	0.556	0.586	0.586	0.624	0.609	0.594	0.586	0.624	0.526	0.541	0.541	0.526	0.421	0.429	0.331	0.18	0.203					
56 MN587655_CRT352-B_rodent_Lophuromys_dudui_DRC_2010	0.421	0.444	0.444	0.444	0.406	0.556	0.519	0.556	0.571	0.571	0.609	0.602	0.594	0.571	0.617	0.526	0.549	0.541	0.519	0.421	0.436	0.323	0.195	0.211	0.053				
57 MN587683_TA529-B_rodent_Lophuromys_machangui_Tanzania_2013	0.429	0.444	0.444	0.451	0.406	0.541	0.504	0.549	0.571	0.579	0.594	0.602	0.586	0.571	0.617	0.526	0.549	0.541	0.519	0.429	0.436	0.316	0.195	0.203	0.053	0.038			
58 MN587662_CRT64-B_rodent_Lophuromys_dudui_DRC_2010	0.429	0.451	0.444	0.444	0.398	0.541	0.534	0.564	0.579	0.579	0.609	0.609	0.586	0.579	0.617	0.519	0.549	0.541	0.519	0.436	0.451	0.331	0.188	0.203	0.06	0.053	0.038		
59 MN587670_MOZ329-B_rodent_Lophuromys_machangui_Mozambique_201	0.436	0.459	0.451	0.459	0.414	0.541	0.511	0.556	0.571	0.586	0.602	0.609	0.586	0.571	0.617	0.519	0.541	0.541	0.519	0.429	0.436	0.323	0.195	0.211	0.053	0.068	0.03	0.03	
60 MN587666_CRT74_rodent_Lophuromys_dudui_DRC_2010	0.429	0.451	0.444	0.451	0.406	0.541	0.511	0.556	0.579	0.586	0.609	0.609	0.586	0.579	0.617	0.526	0.549	0.541	0.519	0.436	0.436	0.323	0.195	0.211	0.038	0.045	0.023	0.038	0.023

Table S7b: Estimates of Evolutionary Divergence between Sequences, NS5B region
